# Pyrrole Compounds from the Two-Step One-Pot Conversion of 2,5-Dimethylfuran for Elastomer Composites with Low Dissipation of Energy

**DOI:** 10.3390/molecules29040861

**Published:** 2024-02-15

**Authors:** Simone Naddeo, Davide Gentile, Fatima Margani, Gea Prioglio, Federica Magaletti, Maurizio Galimberti, Vincenzina Barbera

**Affiliations:** Department of Chemistry, Materials and Chemical Engineering “G. Natta”, Politecnico di Milano, Via Mancinelli 7, 20131 Milano, Italy; simone.naddeo@polimi.it (S.N.); davide.gentile@polimi.it (D.G.); fatima.margani@polimi.it (F.M.); gea.prioglio@polimi.it (G.P.); federica.magaletti@polimi.it (F.M.)

**Keywords:** green chemistry, circular, almost null E-factor, 2,5-dimethylfuran, pyrrole compounds

## Abstract

A one-pot, two-step process was developed for the preparation of pyrrole compounds from 2,5-dimethylfuran. The first step was the acid-catalyzed ring-opening reaction of 2,5-dimethylfuran (DF), leading to the formation of 2,5-hexanedione (HD). A stoichiometric amount of water and a sub-stoichiometric amount of sulfuric acid were used by heating at 50 °C for 24 h. Chemically pure HD was isolated, with a quantitative yield (up to 95%), as revealed by ^1^H-NMR, ^13^C-NMR, and GC-MS analyses. In the second step, HD was used as the starting material for the synthesis of pyrrole compounds via the Paal–Knorr reaction. Various primary amines were used in stoichiometric amounts. ^1^H-NMR, ^13^C-NMR, ESI-Mass, and GC-Mass analyses confirmed that pyrrole compounds were prepared with very good/excellent yields (80–95%), with water as the only co-product. A further purification step was not necessary. The process was characterized by a very high carbon efficiency, up to 80%, and an E-factor down to 0.128, whereas the typical E-factor for fine chemicals is between 5 and 50. Water, a co-product of the second step, can trigger the first step and therefore make the whole process circular. Thus, this synthetic pathway appears to be in line with the requirements of a sustainable chemical process. A pyrrole compound bearing an SH group (SHP) was used for the functionalization of a furnace carbon black (CB). The functionalized CB (CB/SHP) was utilized in place of silica, resulting in a 15% mass reduction of reinforcing filler, in an elastomeric composite based on poly(styrene-co-butadiene) from solution anionic polymerization and poly(1,4-cis-isoprene) from *Hevea Brasiliensis*. Compared to the silica-based composite, a reduction in the Payne effect of about 25% and an increase in the dynamic rigidity (E’ at 70 °C) of about 25% were obtained with CB/SHP.

## 1. Introduction

Responsible consumption and production and climate action are among the sustainable development goals of the United Nations [1]. To achieve these goals, the replacement of fossil resources with renewable biomass for the production of chemicals has emerged as a major research topic [2,3,4,5,6,7]. Lignocellulosic biomass has an estimated annual production of 180 billion metric tons [4]; hence, it is an almost endless reservoir.

Among the chemicals derived from lignocellulosic sources, a strategic role is played by furanic compounds [8,9,10,11,12,13,14,15,16,17,18,19]. In particular, furfural and 5-hydroxymethylfurfural are versatile chemical platforms [16,19] and give rise to a great variety of downstream substances, such as 2,5-dimethylfuran (DF). DF is indeed prepared through the hydrogenolysis of 5-hydroxymethylfurfural [20], apart from direct synthesis from fructose by means of tandem dehydration/hydrogenolysis [21,22,23]. 

DF has been prevailingly investigated for conversion to p-xylene [24,25]. Few other reactions have been reported, including conversion to hydrodeoxygenated products [26], to chiral oxyndoles [27], and to 2,5-hexanedione (HD), which is an intermediate for a wide range of synthetic processes and is largely used in the chemical industry with great commercial value. 

The use of bio-sources in place of oil to produce HD is of growing interest. HD has been prepared through the Pd/C-catalyzed hydrogenation of 5-hydroxymethylfurfural (HMF) in water and under CO_2_ atmosphere at high pressure (30–40 bar) and high temperature (150 °C) [28]. Analogously, the hydrogenation of 5-hydroxymethylfurfural (HMF) in ethanol at 140 °C by using an iridium complex as a catalyst led to 1-hydroxyhexan-2,5-dione [29]. Scientific papers were published on the acid-catalyzed ring opening of DF for the synthesis of HD. The reactions were performed in pure D_2_O at 250 °C for 30 min [30] in the presence of a cationic resin and an excess of H_2_O (H_2_O:DF = 3:1) at room temperature for 72 h [31]; in the presence of 10% sulfuric acid in H_2_O and glacial acetic acid at 85 °C [32]; and in the presence of 10% by mole of a strong acid in H_2_O and 1/1 mixtures of H_2_O and an organic solvent at T = 60–80 °C [33]. Moreover, the ring-opening reaction of DF is achieved by using sulfuric acid and ruthenium catalysts in 2-propanol at 80–90 °C [34]. The use of the acid is thus mandatory for the ring-opening reaction of DF. However, it was reported that prolonged contact of HD with the acidic media led to the formation of oligomeric products [35]. Hence, the methods reported in the literature for the preparation of HD from DF present some drawbacks, such as the use of organic solvents and/or an excess of water; high temperatures; and, in particular, in view of scaling up on an industrial scale, the formation of by-products.

The synthesis of a pyrrole compound (PyC) via the Paal–Knorr reaction of primary amine and 1,4-dicarbonyl compounds was performed in neat conditions by using different synthetic approaches, such as microwaves [36] or mechanochemistry in the presence of organic acids [37]. Pyrrole derivatives were also obtained by using flow chemistry methods [38], from microscale to production scale. With the aim of scaling up the process, some of the authors developed the neat Paal–Knorr reaction by simply mixing and heating primary amines and HD in the absence of solvent(s) and catalysts, achieving high atom efficiency [39,40,41]. The PyCs were used for the neat functionalization of sp^2^ carbon allotropes [41,42] and inorganic oxy-hydroxides such as silica [43]. 

The research on PyCs was focused on two families of compounds, *Janus* molecules [44,45] bearing either hydroxy- or sulfur-based functional groups. Both families of PyCs had a twofold reactivity, based on the pyrrole ring and on the substituent of the nitrogen atom. The pyrrole ring promoted the functionalization of the sp^2^ carbon allotropes through a domino reaction (shown in Figure S1 in the Supplementary Materials) with the carbocatalytic oxidation of PyC in the benzylic position, followed by the cycloaddition reaction of PyC with the graphitic substrate [45]. This functionalization reaction was characterized by the absence of co- and by-products, by yields ranging from 60% to 90% in the case of graphene layers [39,40], and, thus, by high carbon efficiency. The most studied PyC, with hydroxy functional groups in the substituent of the nitrogen atom, was 2-(2,5-dimethyl-1*H*-pyrrol-1-yl)-1,3-propanediol (serinol pyrrole, SP), whose chemical structure is reported below in the text [39,41]. The hydroxy groups promote the condensation reaction of SP with silica with excellent/quantitative yield without the need for solvents or catalysts [42]. The most investigated PyCs with sulfur-based functional groups were 2-(2,5-dimethyl-1*H*-pyrrol-1-yl)ethane-1-thiol (SHP) and 1,2-bis(2-(2,5-dimethyl-1*H*-pyrrol-1-yl)ethyl)disulfide (SSP). Their chemical structure is reported below in the text. The -SH and -SS- functional groups were reported to react with the unsaturated elastomers [46]. The functionalization of silica and of sp^2^ carbon allotropes, mainly carbon black, with the two mentioned families of PyCs improved the properties of elastomeric composites for a large-scale application such as that in tire compounds [46,47]. The development on an industrial scale was announced by a major player in the tire field [48]. 

The objectives of the research here reported were to develop a sustainable synthesis of PyCs, with a view of large-scale production, and to demonstrate that a pyrrole compound obtained from such a synthesis makes a significant contribution to the sustainability of a large-scale product such as a tire. Therefore, one of the objectives of the research was to carry out a one-pot synthesis in which HD was synthesized from DF. The obtained HD was then used as the starting material in reactions with different primary amines, achieving excellent/quantitative yield and no waste, thus aiming at very high carbon efficiency and very low E-factor [49,50,51]. In particular, experimental conditions for the ring-opening reaction of DF had to be suitable to allow the efficient reaction of HD with the primary amine, avoiding the formation of by-products. It is worth noting that the synthesis of a PyC leads to water as the co-product. The use of an excess of water for the preparation of HD from DF, as documented in the above-mentioned literature, would be detrimental to this equilibrium reaction. Moreover, an excess of acid could lead to the formation of oligomeric products, based not only on HD but also on the pyrrole compound, as reported in the literature [35]. 

In this work, the synthesis of pyrrole compounds was performed in two steps: (i) ring-opening reaction of DF to HD, and (ii) ring closure of HD to form the pyrrole rings, through the reaction with a primary amine. Several primary amines were investigated.

**Figure 1:**
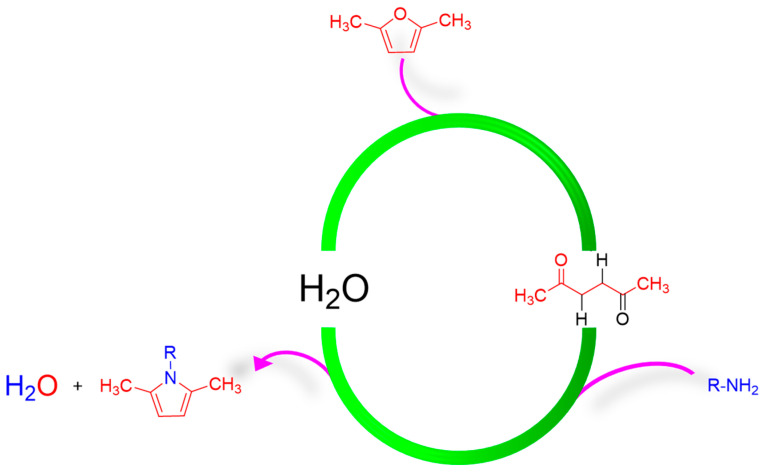
The water cycle for the synthesis of pyrrole compounds in a two-step, one-pot process. The valorization of all the atoms involved is also underlined.

Water is the nucleophile that allows the ring opening of the furan ring and it is also the co-product of the Paal–Knorr reaction. These characteristics make the process circular. The scheme in Figure 1 shows that the cyclic process promoted by water leads to the formation of PyC having 100% carbon economy. In the synthesis here reported, solvents were not used, in line with the basic rules of green chemistry [49,50,51]. Hence, a quantitative yield is the only necessary condition to have high carbon efficiency and low E-factor [51]. 

A further objective of this work was to use a pyrrole compound prepared through the one-pot two-step process in an elastomeric composite characterized by low dissipation of energy and also suitable for large-scale tire application. As mentioned above, SP was used as a coupling agent for silica in composites suitable for tire compounds [42]. It is well-known that silica is the filler of choice [52,53,54,55] for the preparation of low-dissipation-energy tires because silica elastomer composites are characterized by low hysteresis. However, silica-based composites have several technical drawbacks. Firstly, the silanols of the silica surface lead to high compound viscosity, short storage time, difficult processability, corrosion, abrasion of metal surfaces and need of special mixing equipment [56]. On the other hand, silica silanols are abl to establish a chemical bond with the elastomer chains, through the use of coupling agent. Generally, a sulphur-based silane such as bis(triethoxysilylpropyl)tetrasulfide (TESPT) is used for this aim. However, TESPT increases the adhesiveness of the composites to the metal parts of the equipment, which require special treatments. Moreover, the condensation reaction of TESPT with the silanols of silica releases ethanol. The combustion of ethanol produces CO_2_. Serinol Pyrrole (SP) was used in place of TESPT. As a matter of fact, the use of SP would allow us to avoid the release of about 8.0 × 10^7^ Kg of CO_2_ on a worldwide basis [43]. Furthermore, it would be highly desirable to reproduce the properties of a silica-based elastomer composite, without using silica as the filler and without using a coupling agent for the filler. 

Hence, the objective of the research here reported was to replace silica by using a functionalized furnace carbon black (CB in the following phrases) with a pyrrole compound. The pyrrole compound selected for the functionalization of CB was SHP, obtained from cysteamine as the primary amine. The procedure used for the functionalization of CBN234 was already reported [46]. The aim was to reproduce the dynamic mechanical properties of the silica-based composite. The elastomers were a solution-polymerized poly(styrene-co-butadiene) (S-SBR) and poly(1,4-cis-isoprene) from *Hevea brasiliensis* (natural rubber, NR), typically used in a tire tread. The hypothesis behind the use of CB/SHP was to exploit the chemical reactivity of the thiol group with the unsaturations of the elastomer chains. The ultrafast thiol-ene reaction based on cysteine has been reported [57,58] and the reaction of thiol groups with internal double bonds has been documented [59]. In a recent paper [46], some of the authors showed the ability of SHP to react with squalene, used as a model compound of the unsaturated elastomer. The increase in the dynamic rigidity of the elastomer composite, by using CB/SHP in place of CB [46], was attributed to the chemical bond between CB and the elastomer chains. In this work, for the first time, CB/SHP was used in place of silica with the aim to prepare an elastomer composite for low dissipation of energy. The composites were prepared by means of melt blending in an internal mixer and were cured with a sulfur-based system. The cross-linking reaction was studied, and the dynamic mechanical properties in the shear and in the axial mode were determined.

## 2. Results and Discussion

### 2.1. Synthesis of Pyrrole Compounds

A one-pot two-step process was performed for the synthesis of pyrrole compounds starting from DF. The first step was the conversion of DF (**1**) into HD (**2**) and the second step was the ring closure to the pyrrole compound (**3**), via the Paal–Knorr reaction of HD with a primary amine. 

**Figure 2:**
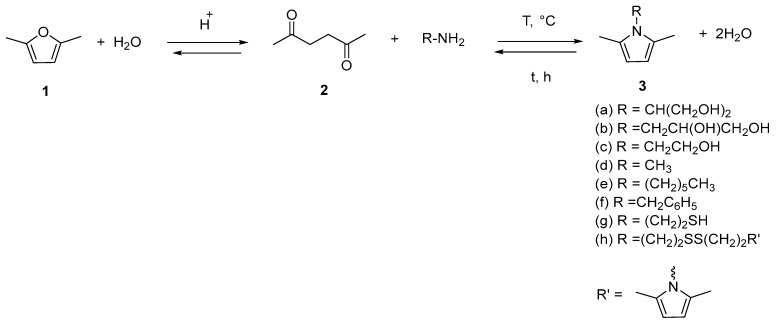
The one-pot, two-step synthetic process that yields variously substituted pyrroles (**3a**–**h**) with almost null E-factor.

As shown in Figure 1 and Figure 2, the ring-opening reaction of DF to HD and the ring-closing reaction to pyrrole are both water-dependent processes.

*Step 1. Ring-opening reaction of DF to HD.*

The ring-opening reaction of DF to HD is a reaction without co-products, hence with 100% atom economy. It is an acid-catalyzed equilibrium reaction that requires at least a stoichiometric amount of water to ensure the formation of HD. As the conversion of DF to HD is a neat reaction, two considerations can be made. (i) The reaction is triggered by water and acid, with the proton transfer from the protonated water to the furan ring. It takes place at room temperature, with the heterocycle insoluble in water. (ii) As the furan is converted to HD, water is consumed. Therefore, there is no water in the reaction mixture, at the end of the first step. Investigation of (a) the amount of water and (b) the amount and the type of acid was carried out to assess their effect on the yield of the reaction. Data are given in Table 1 and details are in the experimental section.

*(a) Effect of the amount of water.* Firstly, the effect of water was investigated by using 15 mol% of H_2_SO_4_ (pKa = −3) with respect to DF. This amount of acid was to be considered the limit value to avoid the formation of by-products, in particular of the HD oligomers, documented in the literature with 16 mol % of H_2_SO_4_ [35]. 

Entry 1 was carried out with an excess of water (H_2_O/DF = 3/1). Entries 2–4 were carried out with a decreasing amount of water (about 30% less for each entry), reaching an equimolar amount of H_2_O and DF in Entry 4, without observing a decrease in the yield to HD, which was quantitative. It was observed that a slightly lower yield was achieved with the largest amount of water. Without going too far into inferences not supported by experimental results, it could be said that an excess of water favors the reverse reaction, since water acts as a proton transfer agent that could hinder the direct reaction. The ^1^H-NMR spectra of Entries 1, 2, 3, and 4 are shown in Figures S2–S7 respectively, in the Supplementary Material. Residual water was detected in the spectra of the reaction mixtures, when an amount of water larger than the stoichiometric one was used. The quantitative yield of HD obtained with a DF/H_2_O molar ratio = 1 is a relevant result for the one-pot process, since water is the co-product of the Paal–Knorr reaction, and its presence should be avoided at the beginning of the reaction.

*(b) Amount and type of acid.* Moving from the experimental conditions of Entry 4, the amount of sulfuric acid was reduced from 4 to 1.7 and to 0.1 (mol% with respect to DF) (Table 1, Entries 5, 6, and 7, respectively). The yield was almost quantitative by also using 4% of H_2_SO_4_, whereas it dropped to low values when the amount of mineral acid was further reduced, at 17% and 13%, respectively. ^1^H-NMR spectra of the final reaction mixtures of Entries 6 and 7 are reported in Figures S8 and S9, respectively, in the Supplementary Material. To account for these results, the interaction between the H_3_O^+^ from the acids and the basic centers in DF and H_2_O could be considered. In 2,5-dimethylfuran, one of the two sp^2^ lone pair electrons is available for reacting with protons, while the other one is involved in the heterocyclic aromatic system. When mineral acid is added to the reaction mixture, it reacts with the lone pair of the oxygen atom in the water molecule, which is more basic. In the reaction mixtures of Entries 6 and 7, with the low amount of H_3_O^+^, water was preferentially protonated and the ring opening of DF occurred only to a very minor extent. Acids with different pK_a_ values, higher and lower than the pKa of H_2_SO_4_, were examined, keeping constant their amount equal to 4 mol% with respect to DF. The following acids were tested: HBr (pKa = −9), HCl (pKa = −8.08), HNO_3_ (pKa = −1.3), and CH_3_COOH (pKa = 4.88). Data are in Entries 8–11 in Table 1. Reactions performed with strong mineral acid, like hydrochloric and hydrobromic acid, allowed us to convert DF to HD with quantitative yield (Entries 8–9 of Table 1). Reaction performed in the presence of nitric acid (Entry 10 in Table 1) led to the formation of a number of by-products, whose chemical nature was not assessed. HD was not obtained with acetic acid (Entry 11 in Table 1). These findings suggest the usage of a strong mineral acid for the protonation of the DF ring, which leads, consequently, to the ring opening and then to the conversion to HD. Highly oxidizing acid should be avoided. All the ^1^H-NMR spectra of Entries 8, 9, 10, and 11 are reported in Figures S10–S12, respectively, in the Supplementary Material.

Based on these experimental findings, HD for the reaction with a primary amine (Step 2 of the process) was prepared with the following experimental conditions: molar ratio DF/H_2_O = 1/1 (47.0 mmol each), 4 mol% of H_2_SO_4_, 50 °C, 24 h. The kinetics of the ring-opening reaction, in these conditions, was studied by means of ^1^H-NMR spectroscopies of the reaction mixture, analyzing samples at increasing times: 2, 3, 3.5, 5, 8, and 24 h. The NMR spectra are in Figure S2 in the Supplementary Material. Most of the reaction occurred after 8 h, as shown by the substantial decrease in the intensity of the methyl group in the NMR spectrum in Figure S2 and by the curves in the graph in Figure S3. Nonetheless, 24 h was adopted as the reaction time and co- or by-products were not observed.

*Step 2. Paal–Knorr reaction of primary amines with HD derived from DF.*

The reaction scheme for the synthesis of the pyrrole compounds is in Figure 2. 

The following primary amines were used: 2-amino-1,3-propandiol (serinol), 1-amino-2,3-propandiol (isoserinol), ethanolamine, methylamine, hexanamine, benzylamine, cysteamine, and cystamine. HD and the acid catalyst were present in the reaction mixture at the end of the first step. The amine was then added to the mixture without any further purification. This approach mimics the settings of a traditional Paal–Knorr reaction, apart from the absence of solvent. The reactions are characterized by very good atom economy (from 75% to 90%), the only co-product being water, which is, however, the reagent of the first step of the process. The detailed procedures are reported in the experimental section. In brief, neat reactions were carried out in temperatures ranging from 25 °C to 155 °C for 2–2.5 h. Data are in Table 2. The yield of all the reactions performed with the primary amines was at least very good (≥80%), and in some cases was excellent (>90%). The atom efficiency was from 61% (Entry 1) to 77% (Entry 5) and the carbon efficiency of the whole two-step process was from 74% (Entry 8) to 94% (Entry 4). Low values of E-factor were obtained, from 0.8 to 0.13. Complete characterization of pyrroles **3a**–**3h** is available in the Supplementary Material. ^1^H-NMR spectra and GC-Mass chromatograms are from Figures S13–S37 (for the detailed list, see the Supplementary Material). 

Primary amines are also available as hydrochlorides. To check the possibility of using the hydrochlorides in the one-pot two-step process, the synthesis of SHP and SSP was also performed from cysteamine hydrochloride and cystamine dihydrochloride. Sodium acetate was added to release the free amino group of cysteamine and cystamine from the hydrochloride salt. Good yields were obtained, from 73% to 78%, according to the principles of Green Metrics [49,50,51]. The lower atom economy and reaction mass efficiency are due to the use of sodium acetate. Hence, the process based on the hydrochloride salts, though feasible, is not advisable to pursue sustainability. The synthesis and characterization of SHP and SSP from hydrochloride salts are reported in the Supplementary Materials.

In previous works by some of the authors, pyrrole compounds were prepared by using oil-derived HD. The yields were worse in the case of 1-hexyl-2,5-dimethyl-*1H*-pyrrole (73%) [40] and 1,2,5-Trimethylpyrrole (86.9%) [42] and better in the case of 2-(2,5-dimethyl-1*H*-pyrrol-1-yl)propane-1,3-diol (96%) [40] (the water in the serinol sample used in the present work affected the yield). Hence, the process here reported at least does not make the reaction yield worse.

### 2.2. Elastomer Composites with CB/SHP as the Reinforcing Filler

As reported in the introduction, the CB/SHP adduct was used in place of silica in an elastomer composite suitable for tire tread. Silica is the preferred filler in tire tread elastomer composites with low dissipation of energy, thanks to its reactivity with the elastomer chains, mediated by the sulfur-based silane. The objective of this study was to replace silica with a functionalized carbon black, CB/SHP, in an elastomer matrix based on S-SBR, whose vinyl groups were reported to react with CB/SHP. The preparation and characterization of the CB/SHP adduct used in this work has been recently reported [46]. Silica was used as the reinforcing filler in the reference composite. The composites based on either silica or CB/SHP contained the same volume amount of filler (silica and CB). The use of CB allowed us to save 15% by mass of the reinforcing filler. Reference composites were prepared as well with pristine CB and the same vulcanization system of the silica-based composite (“CB” composite) and with the same amount of sulfur atoms present in the CB/SHP composite (“CB + S” composite). The composites were prepared via melt blending in an internal mixer, by using the same traditional mixing procedure as reported in the experimental section.

#### Crosslinking

**Figure 3:**
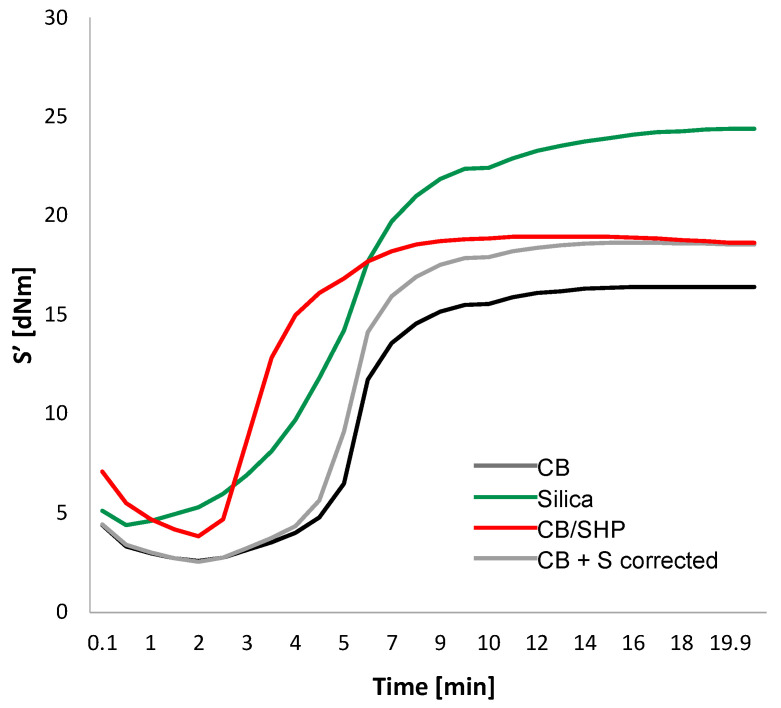
Curing curves of the elastomer composites. The recipes are reported in the experimental section.

The crosslinking was performed with a sulfur-based system. Details are in the experimental section. The rheometric curves are in Figure 3 and data of M_L_, M_H_, (M_H_ − M_L_), t_s1_, t_90_, and curing rate are in Table S1, in the Supplementary Material.

The replacement of silica with CB led to a reduction in the composite viscosity, as indicated by the M_L_ values, to a lower extent with CB/SHP. The M_H_ and (M_H_ − M_L_) values, which can be correlated with both the crosslinking and the filler networks [46], were remarkably higher in the presence of silica and appear to depend on the amount of sulfur atoms for the composites containing CB; in fact, they are higher for the (CB/SHP) and (CB+ S) composites. The induction time of vulcanization (t_s1_) was lower for the (silica) composite, whereas the optimum time (t_90_) was lower for the CB-based composites, and the highest curing rate was for the composite with CB/SHP. 

The lower viscosity of the CB-based composite was expected, in consideration of the lower surface activity of CB, and is in line with the objectives of this work. The higher M_L_ value obtained with CB/SHP suggests that CB/SHP could react with the vinyl groups of S-SBR during the processing. The higher M_H_ and (M_H_ − M_L_) values of the (silica) composite can be explained, at least in part, by the formation of the filler network. The higher curing efficiency of the CB-based composites is due to the replacement of silica with the carbonaceous filler and the higher curing rate observed with CB/SHP can be explained by the reactivity of the thiol group with the elastomer chains. 

### 2.3. Dynamic Mechanical Properties in the Shear Mode

**Figure 4:**
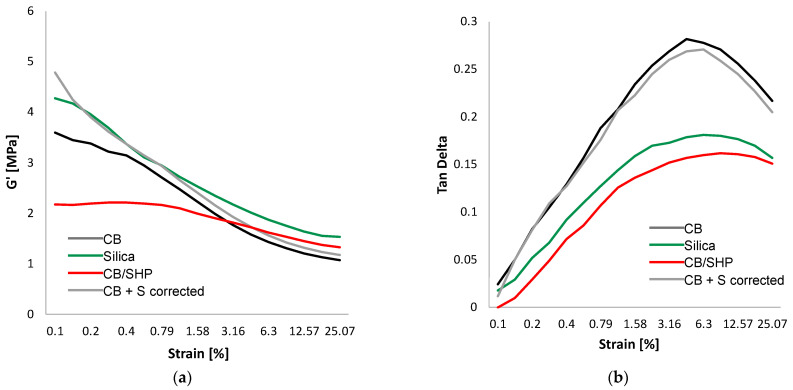
G’ vs. strain amplitude (**a**) and Tan Delta versus strain amplitude (**b**) for composites of Table 1.

Dynamic mechanical properties were determined, for the crosslinked samples, in the shear mode by means of strain sweep experiments, by using a strain amplitude in the range from 0.1% to 25% at a frequency of 1 Hz and a temperature of 50 °C. Details are in the experimental section. The storage G’ and the loss G’’ moduli were measured. The graphs showing the dependence on the strain amplitude of G’ and of Tan delta (G”/G’ ratio) are in Figure 4a,b, respectively, and data of G’γ_min_, G’γ_max_, ΔG’, ΔG’/G’γ_min_, G’’_max_, and Tan δ_max_ are in Table S2 in the Supplementary Material. G’ at minimum strain is an index of the presence of the filler network. The ΔG’ and ΔG’/G’γ_min_ values were taken as the indexes of the non-linearity of the modulus, which is known as the Payne effect [60,61,62,63]. 

**Figure 5:**
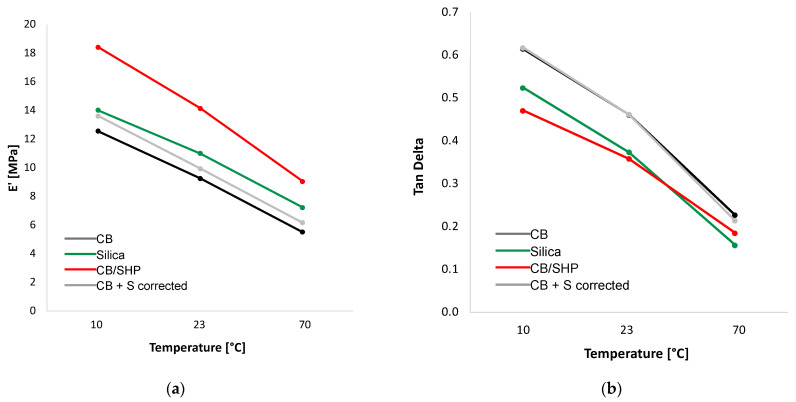
(**a**) E’ vs. temperature curves and (**b**) Tan Delta vs. temperature curves for the composites whose ricepise is reported in Table 4 in the experimental Part.

With CB/SHP, remarkably lower values of G’γ_min_, ΔG’/G’γ_min_, G’’_max_, and Tan δ_max_ were obtained. The reduction, compared to the (silica) composite and to the (CB + S) composite, is indeed appreciable. In particular, the reduction in ΔG’/G’γ_min_ was about 25% and 33%, respectively. It is worth observing in Figure 5a the crossover of the curves of the (CB) and (CB/SHP) composites; the curve due to the (CB/SHP) composite is well below the other curves at minimum strain and is above them at high strain. These findings indicate that the difference between the (silica) composite and the (CB/SHP) and (CB + S) composites is not due to the amount of sulfur in the composites. It can be thus commented that CB/SHP reacts with the elastomer chains, and this brings about the reduction in the filler network. 

### 2.4. Dynamic Mechanical Properties in the Axial Mode

Axial dynamic mechanical analyses in compression were carried out by first applying a pre-strain of 25%, then a dynamic sinusoidal strain of 3.5% at a frequency of 100 Hz. Storage modulus (E’) and loss modulus (E’’) were measured at 10 °C, 23 °C, and 70 °C. Data are reported in Table 3 and the dependence of E’ and Tan Delta on the temperature is shown in Figure 5a,b, respectively.

CB/SHP led to higher dynamic rigidity with respect to all the other composites, at all the temperatures. E’ at 70 °C of the (CB/SHP) composite was higher than E’ of the (silica) and the (CB + S) composites of about 25% and 46%, respectively. It is worth observing that the (E’_70 °C_ − E’_10 °C_) difference is similar for the (silica) and the (CB/SHP) composite. This is a very meaningful finding, as the reduction in the dynamic rigidity with temperature is a very important feature for a tire tread compound. The tan delta values of the CB/SHP composite are lower at 10 °C and 23 °C and similar at 70 °C, compared to the values of the (silica) composite. The values of E’ are lower and the values of Tan delta are higher for the (CB + S) composite. Hence, the content of sulfur in the CB-based composites is not the parameter which steers the dynamic mechanical properties of the composites. These results can be reasonably justified by the reaction of CB/SHP with the elastomer chains. It is indeed worth adding that a higher dynamic rigidity gives the opportunity to reduce the filler content, thus reducing the hysteresis of the composite. In a recent work [46], it was shown that CB-SHP is more efficient than CB-SSP in promoting the dynamic-mechanical reinforcement of the elastomer composite. For example, it was shown that the dynamic rigidity increased more than 20% and the hysteresis decreased by about 10%, at 70 °C, by using CB/SHP and CB/SSP in place of 33% and 66% of CB, respectively. It was hypothesized that only one of the two pyrrole rings of CB-SSP could react with the carbon substrate. 

The recipes of the elastomer composites are reported in Table 4 in the experimental section.

The results obtained with CB/SHP demonstrate the key role of CB functionalization and are very important for improving the sustainability of elastomer composites. In fact, carbon black can rightly be considered not an example of sustainability, but new carbon fillers are appearing on the scene, such as char from tires [64] and biochar [65]. The low surface area and the lack of structure make these carbon materials unsuitable as tire compounds, and only the reactivity brought by functionalization can enable their use.

## 3. Experimental Section

### 3.1. Materials

#### 3.1.1. For the Preparation of Pyrrole Compounds

Reagents and solvents, commercially available, were purchased and used without further purification.

Sulfuric acid 95–97% purity, acetic acid solution, hexylamine, methylamine solution (40% in weight), ethanolamine, benzylamine, cysteamine, and cystamine were purchased from Sigma Aldrich. Hydrochloric acid solution (37% w) and nitric acid solution (69.5% w) were purchased from Carlo Erba. Bromidic acid solution (48% w) was purchased from Fluka. 2-Amino-1,3-propanediol (serinol) and 1-amino-2,3-propandiol (isoserinol) were kindly provided by Bracco.

#### 3.1.2. For the Preparation of CB/SHP Adduct

CBN234 with a carbon content not lower than 98 wt%, a surface area of 113 m^2^/g, an average elemental particle size of 27 nm, and an average aggregate size of 152 nm was from Cabot Corporation, Ravenna, Italy.

#### 3.1.3. For the Preparation of Rubber Composites

Solution styrene-butadiene rubber (S-SBR) was poly (styrene-co-butadiene) from anionic polymerization promoted by an organo-lithium initiator: SPRINTAN SLR-4630 (TRINSEO), with 25% styrene, 37.5 parts of TDAE oil, typical glass transition temperature = −28 °C, Mooney viscosity (ML(1 + 4)100 °C) = 55 MU.

Natural rubber was poly(1,4-cis-isoprene) from Hevea brasiliensis: STR20 (Eatern GR Thailand − Chonburi). 

Silica ZEOSIL 1165MP (Solvay). The product was in the form of white micropearls. The specific surface area was 140−180 m^2^/g, loss on drying (2 h @ 105 °C) ≤ 8.0%, soluble salts (as Na_2_SO_4_) ≤ 2.0%. 

CBN234 was of the same commercial grade reported above. 

Stearic acid was provided by Sogis (Sospiro, Italy), ZnO was provided by Zincol Ossidi (Ferrania, Italy), N′-phenyl-p-phenylenediamine (6PPD) was provided by Crompton (Mumbai, India), sulfur was provided by Solfotecnica (Ravenna, Italy), and N-ter-butyl-2-benzothiazyl sulfenamide (TBBS) was provide by Flexsys (St. Louis, MO, USA).

#### 3.1.4. Synthesis of Pyrrole Derivatives

It was performed in one pot and two steps.

*Step 1. General procedure for the synthesis of 2,5-hexanedione (2) (STEP 1)*

The procedure adopted for Entry 1 of Table 1 is reported as follows. The same procedure was followed for the other entries, except that the amount of water and/or the amount of acid were changed. The water in the reaction partially came from the acid solution. 

In a round-bottomed flask, equipped with a condenser, distilled water (47.0 mmol, 0.85 mL), sulfuric acid (4 mol%, 1.88 mmol), and 2,5-dimethylfuran (47.0 mmol, 5.0 mL) were put in sequence. Then, the oil bath was set at 50 °C and the reaction was stirred for 24 h. The final mixture was analyzed by ^1^H-NMR spectroscopy. 2,5-hexanedione (HD) (**2**) was obtained as a dark brown liquid with a yield of 95% and used without any further purification in STEP 2. Water was detected in negligible traces. The relative yield of 2,5-hexanedione and the amount of the unreacted 2,5-dimethylfuran were determined by ^1^H-NMR spectroscopy, considering the ratio of the signals of DF and HD, respectively.

*Step 2. General procedure for the synthesis of pyrrole compounds*

*Reaction of 2,5-HD with primary amines*

The selected primary amine, either serinol (38.0 mmol), isoserinol (47.0 mmol), ethanolamine (47.0 mmol), methylamine solution (40% in weight water solution) (47.0 mmol), hexylamine (47.0 mmol), benzylamine (47.0 mmol), cysteamine (47.0 mmol), or cystamine (23.5 mmol), was introduced in a 50 mL round-bottomed flask, containing the 2,5-hexanedione (**2**), obtained in Step 1, in equimolar ratio. The mixtures were stirred for 2 h at different temperatures (indicated below). The chemical purity of the products was determined by means of ^1^H-NMR, ^13^C-NMR, and GC-Mass spectroscopies. The reaction yield was calculated considering the final amount (mmol) of the pyrrole compound compared to the starting amount of 2,5-dimethylfuran (mmol). 

The coupling constants and chemical shift values from ^1^H-NMR and ^13^C-NMR analyses of the PyC prepared in this work have been compared with literature values. The data are in Tables S1 and S2, respectively, in the Supplementary Material. The values are very similar when the analyses are performed in the same solvent. The use of different solvents led to different values.

*2-(2,5-dimethyl-1H-pyrrol-1-yl)propane-1,3-diol (Serinol pyrrole) (***3a***)*

Reaction temperature: 150 °C. Dark viscous red liquid was obtained: 6.73 g, 80% yield. 

Atom Economy (A.E_c_) = 82%; Atom efficiency (A.E_f_) = 65%, Reaction Mass Efficiency(RME) = 72%, E-Factor = 0.097.

^1^H-NMR (*d_6_*-DMSO), δ, (ppm): 5.55 (s, 2H), 4.78 (t, J = 5.5 Hz, 2H), 4.09 (m, J = 6.7 Hz, 1H), 3.76 (dt, J_1_ = 11.5 Hz, J_2_ = 6.0 Hz, 2H), 3.64 (dt, J_1_ = 11.1 Hz, J_2_ = 7.1 Hz, J_3_ = 5.5 Hz, 2H), 2.15 (s, 6H). ^13^C-NMR (CDCl3), δ, (ppm): 128.97, 107.09, 62.61, 60.30, 14.15

GC-Mass: retention time = 17.309 min; molecular peak = 169 *m*/*z.*

ESI-Mass: [M − H]^−^: Calculated exact mass = 168.11 *m*/*z*; found exact mass = 168.0 *m*/*z.*

*3-(2,5-dimethyl-1H-pyrrol-1-yl)propane-1,2-diol (iso-Serinol pyrrole) (***3b***)*

Reaction temperature: 50 °C. Viscous red liquid was obtained: 8.60 g, 92% yield.

A.E_c_ = 82%; A.E_f_ = 75%, RME = 89%, E-Factor = 0.084.

^1^H-NMR (*d_6_*-DMSO), δ, (ppm): 5.57 (s, 2H), 4.82 (s, 1H), 4.69 (s, 1H), 3.85 (dd, J_1_ = 18.0 Hz, J_2_ = 7.30 Hz 1H), 3.58 (dd, 2H), 3.31 (dd, 2H), 2.14 (s, 6H). ^13^C-NMR (CDCl_3_), δ, (ppm): 128.44, 105.74, 71.71, 64.12, 46.21, 12.82.

GC-Mass: retention time = 17.064 min; molecular peak = 169 *m*/*z.*

ESI-Mass: [M + H]^+^: Calculated exact mass = 170.11 *m*/*z*; found exact mass = 170.2 *m*/*z*, 

*2-(2,5-dimethyl-1H-pyrrol-1-yl)ethanol (ethanol pyrrole, EP) (***3c***)*

Reaction temperature: 155 °C. Dark brown viscous liquid was obtained: 6.27 g, 93% yield. 

A.E_c_ = 79%; A.E_f_ = 73%, RME = 76%, E-Factor = 0.101.

^1^H-NMR (*d_6_*-DMSO), δ, (ppm): 5.58 (s, 2H, CH-pyr), 4.83 (s, J = 5.3 Hz, 1H, OH), 3.77. (t, J = 6.5 Hz, 2H, CH_2_), 3.49 (m, J = 6.1 Hz, 2H, CH_2_), 2.14 (s, 6H, CH_3_). 

^13^C-NMR (CDCl3), δ, (ppm): 128.17, 105.57, 62.24, 45.67, 12.72.

GC-Mass: retention time = 13.973 min; molecular peak = 139 *m*/*z.*

ESI-Mass: [M + Na]^+^: Calculated exact mass = 162.31 *m*/*z*; found exact mass = 162.3 *m*/*z*.

*1,2,5-trimethyl-1H-pyrrole (trimethyl pyrrole, TMP) (***3d***)*

Reaction temperature: 25 °C. Dark brown liquid was obtained: 4.97 g, 94% yield. 

A.E_c_ = 75%; A.E_f_ = 70%, RME = 72%. E-Factor = 0.128.

^1^H-NMR (CDCl_3_), δ, (ppm): 5.78, s, 2H; 3.39, s, 3H; 2.22, s, 6H. 

^13^C-NMR (CDCl_3_), δ, (ppm): 127.83, 104.70, 30.00, 12.55.

GC-Mass: retention time = 9.286 min; molecular peak = 109 *m*/*z.*

ESI-Mass: [2M + H]^+^: Calculated exact mass = 219.31 *m*/*z*; found exact mass [2M + H]^+^ = 219.2 *m*/*z*.

*1-hexyl-2,5-dimethyl-1H-pyrrole (hexyl pyrrole, HP) (***3e***)*

Reaction temperature: 60 °C. Dark brown liquid was obtained: 7.67 g, 85% yield. 

A.E_c_ = 90%; A.E_f_ = 76%, RME = 75.9%, E-Factor = 0.086.

^1^H-NMR (DMSO-*d_6_*), δ, (ppm): 5.58 (s, 2H), 3.68 (t, J = 15.6 Hz, 2H), 3.13 (s, 6H), 1.49 (m, 14.4 Hz, 2H), 1.31–1.25 (m, J = 14.0 Hz, 6H), 0.86 (t, J = 6.7 Hz, 3H). ^13^C-NMR (CDCl_3_), δ, (ppm): 127.41, 105.06, 43.81, 31.66, 31.14, 26.79, 22.71, 14.12, 12.60.

GC-Mass: retention time = 15.754 min; molecular peak = 179 *m*/*z.*

ESI-Mass: [2M + H]^+^: Calculated exact mass = 359.16 *m*/*z*; found exact mass = [2M + H] = 359.5 *m*/*z*. 

*1-benzyl-2,5-dimethyl-1H-pyrrole (benzyl pyrrole, BP) (***3f***)*

Reaction temperature: 100 °C. Dark brown viscous liquid was obtained: 6.93 g, 80% yield. 

A.E_c_ = 83.7%; A.E_f_ = 66%, RME = 75.9%, E-Factor = 0.088.

^1^H-NMR (DMSO-*d_6_*), δ, (ppm): 7.30 (m, J = 7.4 Hz, 2H, Ar benz), 7.22 (t, J = 7.3 Hz, 1H; Ar benz), 6.85 (d, J= 8.2 Hz, 2H; Ar benz), 5.71 (s, 2H, Ar pyr), 5.03 (s, 2H, CH_2_), 2,06 (s, 6H, CH_3_).

^13^C-NMR (CDCl_3_), δ, (ppm): 138.69, 128.82, 128.11, 127.11, 127.03, 125.78, 105.55, 46.85, 12.56.

GC-Mass: retention time = 17.291 min; molecular peak = 185 *m*/*z.*

ESI-Mass: [M + H]^+^: Calculated exact mass = 186.26 *m*/*z*; found exact mass = 186.3.

*2-(2,5-Dimethyl-1H-pyrrol-1-yl)ethane-1-thiol (SHP) (***3g***)*

Dark brown liquid was obtained: 5.31g. yield of 79%. 

A.E_c_ = 81%; A.E_f_ = 64%, RME = 65%, E-Factor = 0.076.

^1^H-NMR (CDCl_3_), δ (ppm): 5.78, (s, 2H, Ar), 3.93 (m, J = 7.75 Hz, 2H, -CH_2_), 2.75–2.66 (m, J_1_ = 7.7 Hz, J_2_ = 15.8 Hz, 2H, -CH_2_), 2.24 (s, 6H, -CH_3_), 1.36 (t, J = 8.5 Hz, 1H, -SH). 

^13^C-NMR (*d_6_*-DMSO), δ (ppm): 126.81, 104.74, 46.16, 24.09, 12.18.

GC-Mass analysis retention time = 14.50 min, molecular peak = 155 *m*/*z*.

*1,2-Bis(2-(2,5-dimethyl-1H-pyrrol-1-yl)ethyl)disulfide (SSP) (***3h***)*

A dark orange solid was obtained: 5.39 g, 74% yield. 

A.E_c_ = 65%; A.E_f_ = 48%, RME = 58%, E-Factor = 0.080.

^1^H-NMR (CDCl_3_), δ (ppm): 5.78 (s, 4H, Ar), 4.12–4.02 (m, J = 7.9 Hz 4H, CH_2_), 2.87–2.83 (m, J = 7.9 Hz 4H, CH_2_), 2.25 (s, 12H, CH_3_).

^13^C-NMR (CDCl_3_), δ (ppm): 127.43, 105.90, 43.22, 37.94, 12.64.

GC-Mass: retention time = 25.76 min; molecular peak = 308 *m*/*z*.

ESI-Mass: [M + Na]^+^: Calculated exact mass = 331.13 *m*/*z*; found exact mass = 331.2 *m*/*z*.

#### 3.1.5. Preparation and Characterization of the CB/PyC Adduct

The preparation and characterization of the CB/SHP adduct have been reported elsewhere [46]. In brief, CB and SHP were mixed with the help of acetone and sonication. Upon removing the solvent at reduced pressure, the physical mixture was heated at 150 °C for 2 h. The organic solvent and sonication were used at the lab scale to allow the preparation of a homogeneous mixture but were avoided at the pre-industrial scale, where a spray-dry can be used. TGA was carried out on the adduct, and the weight loss between 150 °C and 900 °C allowed us to estimate an amount of the modifier in the adduct equal to 7 parts per 100 parts of CB.

### 3.2. Preparation of Elastomeric Composites

#### 3.2.1. Recipes

The recipes of the elastomer composites are reported in Table 4. The amount of the ingredients is expressed in parts per hundred rubber (phr). In the text below, the composites are indicated with reference to the reinforcing filler, indicated in brackets. For example, the composite with CB/SHP is indicated as the (CB/SHP) composite.

#### 3.2.2. Mixing Procedure

**Figure 6:**
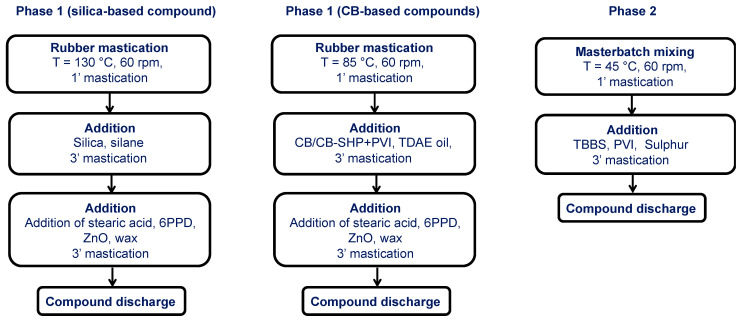
Block diagram of elastomeric composites reported in Table 1.

The composites were prepared via melt blending by using a Brabender^®^ internal mixer whose volume was 55 cc (Brabender^®^ PL-2000 Plasti-Corder Torque Rheometer, Brabender GmbH & Co. KG, Duisburg, Germany). The procedure is shown in the infographic in Figure 6.

### 3.3. Characterization Methods

#### 3.3.1. Characterization of Pyrrole Compounds

NMR spectra were recorded on a Bruker 400 MHz instrument (100 MHz) at 298 K. Chemical shifts were reported in ppm with the solvent residual peak as the internal standard (DMSO-*d_6_*: δH = 2.50 ppm; CDCl_3_: δH = 7.26 ppm). 

Mass spectra were recorded by using electrospray ionization (ESI) with a Bruker Esquire 3000 plus ion-trap mass spectrometer instrument equipped with an ESI Ion Trap LC/MSn System.

The instrument for GC-MS analysis was an Agilent 5973 (Agilient technologies, Santa Clara, CA, USA) network mass selective detector with a 6890 Series GC system mass spectrometer. The column used for all analyses was a J&W GC Column HP-5MS [(5%-phenyl)-methylpolysiloxane] 30 m, with 0.25 mm internal diameter and 0.25 μm film thickness.

#### 3.3.2. Characterization of CB/SHP Adduct

CB/SPH adduct was characterized as reported in reference [46].

#### 3.3.3. Characterization of Elastomer Composites

##### Crosslinking

A rubber process analyzer (RPA, Alpha Technologies, Hudson, OH, USA) was used for performing the crosslinking reaction of each rubber composite. A total of 0 g of elastomeric composites was placed in the rheometer. A first thermal ramp was performed on the sample in order to cancel the thermomechanical history. A strain sweep from 0.1−25% of strain amplitude at 50 °C was applied to non-crosslinked samples. Then, the sample was kept at 50 °C for 10 min, and another strain sweep at 50 °C was applied. Then, the temperature was increased from 50 °C to 170 °C, and the crosslinking reaction occurred. The crosslinking reaction was performed at 170 °C for 20 min. The oscillation angle adopted was 6.28° at a frequency of 1.7 Hz. The minimum torque (M_L_), the maximum torque (M_H_), the time needed to have a torque equal to ML + 1 dNm (t_S1_), and the time needed to reach 90% of the maximum torque (t_90_) were measured at the end of the analysis.

#### 3.3.4. Dynamic Mechanical Analyses in Shear Strain Sweep Tests

Shear dynamic mechanical properties were evaluated by using a rubber process analyzer (RPA). 

A strain sweep at low deformations (0.1–25% strain) was applied to the crude sample. 

The temperature then was kept at 50 °C for ten minutes and subjected to another strain sweep at 50 °C before being vulcanized. After 20 min at 50 °C, the crude elastomeric composites were subjected to a strain sweep of 0.1–25%. The frequency of the oscillation was 1 Hz. The measured properties were shear storage, G’, and loss moduli G’’.

#### 3.3.5. Dynamic Mechanical Characterization in the Axial Mode

An instron dynamic device was used for the determination of the dynamic mechanical properties in the traction compression mode according to the following methods. A test piece of the crosslinked elastomeric composition was preloaded up to 25%. The dimensions of the cylinder were as follows: length = 25 mm; diameter = 12 mm. The obtained cylinders were kept at the prefixed temperature (10, 23, and 70 °C) for the whole duration of the test. Longitudinal deformation with respect to the initial length was applied, and then the sample was submitted to a dynamic sinusoidal strain having an amplitude of ±3.5% with respect to the length under pre-load. The experimental data were measured at a frequency of 100 Hz. The dynamic mechanical properties are expressed in terms of dynamic storage modulus (E′) and loss factor (Tan Delta) values. The Tan Delta value is calculated as a ratio between loss (E”) and storage modulus (E′).

## 4. Conclusions

This work demonstrates that pyrrole compounds can be prepared via a one-pot, two-step synthesis, through the acid-catalyzed ring-opening reaction of a cellulosic derivative such as DF to HD, followed by the Paal–Knorr reaction of HD with a primary amine. In the process, the key role is played by water, the reagent in the ring opening of DF, and the co-product of the pyrrole synthesis. Water could thus create a circular process. The amount of water and acid in the first step was tuned to have a high yield in the second step and to avoid the formation of by-products. Excellent yield to HD was achieved with mild reaction conditions (50 °C and 8 h as the time needed to achieve most of the yield) by using a stoichiometric amount of water and a sub-stoichiometric amount of sulfuric acid. The formation of HD oligomers was prevented. HD was used without any purification for the Paal–Knorr reaction in the presence of different primary amines. Pyrrole compounds were obtained with good, very good, and excellent yields, without by-products. The chemical purity of the products of the first and second steps was confirmed by ^1^H-NMR, ^13^C-NMR, and GC-Mass spectroscopies. Pyrrole compounds can thus be prepared with excellent carbon efficiency and with an almost null E-factor.

Adduct of CB with SHP, the pyrrole compound prepared from cysteamine, was used in place of silica in elastomer composites, saving 15% by mass of reinforcing filler and obtaining a reduction in the Payne effect of about 25% and an increase in dynamic rigidity (E’ at 70 °C) of about 25%. The higher dynamic rigidity paves the way for the reduction in filler content, thus reducing hysteresis.

As mentioned in the introduction, a primary company in the tire field announced the use of pyrrole compounds as coupling agents for reinforcing fillers, both silica and carbon black. The global market for tires is forecast to reach 2.7 billion units by 2025. By estimating 10 kg as the total weight of the compounds in a tire, 30 as the weight% of the reinforcing filler, and 5% as the average amount of the coupling agent, 40 × 10^7^ kg of pyrrole compounds could be needed for the whole tire market, a remarkable amount that would indeed require a sustainable synthesis. By considering a more reasonable market penetration (the company that promotes this technology has a market share of about 5%), a tonnage of about 2 × 10^4^ tons could be estimated. This tonnage is in the typical range of fine chemicals, whose production is characterized by an E-factor of 5 (kg waste/kg product), as reported in the well-known table of E-factors. It is thus worth observing that the E-factor for the preparation of the pyrrole compounds here presented is much lower. The process reported in this manuscript appears to be in line with the teaching of green chemistry: “*what needed was not end-of-pipe remediation of waste but waste prevention at source by developing clear processes*”. The lower E-factor for the preparation of chemicals, the lower weight, the Payne effect, and the hysteresis of elastomeric composites for tire compounds can indeed make a remarkable contribution to the sustainability of a large-scale product such as a tire. Moreover, to implement the sustainability of the synthetic protocols, sources of energy, such as microwave or ultrasound irradiation, could be used.

## Figures and Tables

**Table 1:** Step 1 of the process. Synthesis of HD from DF and H_2_O in the presence of an acid.

Entry	Ingredient: Type and Amount				Yield (%)
	DF (mmol)	H_2_O (mmol)	Acid Catalyst		
			Type	mol % ^a^	
1	47	141	H_2_SO_4_^b^	15	95
2	47	94	H_2_SO_4_^b^	15	97
3	47	70	H_2_SO_4_^b^	15	99
4	47	47	H_2_SO_4_^b^	15	98
5	47	47	H_2_SO_4_^b^	4	95
6	47	47	H_2_SO_4_^b^	1.7	17
7	47	47	H_2_SO_4_^b^	0.1	13
8	47	47	HCl ^c^	4	97
9	47	47	HBr ^d^	4	97
10	47	47	HNO_3_^e^	4	<5
11	47	47	CH_3_COOH ^f^	4	0

^a^ with respect to DF; ^b^ 97% w; ^c^ 37% w; ^d^ 48% w; ^e^ 69.5% w; ^f^ glacial.

**Table 2:** Step 2 of the process: synthesis of pyrrole compounds from primary amines and HD from Step 1.

Entry	Primary Amine ^a^	T (°C)	Time (h)	Pyrrole Compound Yield	Carbon Efficiency ^b^	E-Factor ^d^
1 ^a^	Serinol	150	2.5	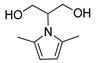 80%	80% ^c^	0.097
2	Isoserinol	50	2	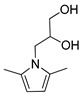 92%	92%	0.084
3	Ethanolamine	155	2	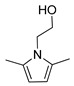 93%	93%	0.101
4	Methylamine	room temperature	2	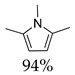 94%	94%	0.128
5	Hexylamine	60	2	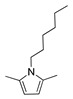 85%	85%	0.086
6	Benzylamine	100	2	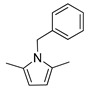 80%	80%	0.088
7	Cysteamine	Room temperature	4	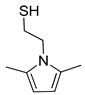 79%	79%	0.076
8	Cystamine	80	2	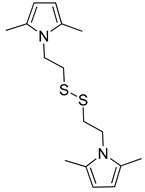 74%	74%	0.080

^a^ 47 mmol of primary amine was added to the solution of 2,5-hexanedione obtained from DF. No purification was needed. ^b^ The carbon efficiency was calculated considering the whole process. ^c^ The 80% yield obtained with serinol was also probably due to the presence of water in the serinol sample used for the reaction. ^d^ The E-factor was calculated considered the total amount of waste produced in both steps.

**Table 3:** E’, E”, and Tan delta values at 10 °C, 23 °C, and 70 °C for composites of Table 1.

		Composite Based on			
Property	Temperature (°C)	Silica	CB/SHP	CB + S	CB
E’ (MPa)	10	14.02	18.42	13.62	12.56
	23	11.00	14.15	9.95	9.27
	70	7.23	9.05	6.18	5.52
E’’ (MPa)	10	7.34	8.67	8.41	7.72
	23	4.11	5.07	4.59	4.27
	70	1.13	1.67	1.32	1.25
Tan Delta	10	0.52	0.47	0.62	0.61
	23	0.37	0.36	0.46	0.46
	70	0.16	0.18	0.21	0.23
ΔE’ (E’_70°C_ − E’_10°C_) (MPa)		6.79	7.44	9.37	7.04

**Table 4:** S-SBR/NR-based composites with silica, CB, CB/SHP adduct as reinforcing fillers ^a^.

	Composite Based on			
Ingredient	Silica	CB/SHP	CB + S	CB
S-SBR 4630	70	70	70	70
NR	30	30	30	30
Silica	65	0	0	0
Silane TESPT	5.2	0	0	0
CB N234	0	0	55	55
CB N234-SHP	0	58.70	0	0
Sulphur S_8_	1.80	1.80	2.57	1.80
Sulphur atoms ^b^	3.04	2.57	2.57	1.80

^a^ Other ingredients used for each composite: ZnO 2.5 phr, stearic acid 2 phr, wax 1 phr, 6PPD 2 phr, TBBS 1.8 phr, PVI 0.5 phr. ^b^ Total sulfur content, including the silane TESPT and SHP.

## Data Availability

Data will be made available on request.

## Supplementary Materials

The following supporting information can be downloaded at: https://www.mdpi.com/article/10.3390/molecules29040861/s1, S1. Reaction mechanism for the functionalization of sp2 carbon allotropes with pyrrole compound; S2. Kinetic studies; S2. Screening of the amount of water; S3. Screening of the amount of acid; S4. Screening of the type of acids, S5. Characterization of bio-pyrroles; S6. Synthesis of SHP and SSP starting from hydrochloride salt; S7. Green metrics calculation for bio-pyrroles [66,67]; S8. Rheometric data.
