# Supplementary material for: Pyrrole Compounds from the Two-Step One-Pot Conversion of 2,5-Dimethylfuran for Elastomer Composites with Low Dissipation of Energy
Source: Molecules. 2024 Feb 15;29(4):861. doi: 10.3390/molecules29040861 (PMC10891845; doi:10.3390/molecules29040861)
Supplement: Supplementary file 1 [file molecules-29-00861-s001.zip › molecules-2851548-supplementary.pdf]

Supplementary Materials

Pyrrole Compounds from the Two Steps One Pot Conversion of 2,5-Dimethylfuran for Elastomer Composites with Low Dissipation of Energy

Simone Naddeo, Davide Gentile, Fatima Margani, Gea Prioglio, Federica Magaletti, Maurizio Galimberti * and Vincenzina Barbera *

Department of Chemistry, Materials and Chemical Engineering "G. Natta", Politecnico di Milano, Via Mancinelli 7, 20131 Milano, Italy; simone.naddeo@polimi.it (S.N.); davide.gentile@polimi.it (D.G.); fatima.margani@polimi.it (F.M.); gea.prioglio@polimi.it (G.P.); federica.magaletti@polimi.it (F.M.)

* Correspondence: maurizio.galimberti@polimi.it (M.G.); vincenzina.barbera@polimi.it (V.B.)

Table of Contents

S1. Reaction mechanism for the functionalization of sp2 carbon allotropes with pyrrole compound 3

S2. Kinetic studies 4

S2. Screening of the amount of water 6

S3. Screening of the amount of acid 10

S4. Screening of the type of acids 12

S5. Characterization of bio-pyrroles 15

S6. Synthesis of SHP and SSP starting from hydrochloride salt 39

S7. Green metrics calculation for bio-pyrroles 43

S8. Rheometric data……………………………………………………………………………44

S.1. Reaction mechanism for the functionalization of sp^2 carbon allotropes with pyrrole compounds

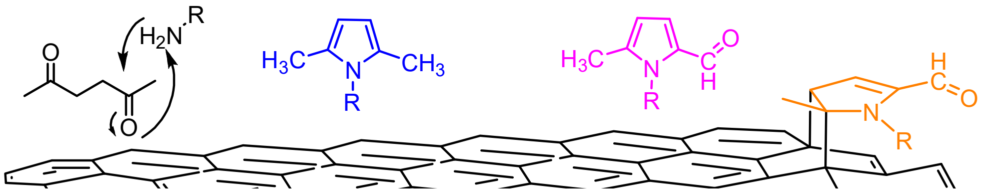

Figure S.1. Reaction mechanism for the functionalization of sp^2 carbon allotropes with a pyrrole compound with methyl groups in α positions. It is also shown the synthesis of the pyrrole compound.

S.2. Kinetic studies for the ring opening reaction of DF to HD

The kinetics of the ring opening reaction of DF to HD was studied collecting 5 aliquots of the reaction mixture at increasing times and analysing them by means of ^{1}H-NMR spectroscopies.

Reaction parameters: molar ration DF / water = 1 / 1, 15 mol% of H_2SO_4, 50 °C. ^{1}H-NMR spectra were collected in $CDCl_3$ after 2 hours, 3 hours and 30 minutes, 5 hours, 8 hours and 24 hours. They are shown in Figure S.2 a-e, respectively. The red arrows show the methyl group of DF. It is evident the decreasing of the concentration of DF as the reaction time increases.

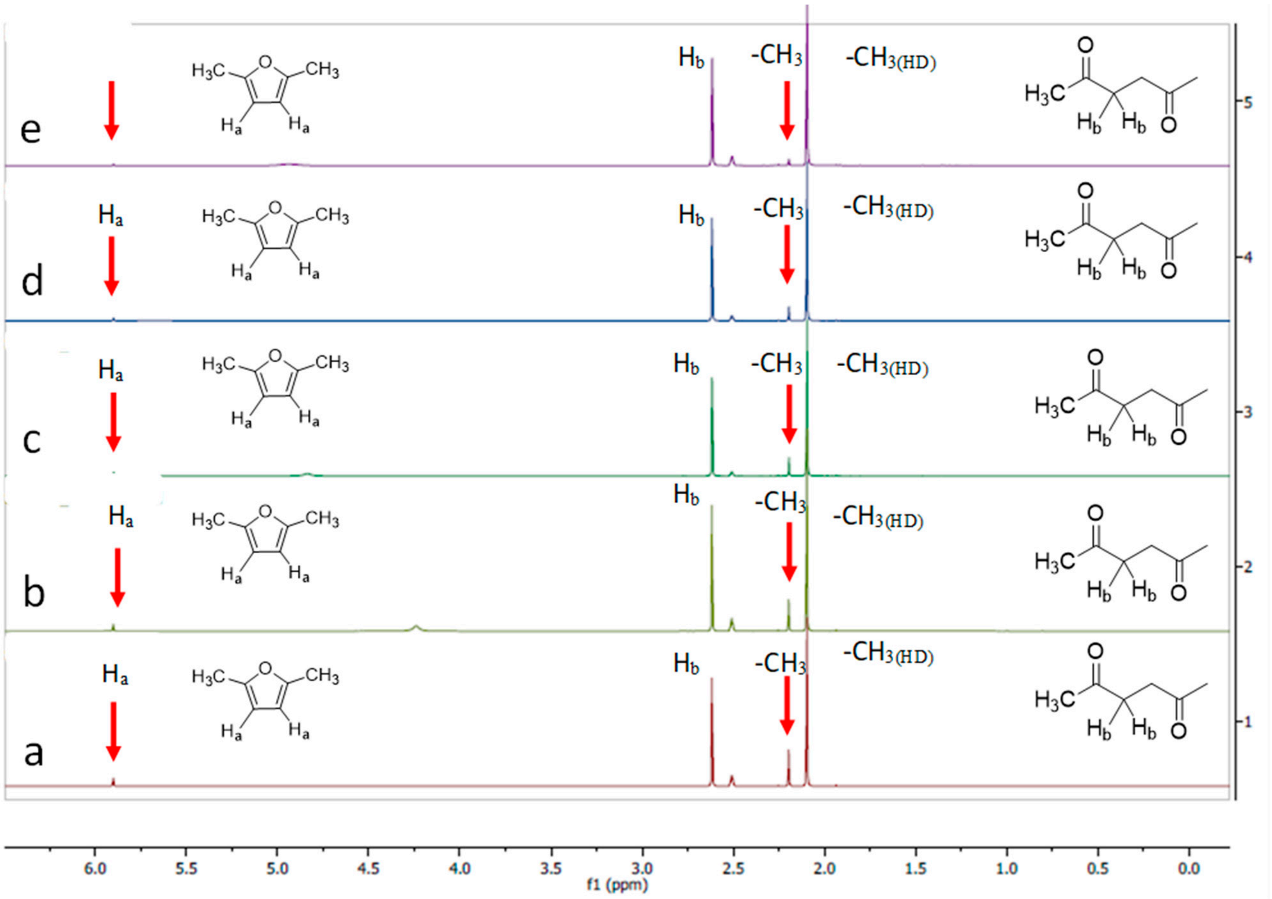

Figure S.2. ^{1}H NMR spectra (400 MHz, DMSO-d_6) recorded on the ring opening reaction of 2,5-dimethylfuran to 2,5-hexanedione.

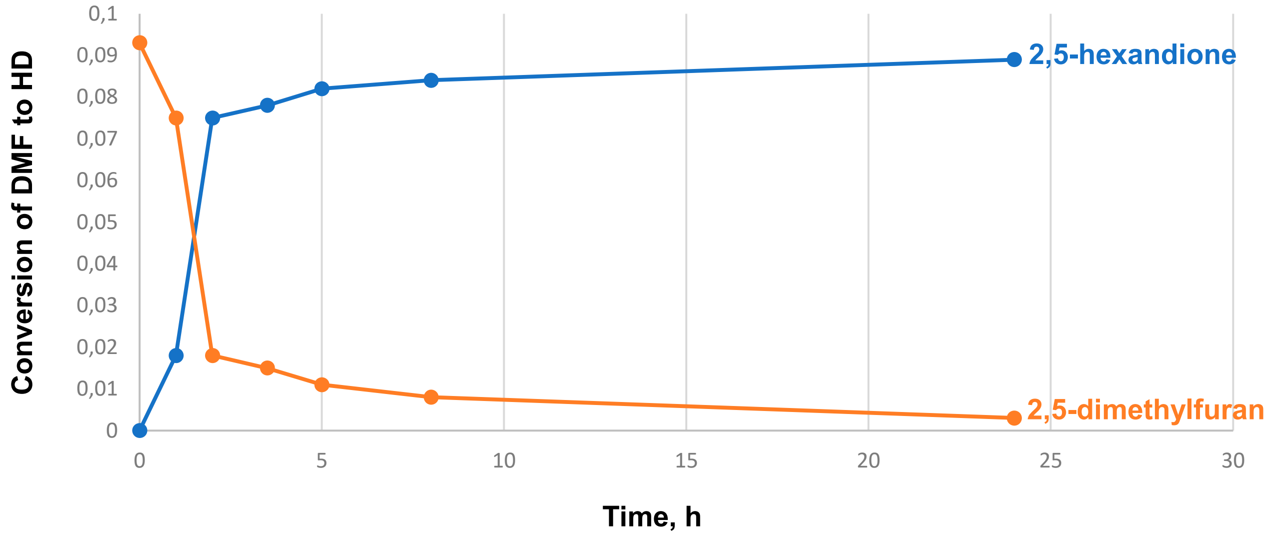

Figure S.3. Conversion of the ring opening reaction of DF to HD, at different reaction times. The graph shows the relative concentration of DF (orange line) and HD (blue line) vs time.

S2. Screening of the amount of water

Different DF/H_2O ratio were investigated. The reactions were followed by NMR spectroscopy.

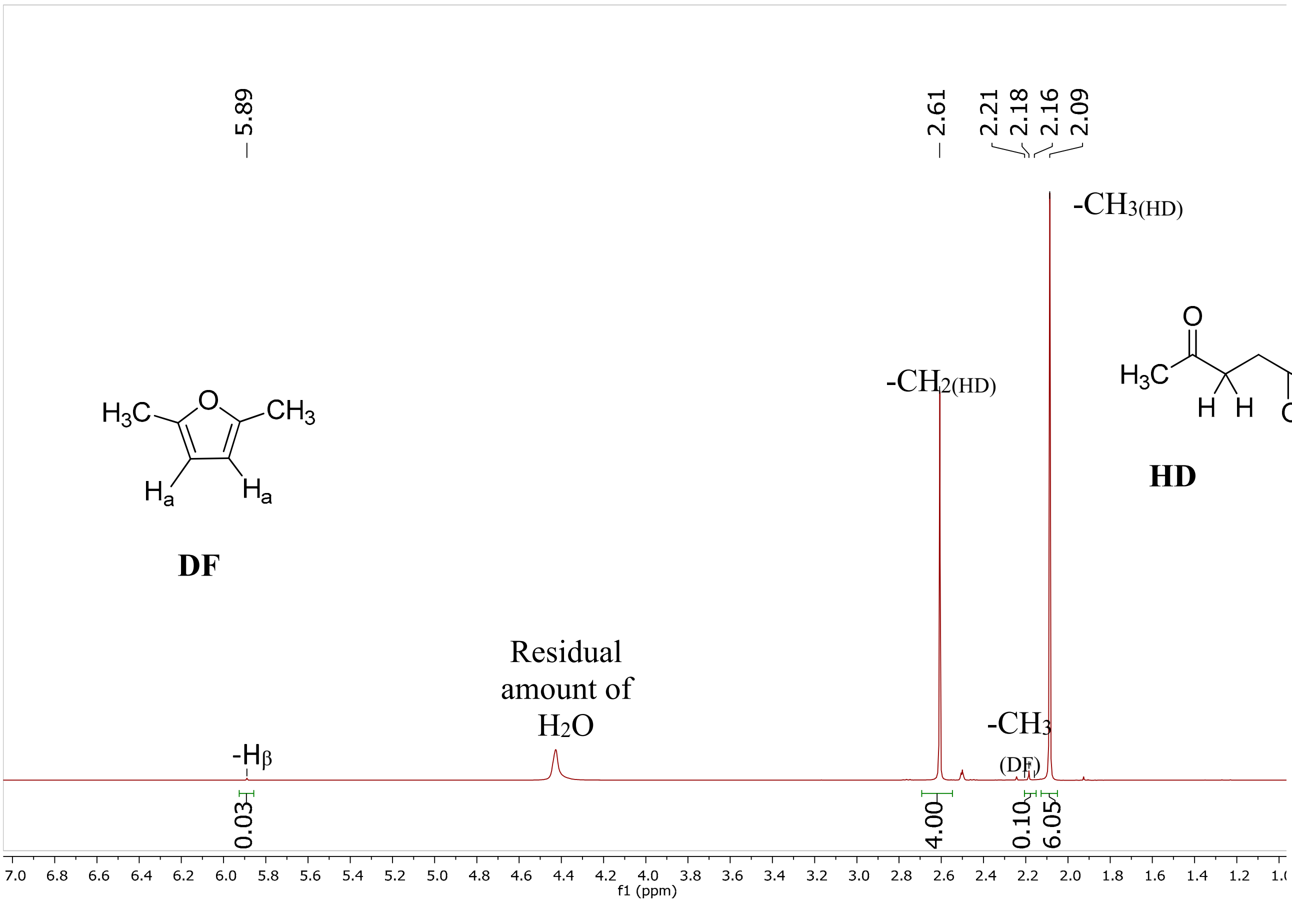

Figure S.4. ^{1}H-NMR spectrum (400 MHz, DMSO-d_6) of the reaction between DF, water (ratio DF/H_2O 1:3.0) and sulfuric acid 15 mol%. DF was converted by 95% in HD; residual amount of water was observed at 4.39 ppm.

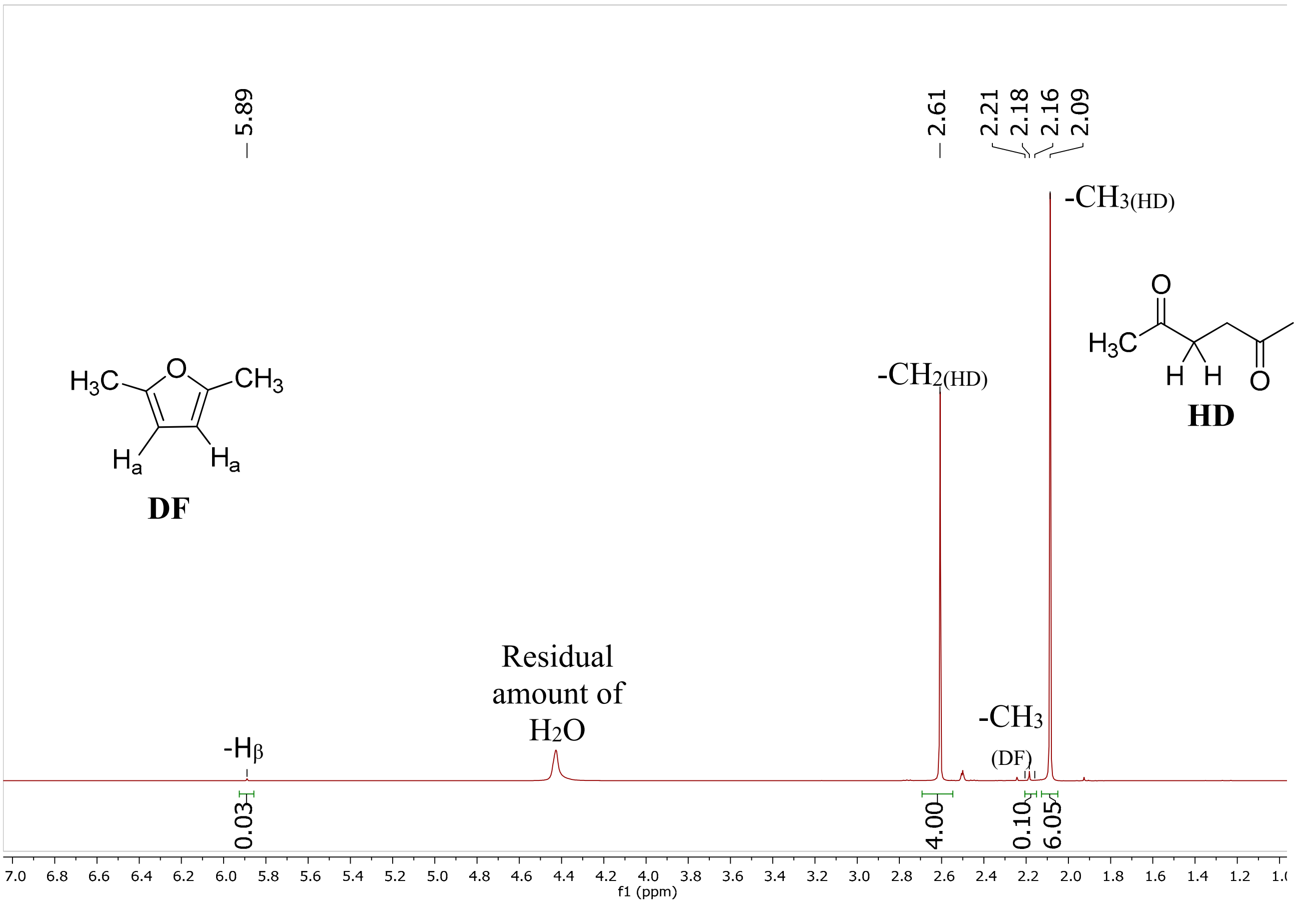

Figure S.5. ^{1}H-NMR spectrum (400 MHz, DMSO-*d*$_6$) of the reaction between DF, water (ratio DF/H_2O 1/2.0) and sulfuric acid 15 mol%. DF was converted by 95% in HD; residual amount of water was observed at 4.43 ppm.

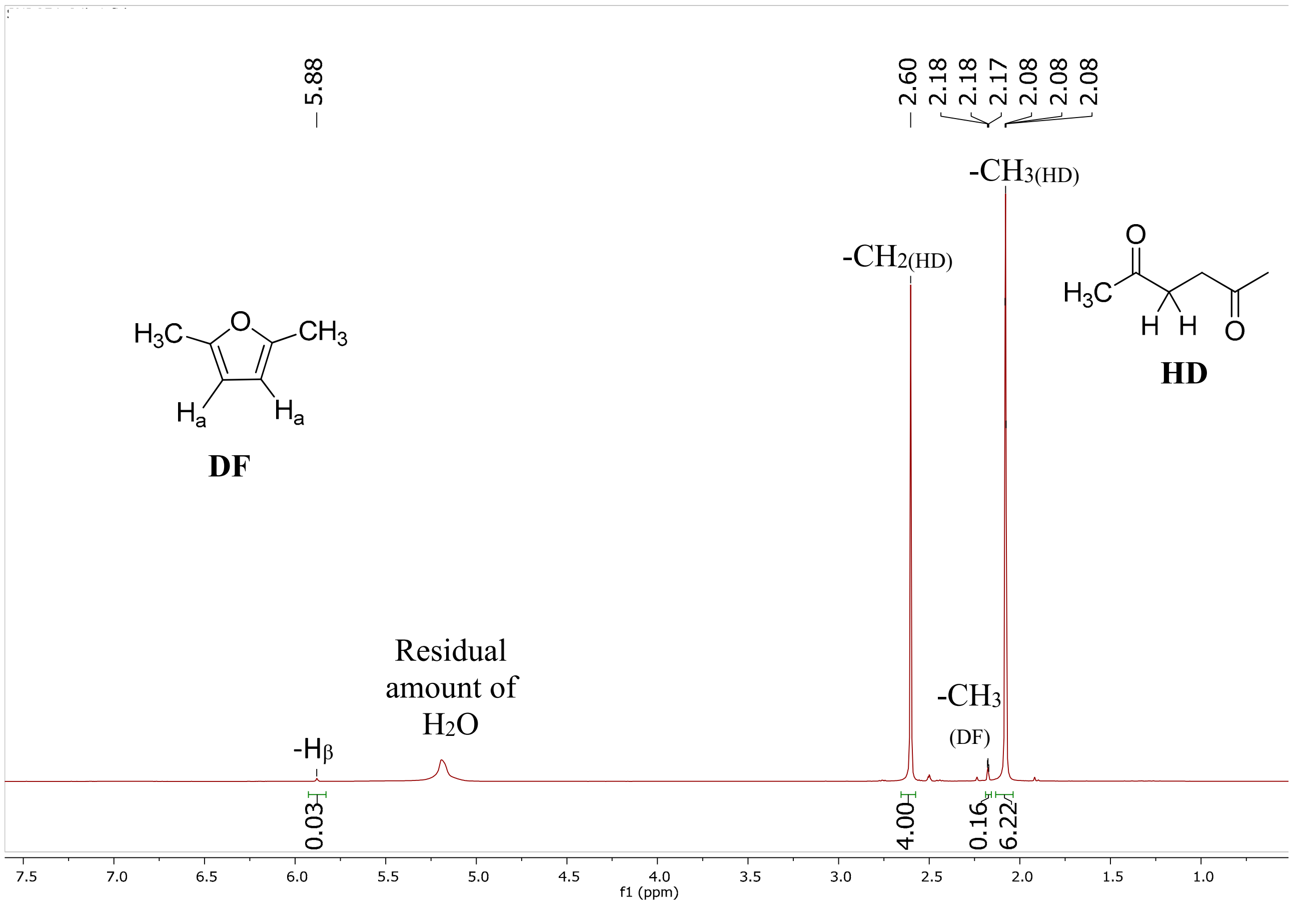

Figure S.6. ^{1}H-NMR spectrum (400 MHz, DMSO-*d*$_6$) of the reaction between DF, water (ratio DF/H_2O 1: 1.5) and sulfuric acid 15 mol%. DF was converted by 95% in HD; residual amount of water was observed at 5.19 ppm

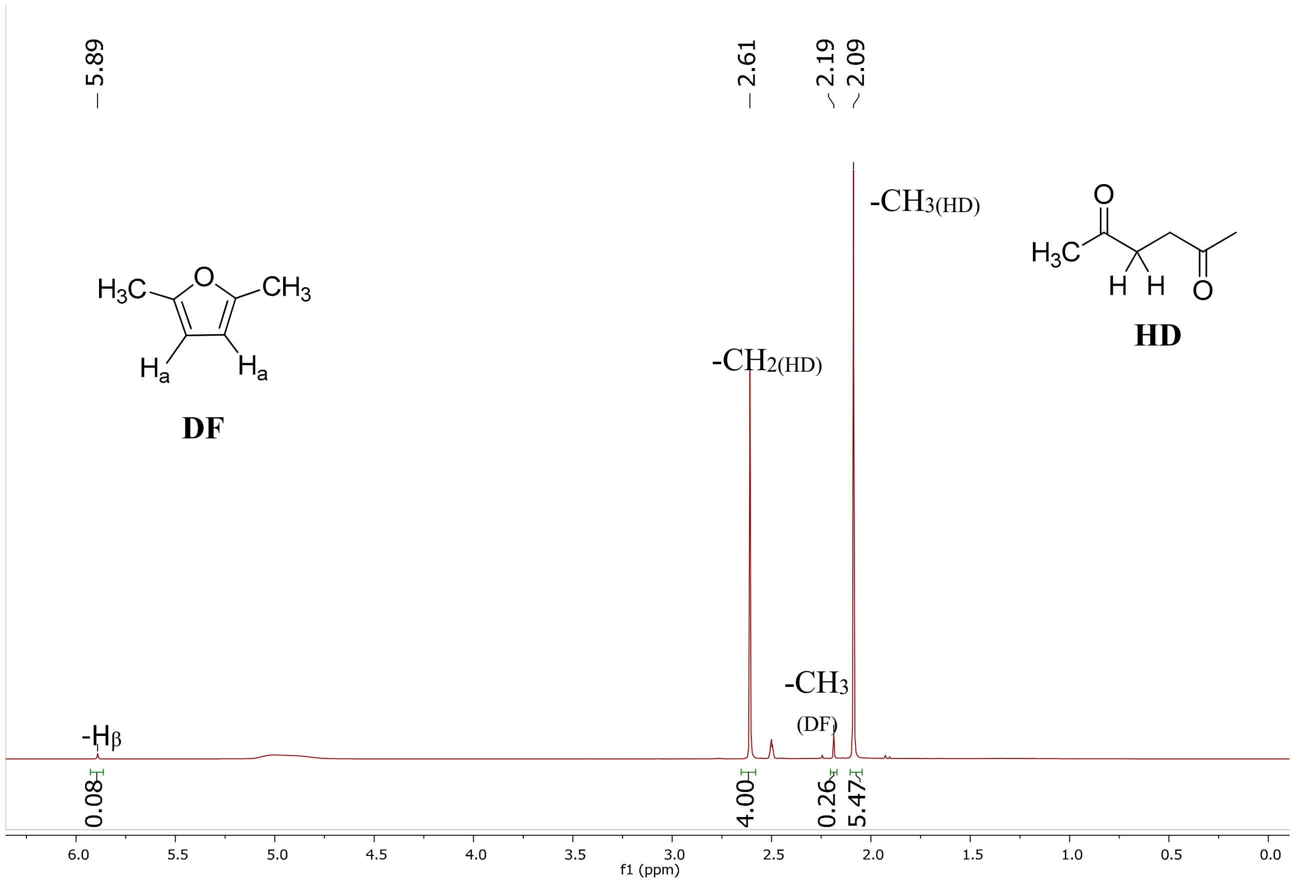

Figure S.7. ^{1}H-NMR spectrum (400 MHz, DMSO-*d6*) of the reaction between DF, water (ratio DF / H_2O 1: 1) and sulfuric acid 15 mol%. DF was converted by 95% in HD

S3. Screening of the amount of acid

The amount of sulfuric acid was investigated. The reactions were followed by NMR spectroscopy.

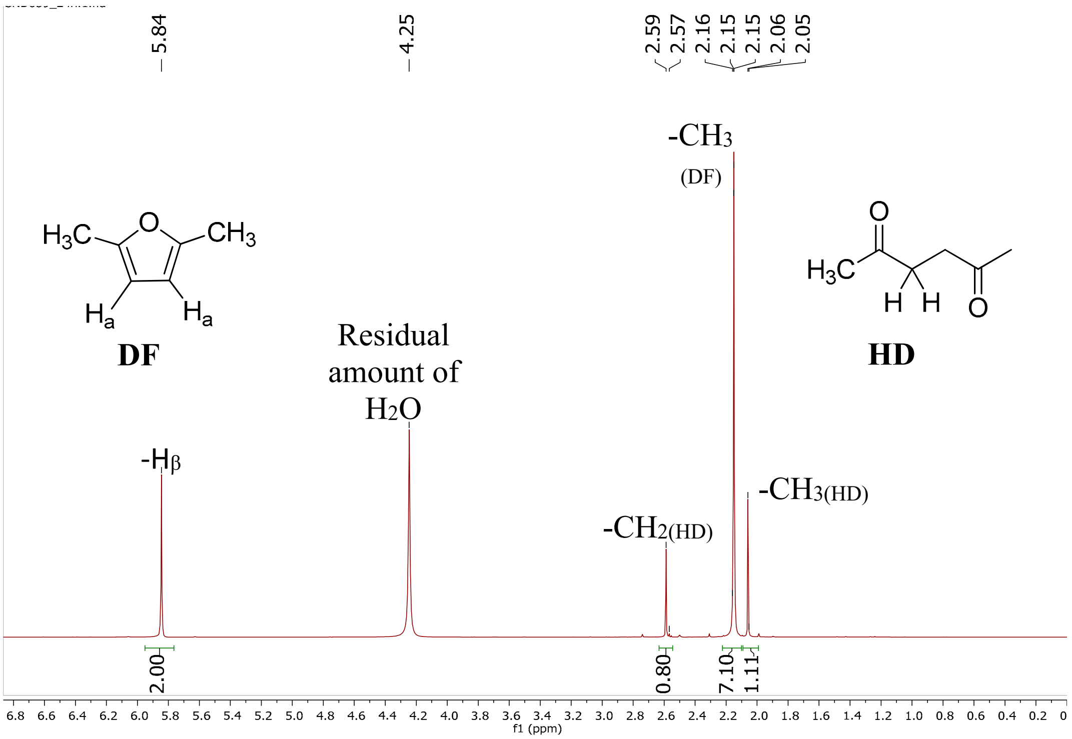

Figure S.8. ^{1}H NMR spectrum (400 MHz, DMSO-*d6*) of the reaction DF, water (ratio DF/H_2O 1: 1) and 1.7 mol% of sulfuric acid. DF was converted by only 17% in HD.

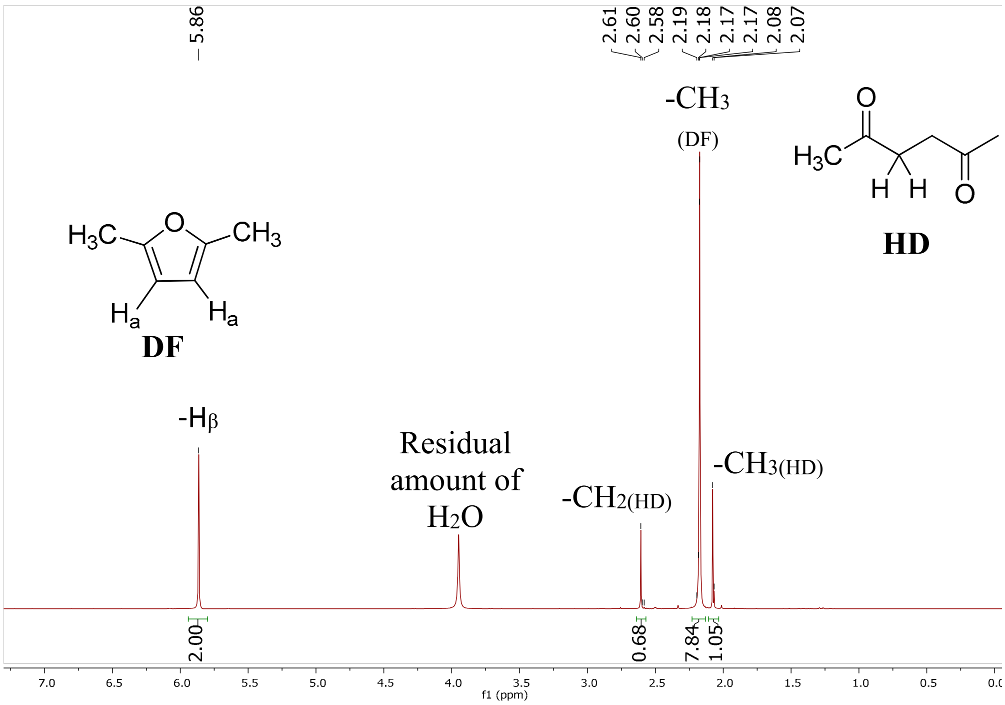

Figure S.9. ^{1}H-NMR spectrum (400 MHz, DMSO-*d6*) of the reaction between DF, water (molar ratio DF/H_2O 1: 1) and catalitic amount of sulfuric acid. DF was converted by only 13% in HD.

S4. Screening of the type of acids

Different acids were investigated. The reactions were followed by NMR spectroscopy.

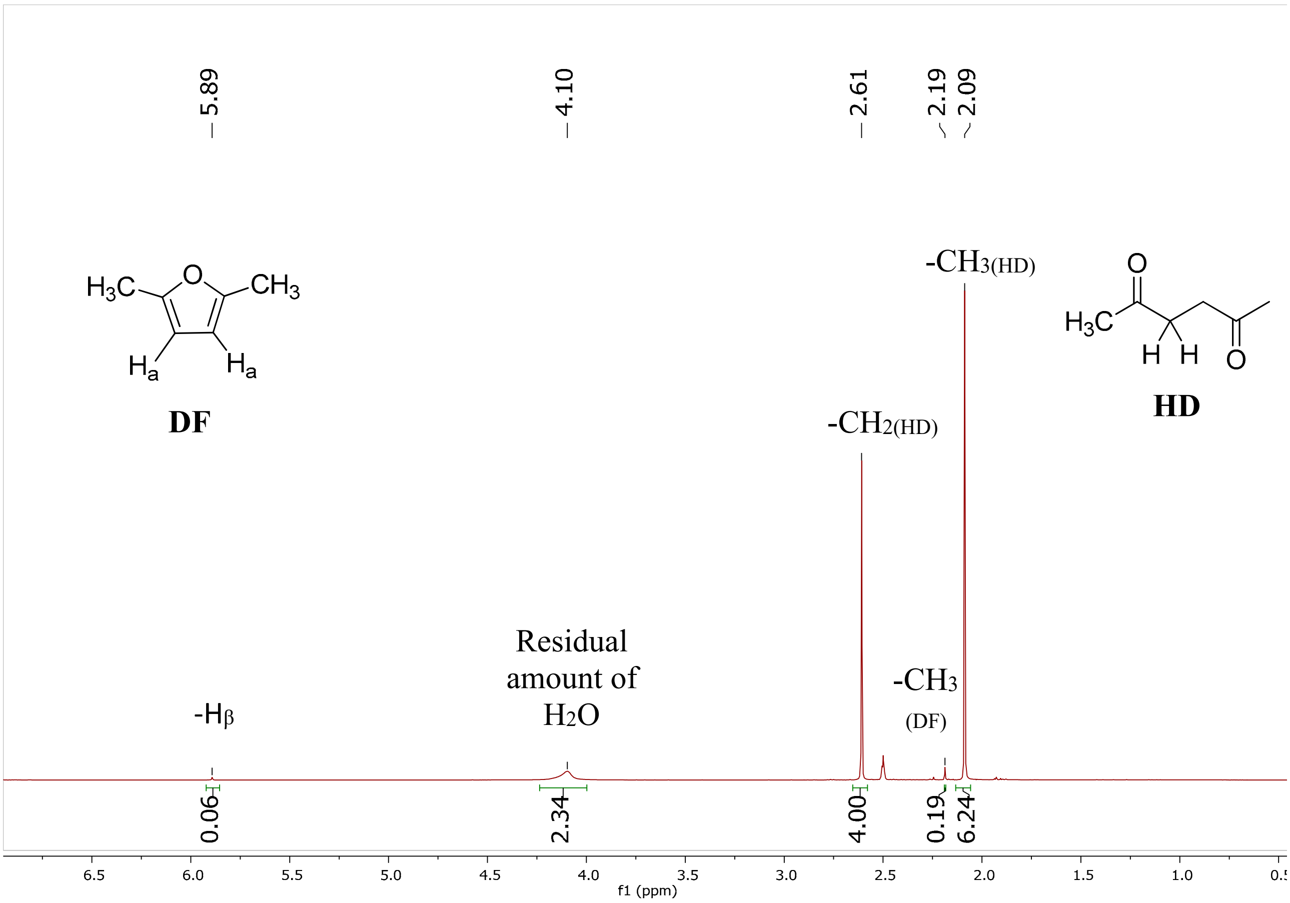

Figure S.10. ^{1}H-NMR spectrum (400 MHz, DMSO-d_6) of the reaction between DF, water and 4 mol% of hydrochloridric acid. Residual water was observed at 4.10 ppm.

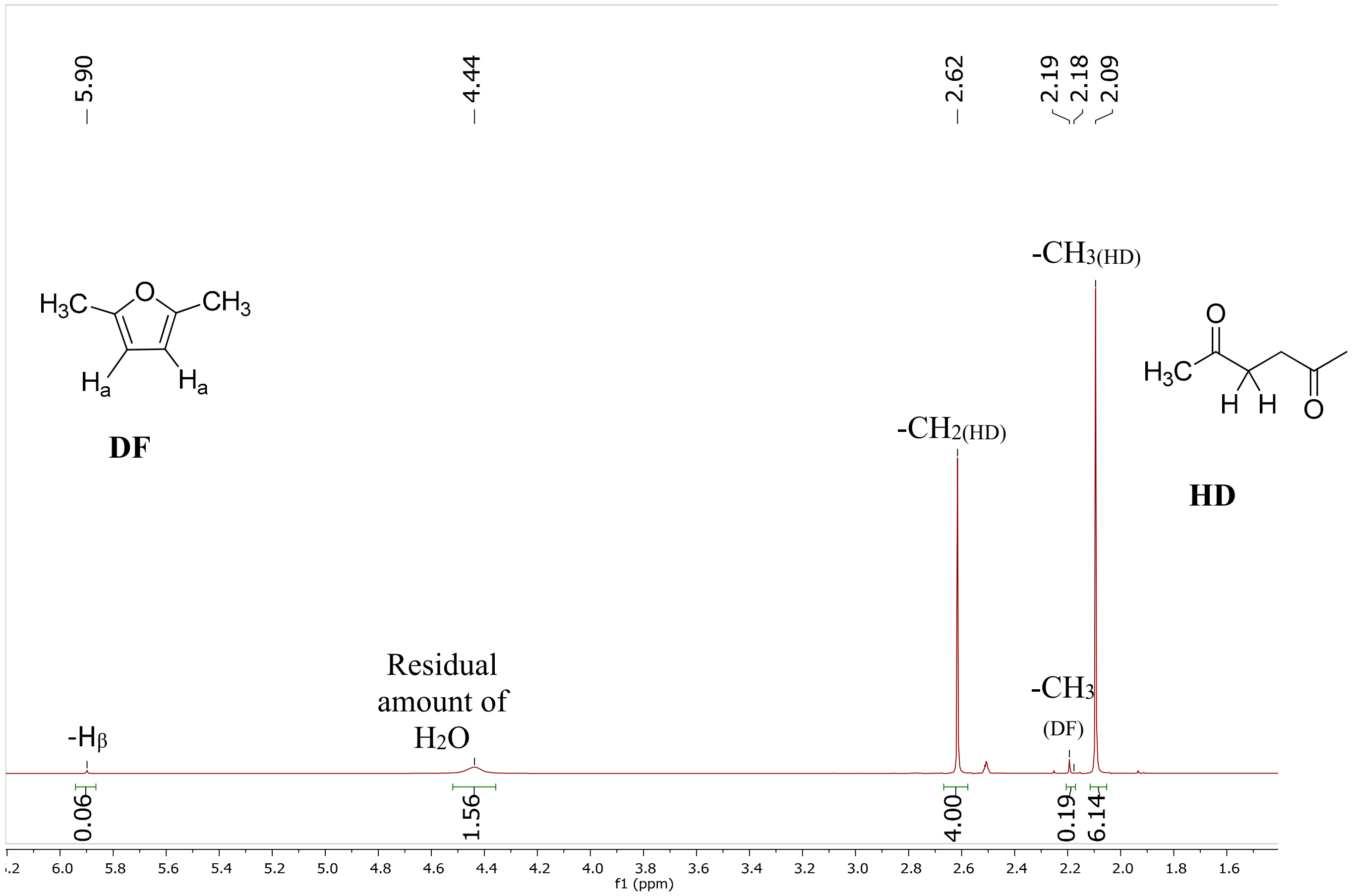

Figure S.11. ^{1}H NMR spectrum (400 MHz, DMSO-*d*6) of the reaction between DF, water and 4 mol% of hydrobromidric acid. Residual water was observed at 4.44 ppm.

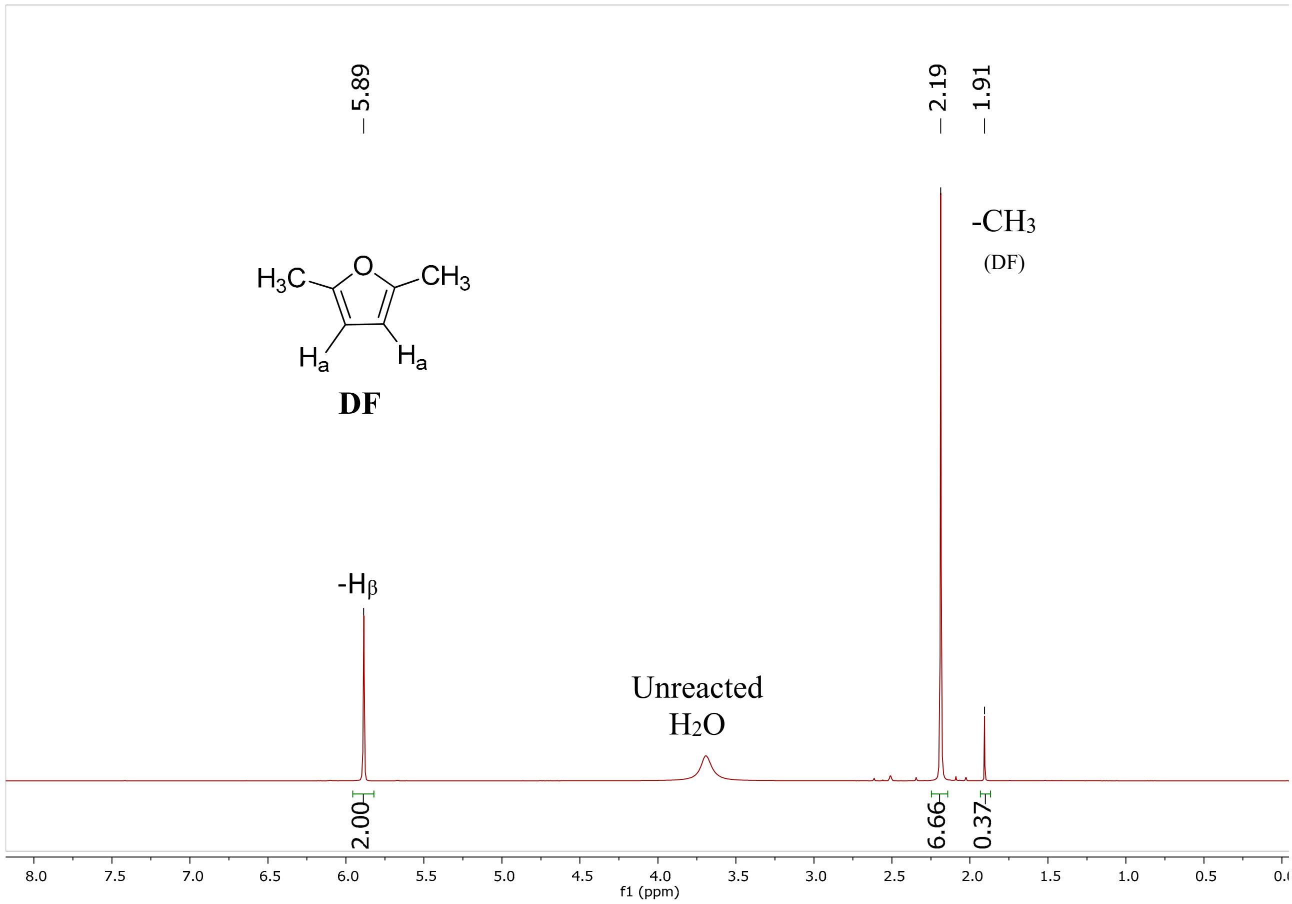

Figure S.12. ^{1}H NMR spectrum (400 MHz, DMSO-*d*$_6$) of the reaction between DF, water and 4 mol% of acetic acid. DF was not converted in HD.

S5. Characterization of bio-pyrroles

All pyrrole derivatives were characterized by NMR spectroscopy and further confirmed by Gas Chromatography Mass (GC-Mass) and Electrospray Ionization Mass (ESI-MS) spectrometries.

3a) ***2-(2,5-dimethyl-1H-pyrrol-1-yl)propane-1,3-diol*** (Serinol pyrrole, SP)

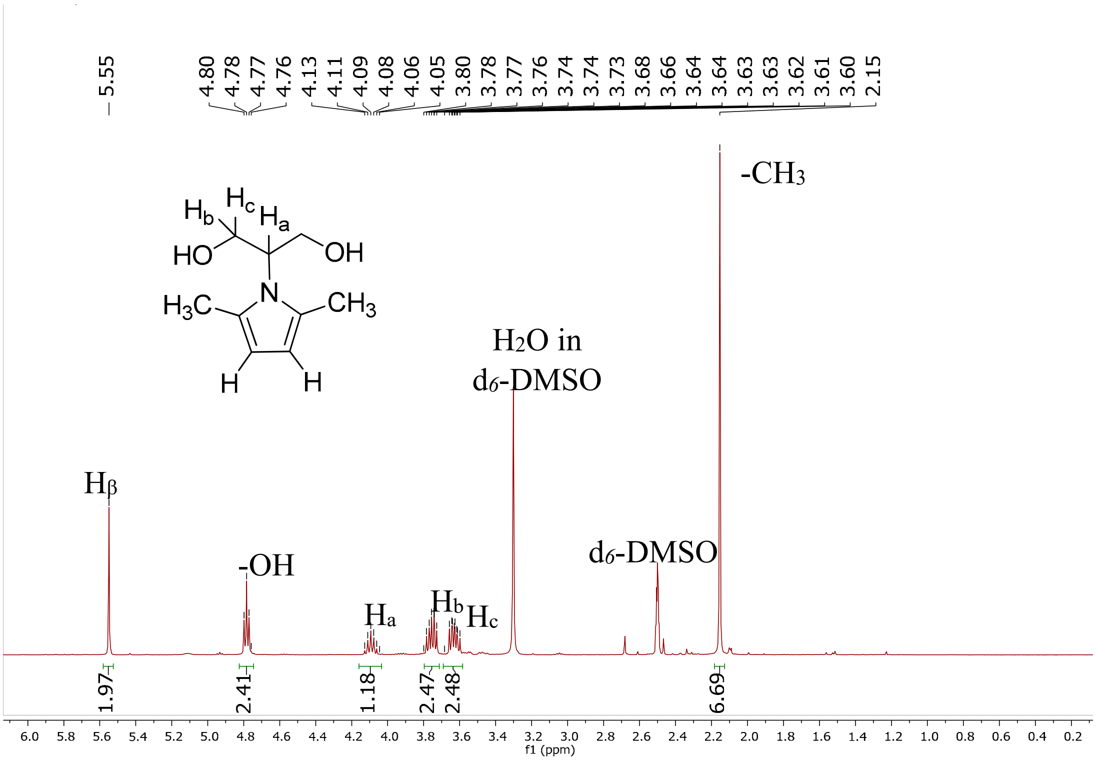

(A)

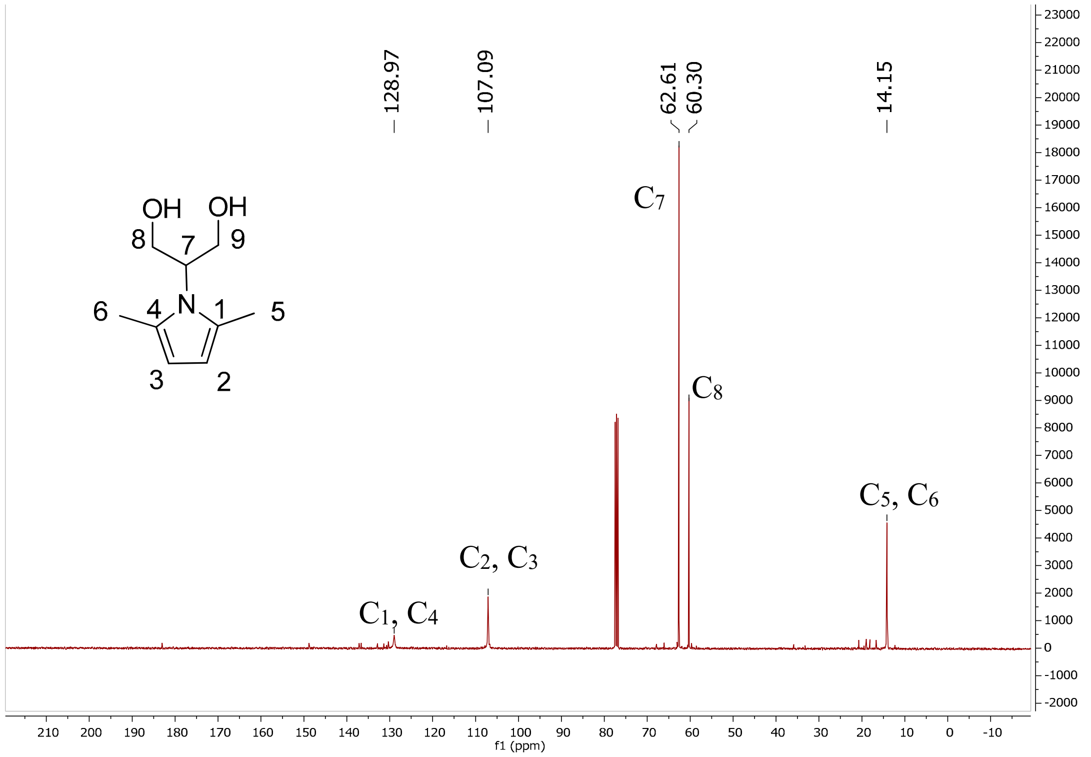

(B)

Figure S.13 NMR spectra of (A) ^{1}H (400 MHz, DMSO-*d6*) and (B) ^{13}C of 2-(2,5-dimethyl-1*H*-pyrrol-1-yl)propane-1,3-diol (Serinol pyrrole, SP).

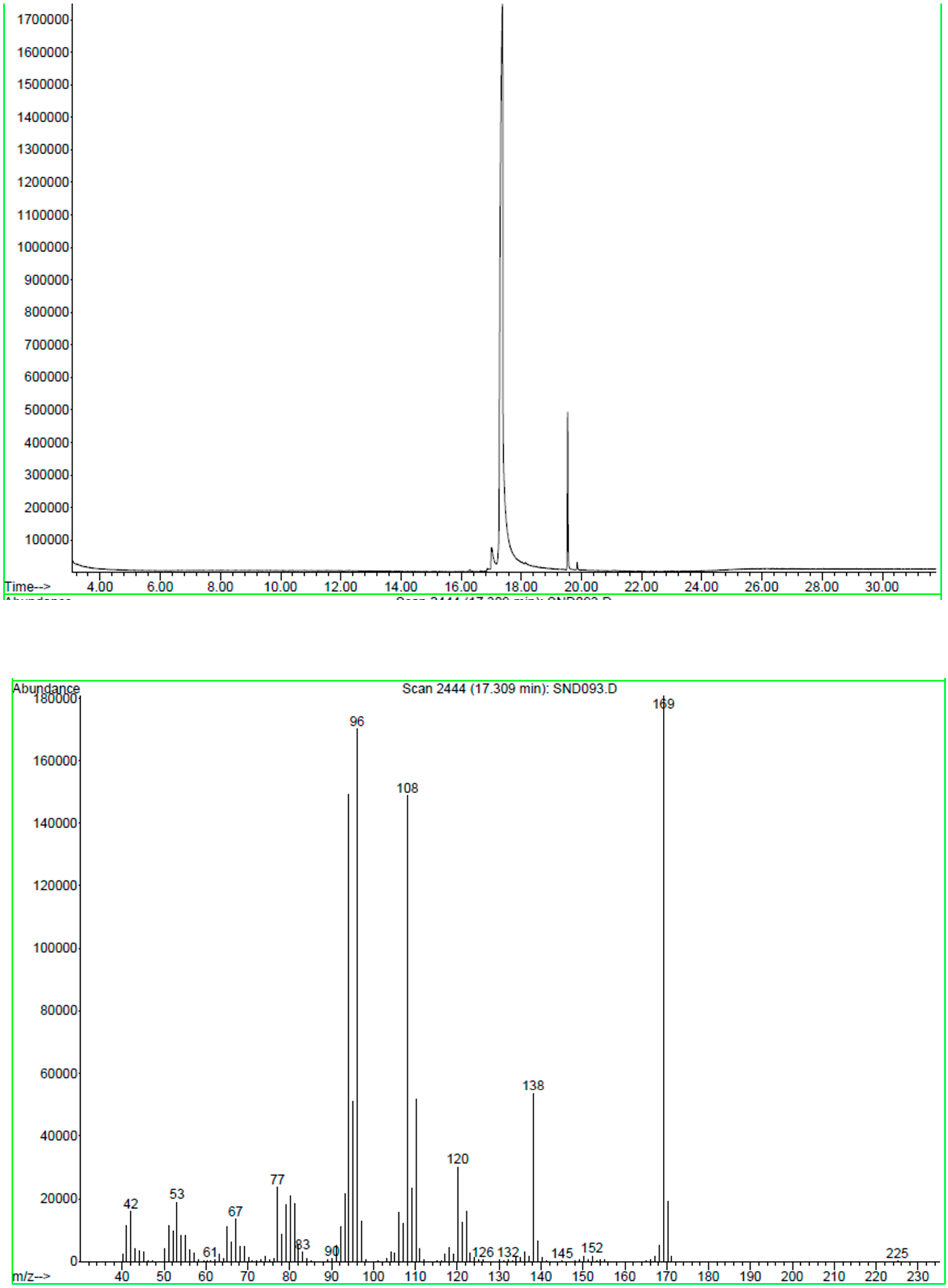

Figure S.14: GC-Mass chromatogram of Serinol pyrrole.

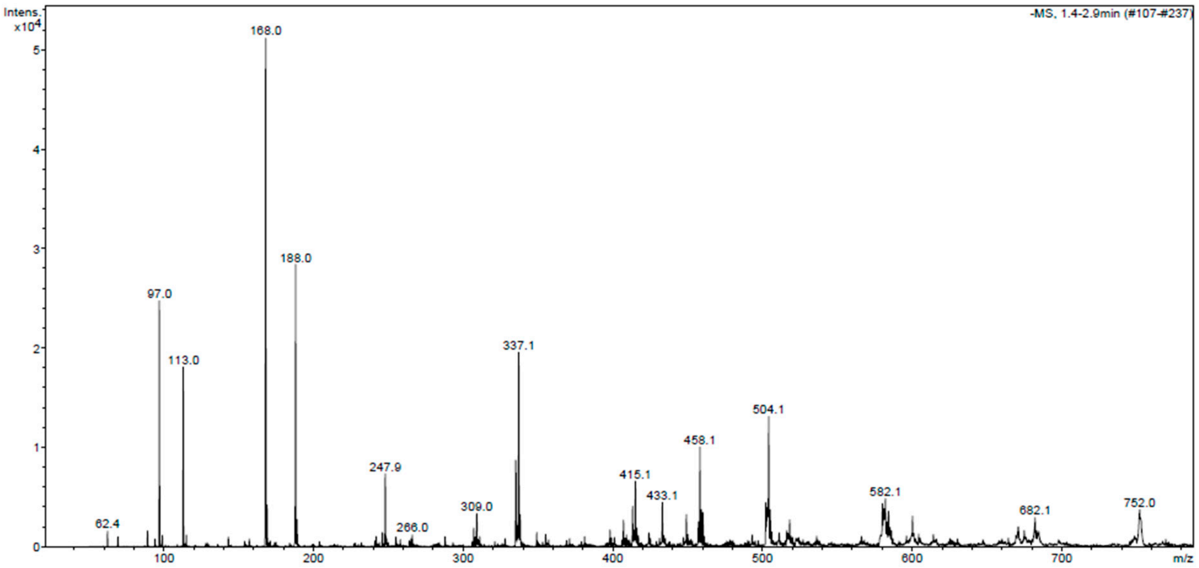

Figure S.15. Positive ESI-MS spectrum (1:100 MeOH) of Serinol pyrrole

3b) ***3-(2,5-dimethyl-1H-pyrrol-1-yl)propane-1,2-diol*** (iso-SP)

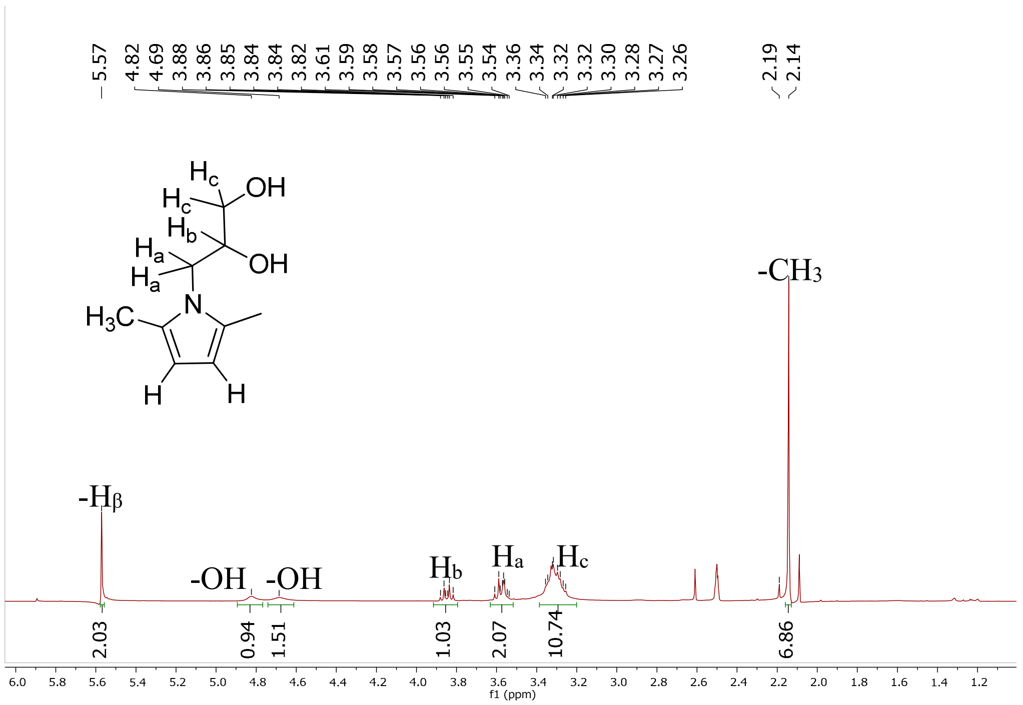

(A)

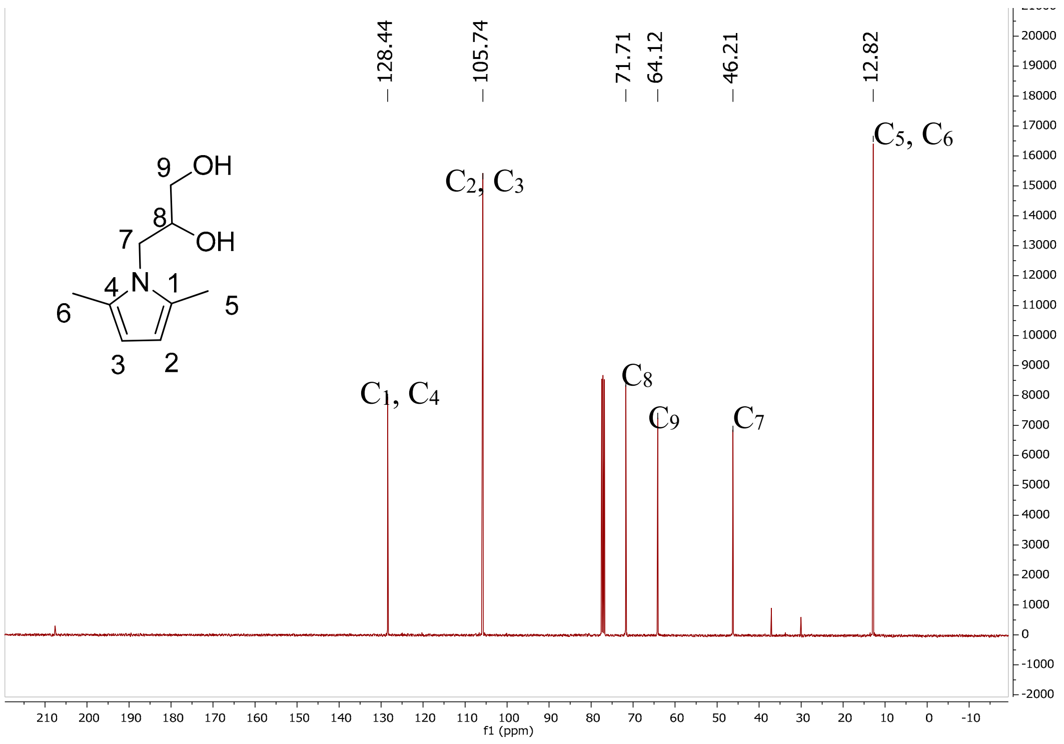

(B)

Figure S.16. NMR spectra of (A) ^{1}H (400 MHz, DMSO-d_6) and (B) ^{13}C of 3-(2,5-dimethyl-1*H*-pyrrol-1-yl)propane-1,2-diol (iso-Serinol Pyrrole).

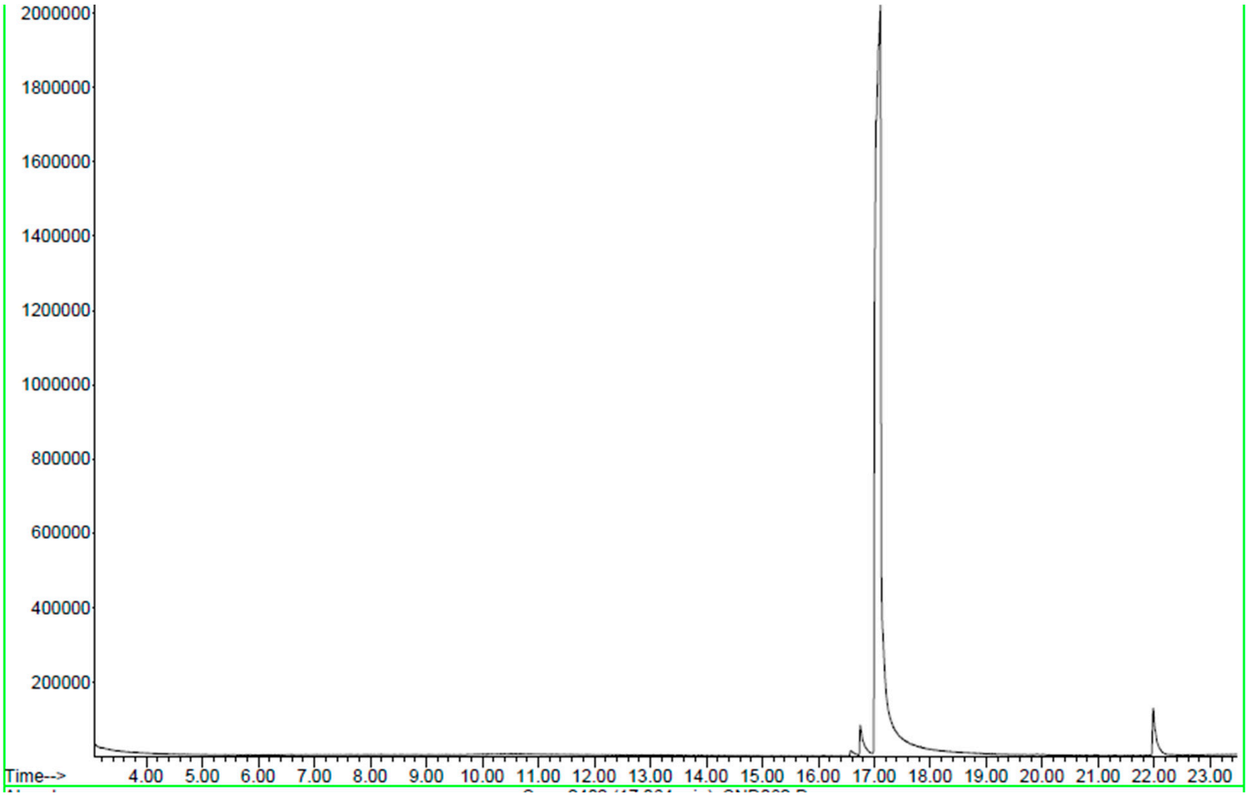

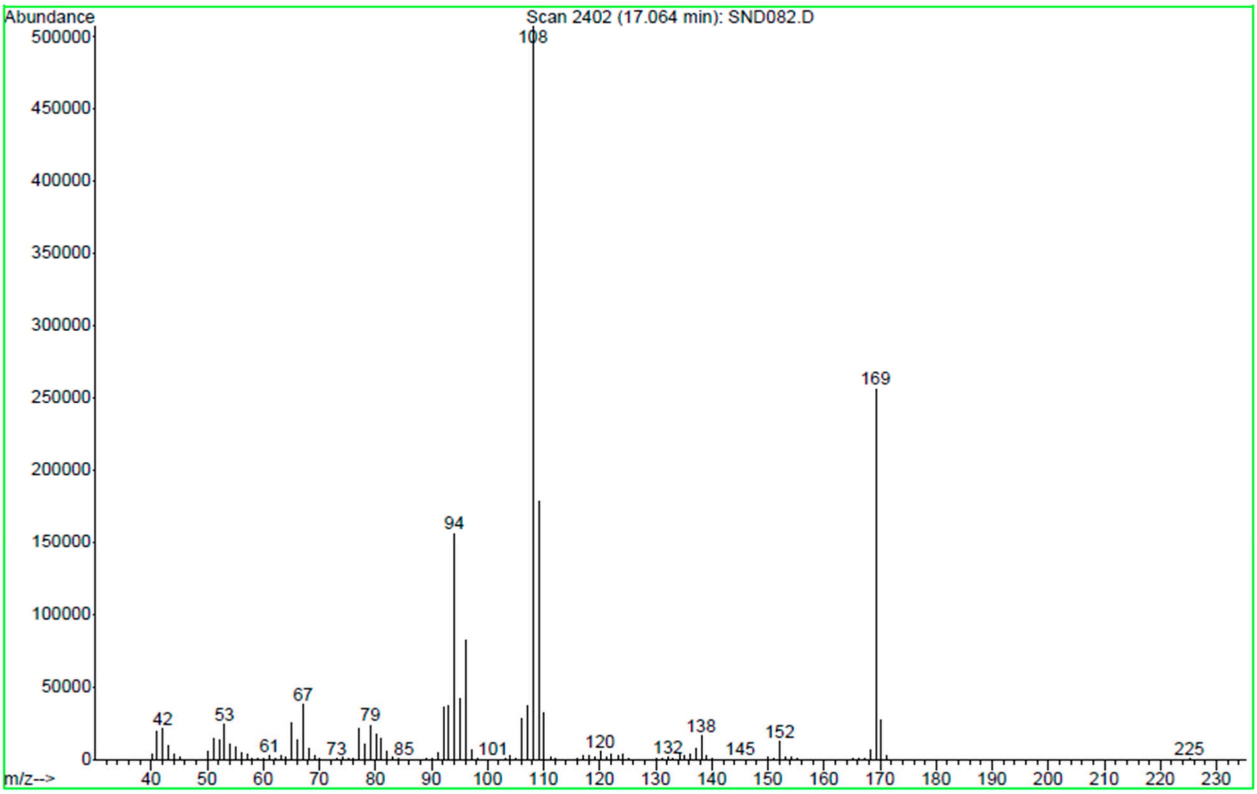

Figure S.17: GC-Mass chromatogram of 3-(2,5-dimethyl-1*H*-pyrrol-1-yl)propane-1,2-diol

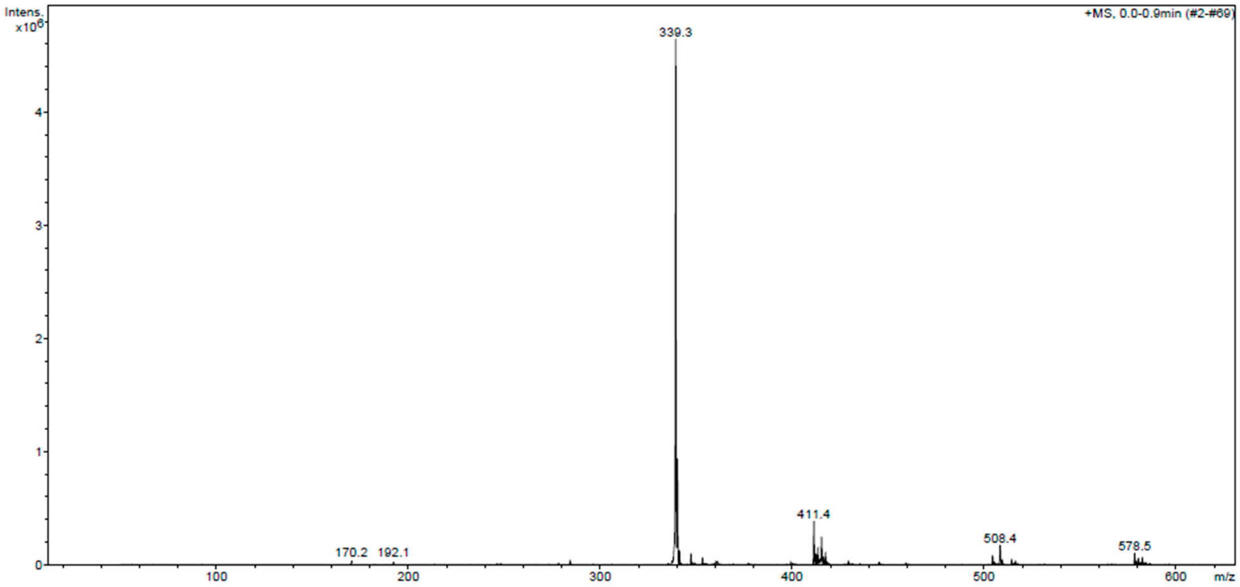

Figure S.18. Positive ESI-MS spectrum (1:100 MeOH) of 3-(2,5-dimethyl-1*H*-pyrrol-1-yl)propane-1,2-diol

3c) 2-*(2,5-dimethyl-1H-pyrrol-1-yl)ethanol* (ethanol pyrrole)

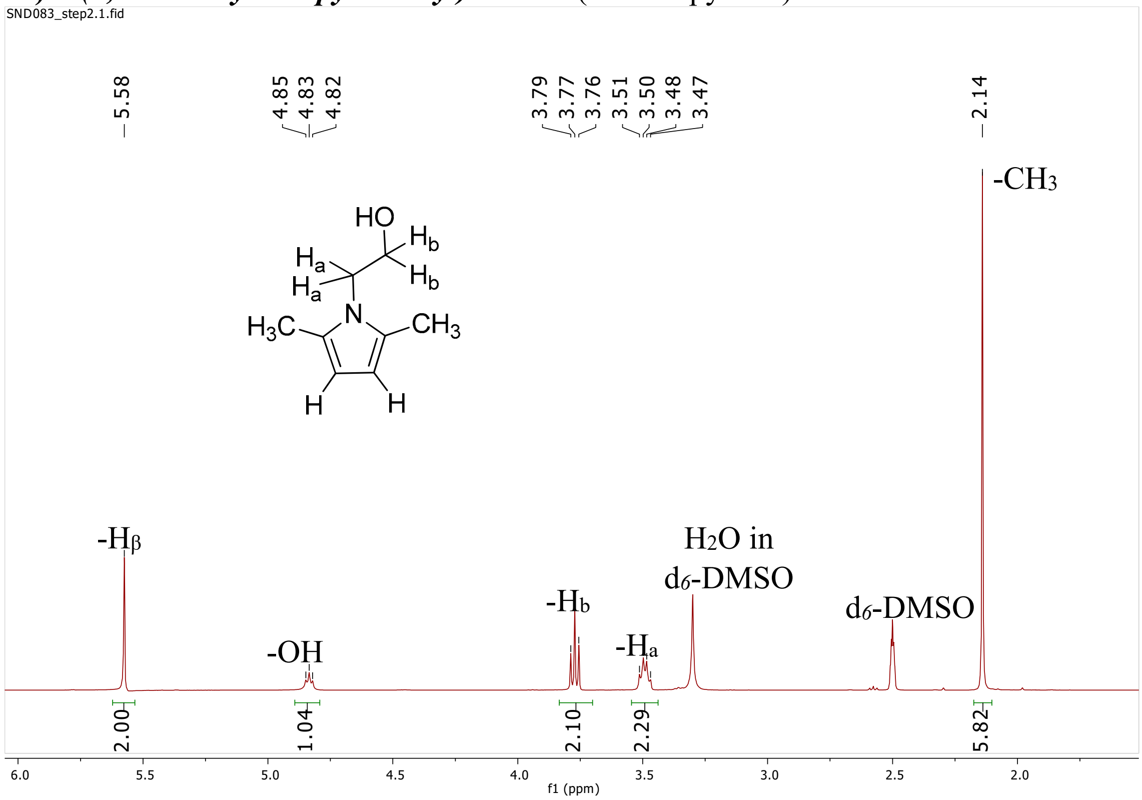

(A)

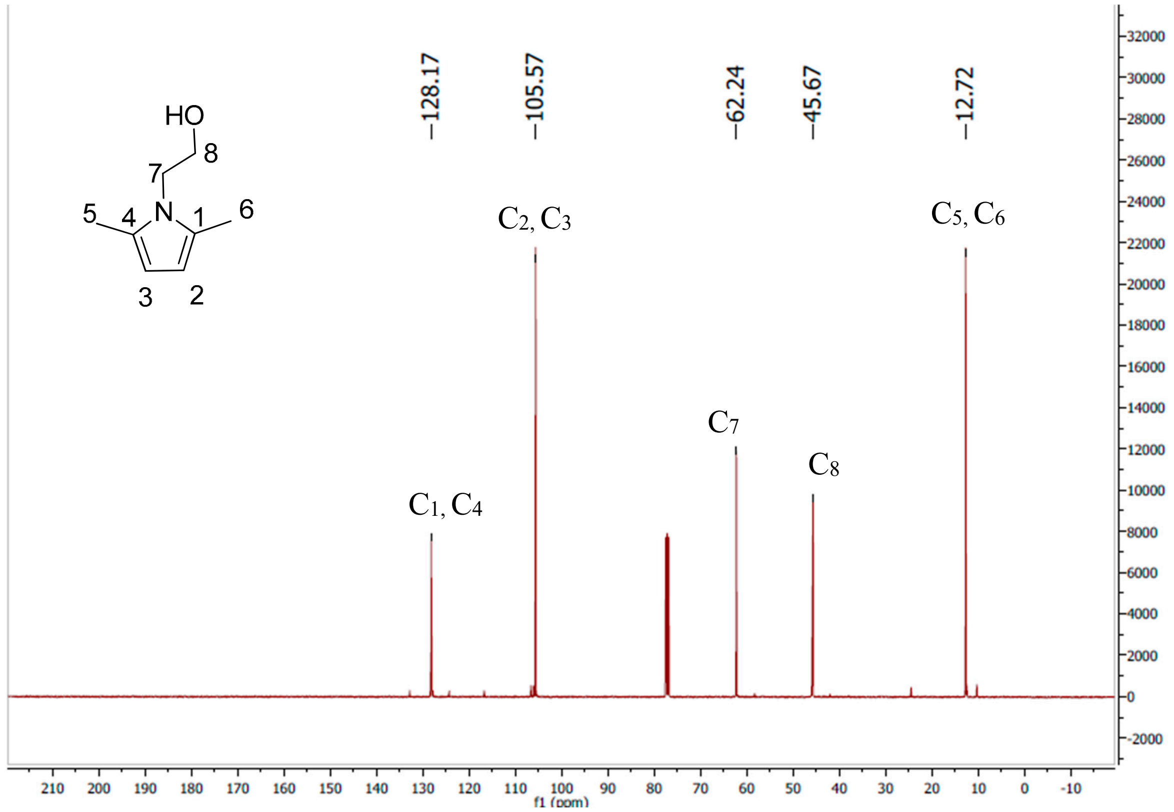

(B)

Figure S.19. NMR spectra of (A) ^{1}H (400 MHz, DMSO-*d6*) and (B) ^{13}C of 2-(2,5-dimethyl-1*H*-pyrrol-1-yl)ethanol (ethanol pyrrole)

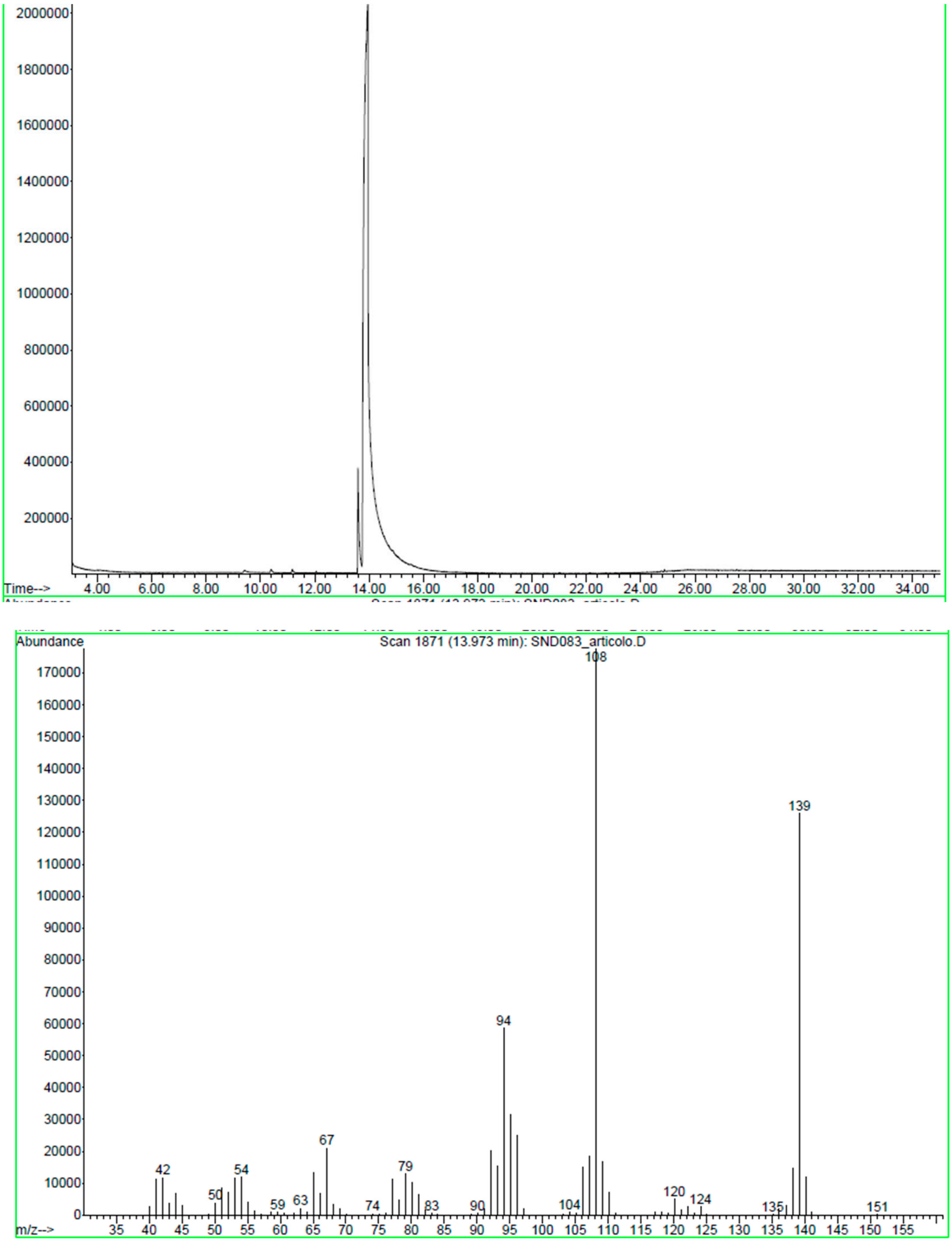

Figure S.20. GC-Mass chromatogram of -(2,5-dimethyl-1*H*-pyrrol-1-yl)ethanol (ethanol pyrrole)

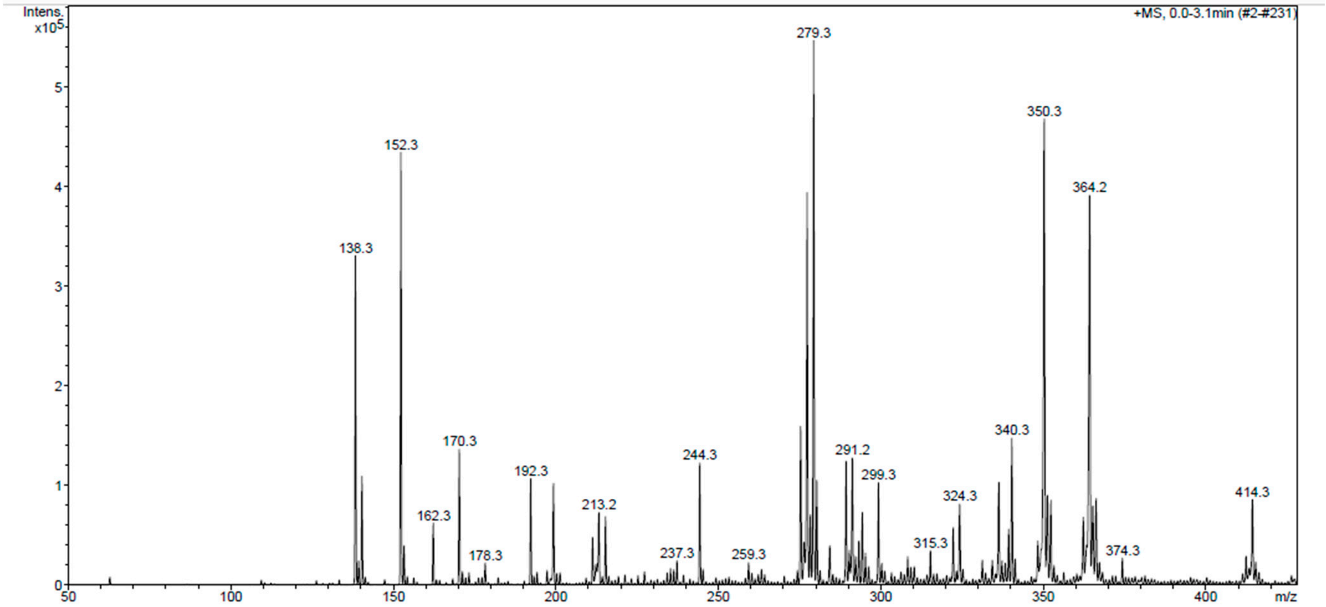

Figure S.21. Positive ESI-MS spectrum (1:100 MeOH) of ethanol pyrrole.

3d) 1,2,5-*trimethyl-1H-pyrrole* (trimethyl pyrrole)

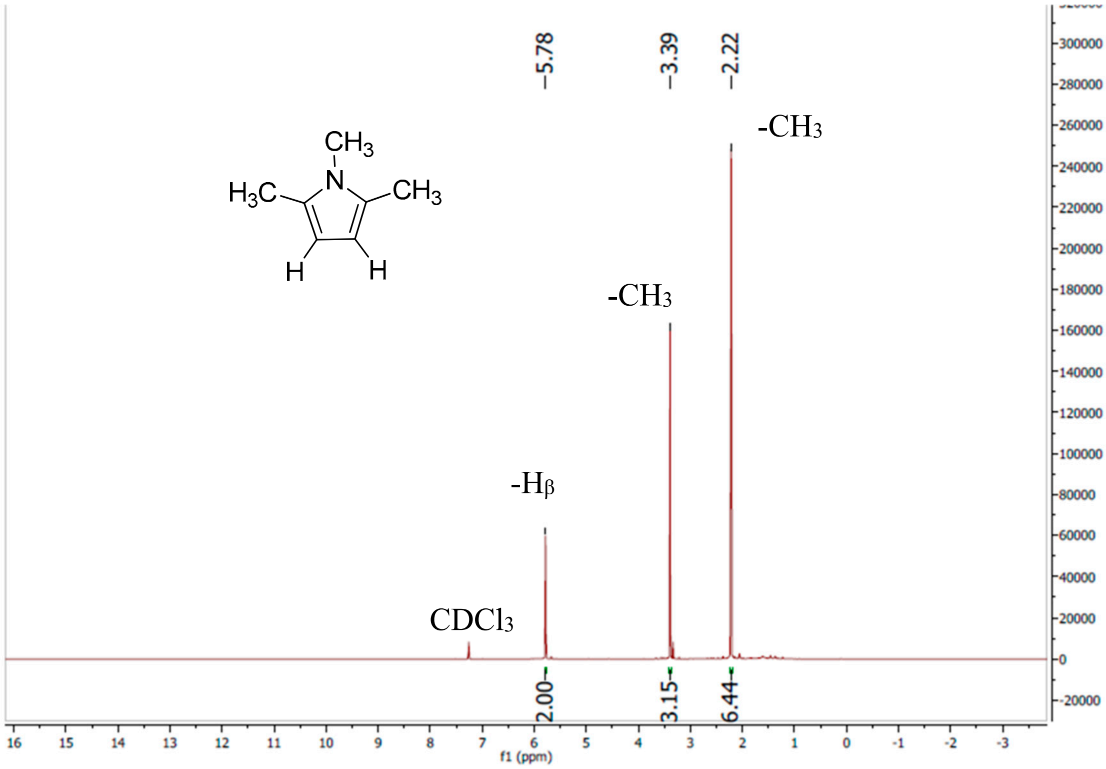

(A)

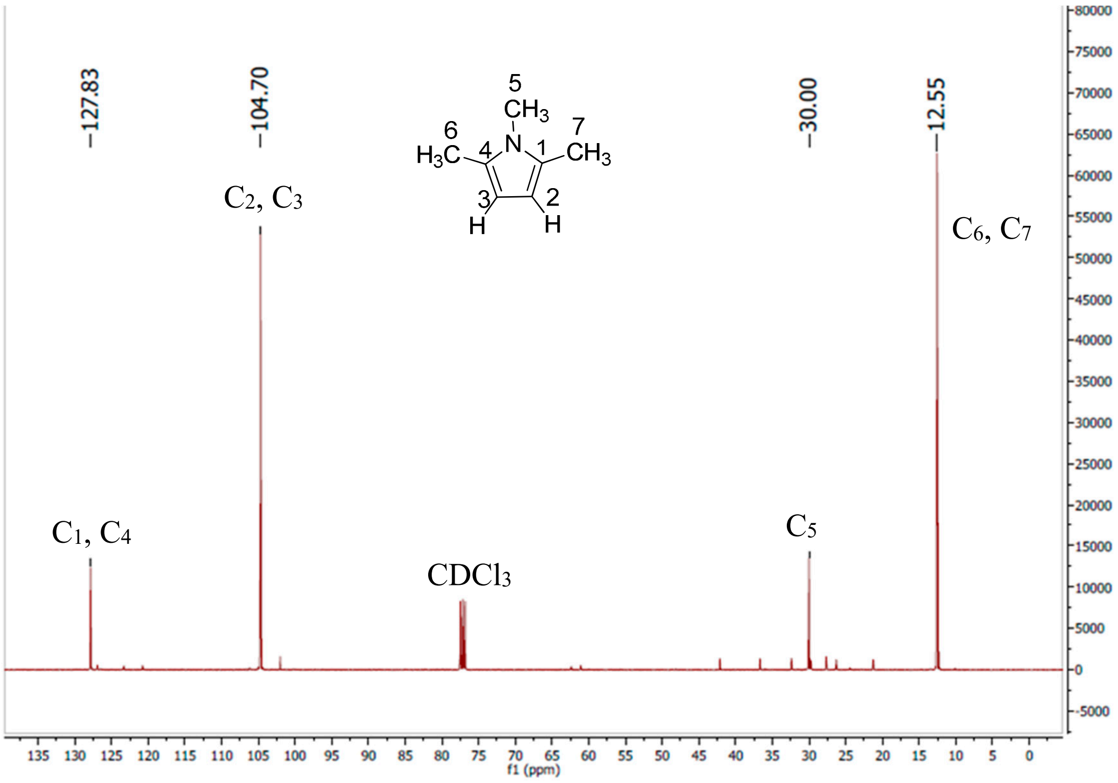

(B)

Figure S.22. NMR spectra of (A) ^{1}H (400 MHz, $CDCl_3$) and (B) ^{13}C of 1,2,5-trimethyl-1*H*-pyrrole (trimethyl pyrrole).

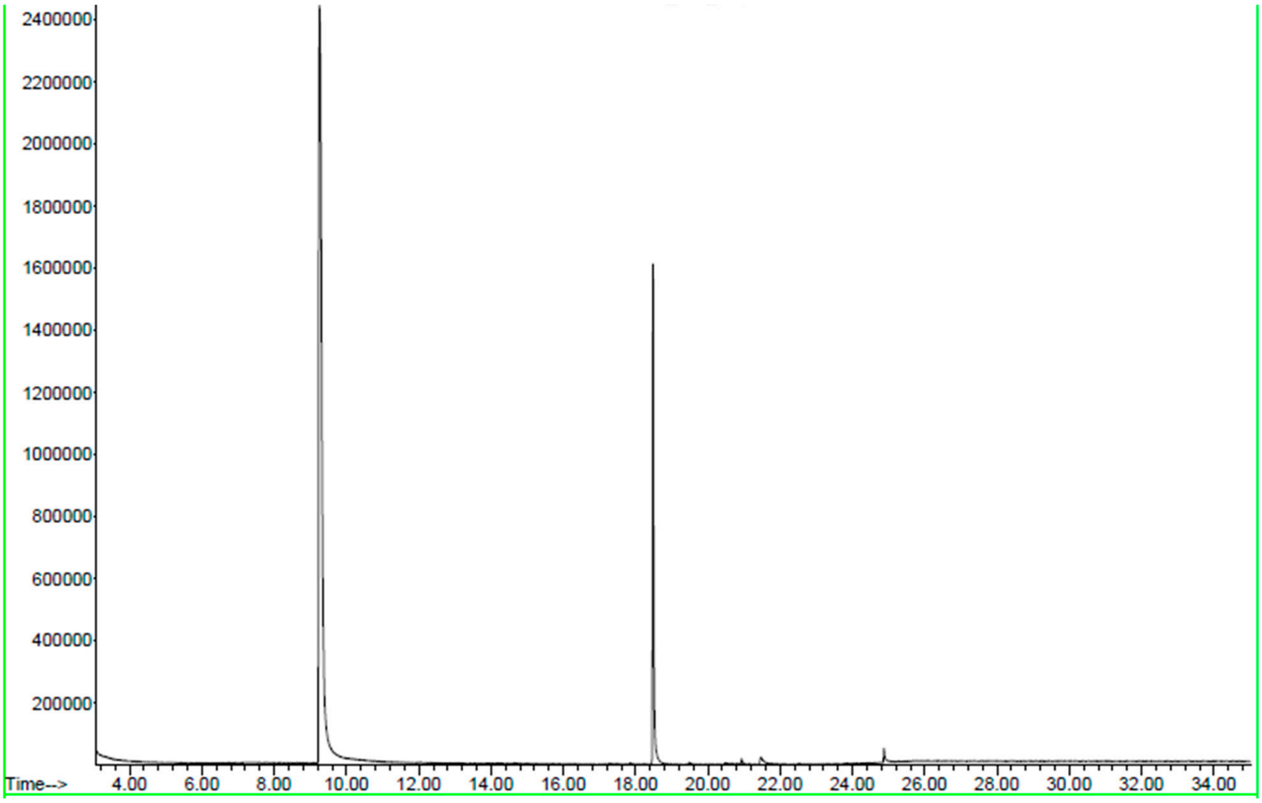

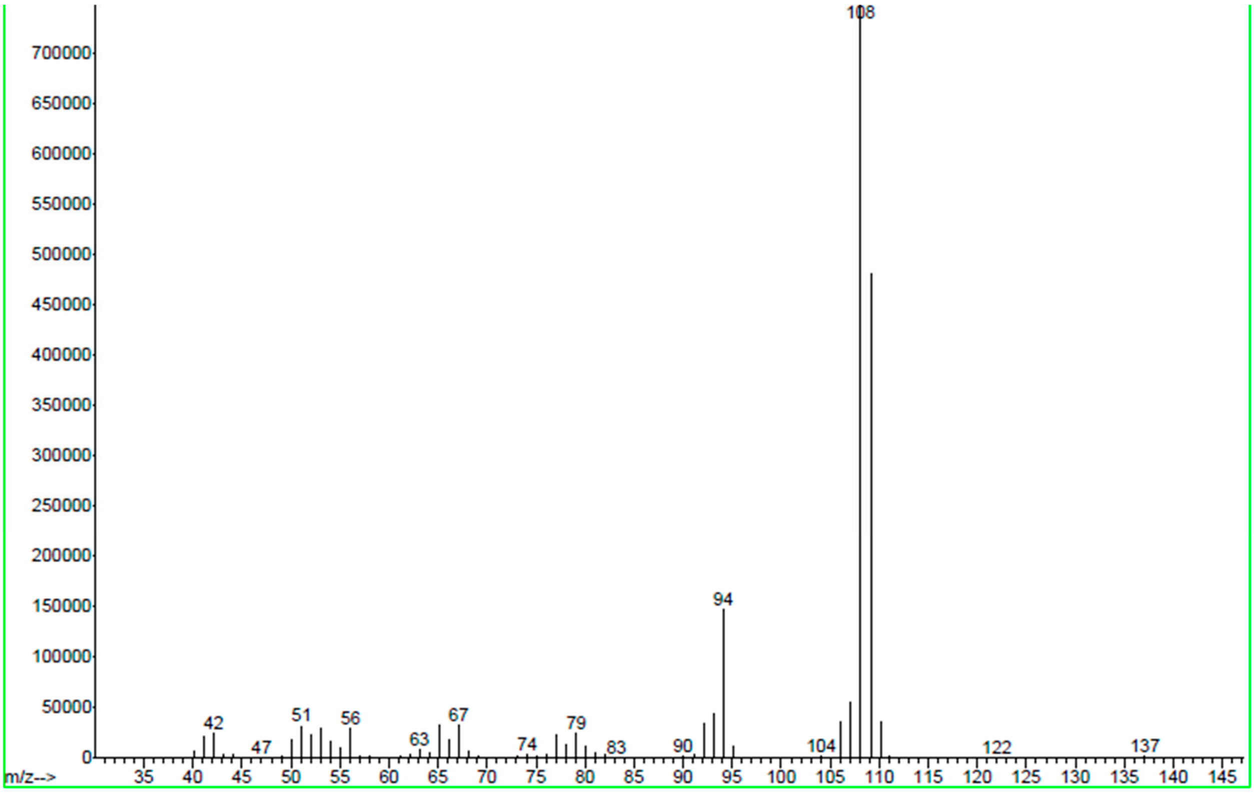

Figure S.23: GC-Mass chromatogram of trimethyl pyrrole.

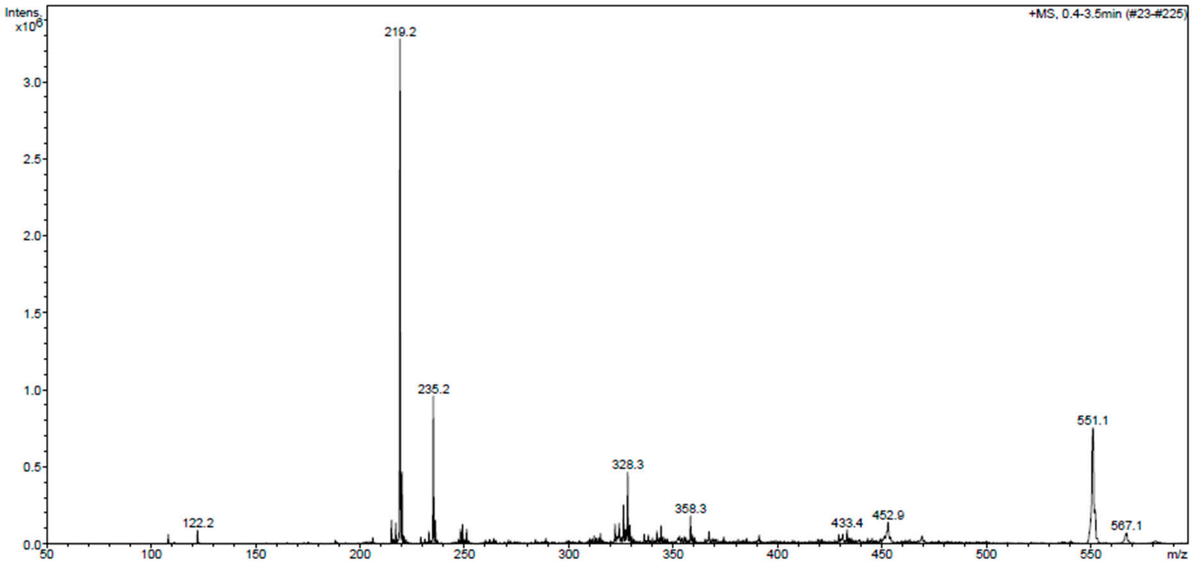

Figure S.24. Positive ESI-MS spectrum (1:100 MeOH) of trimethyl pyrrole

3e) ***1-hexyl-2,5-dimethyl-1H-pyrrole*** (hexylpyrrole).

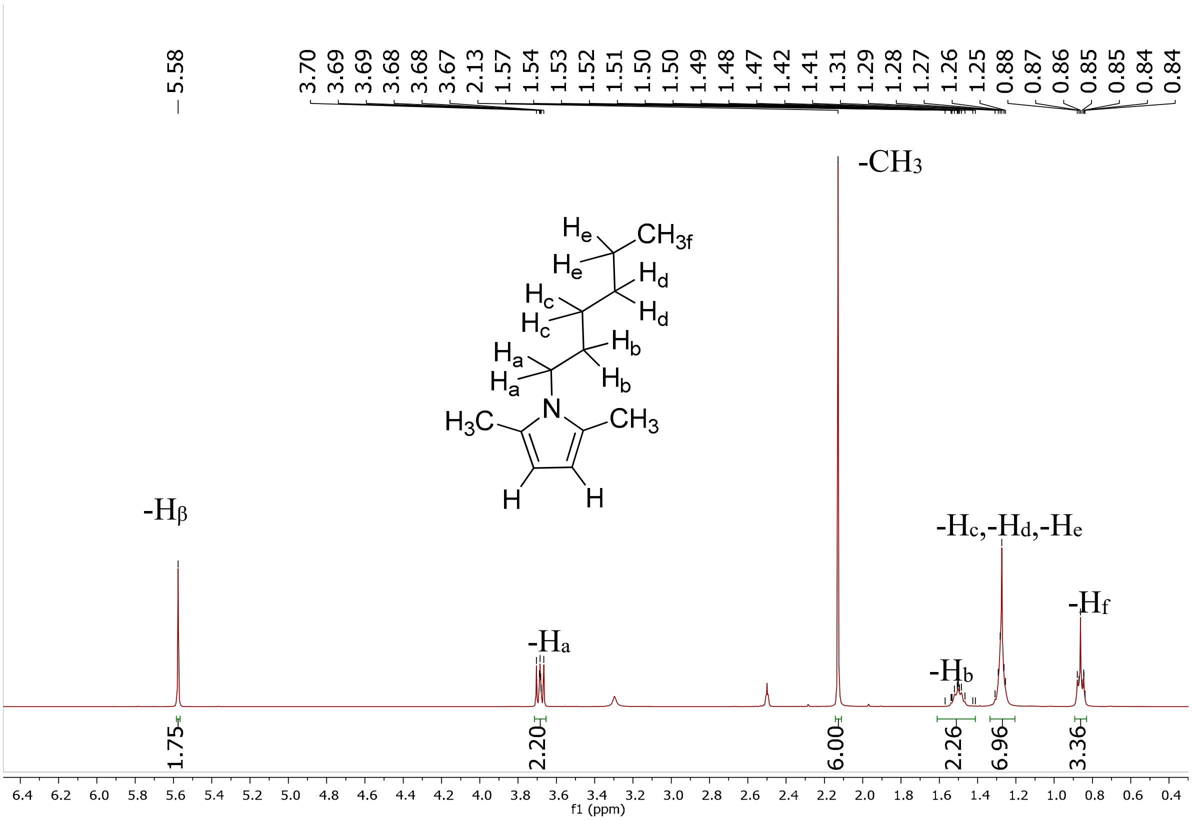

(A)

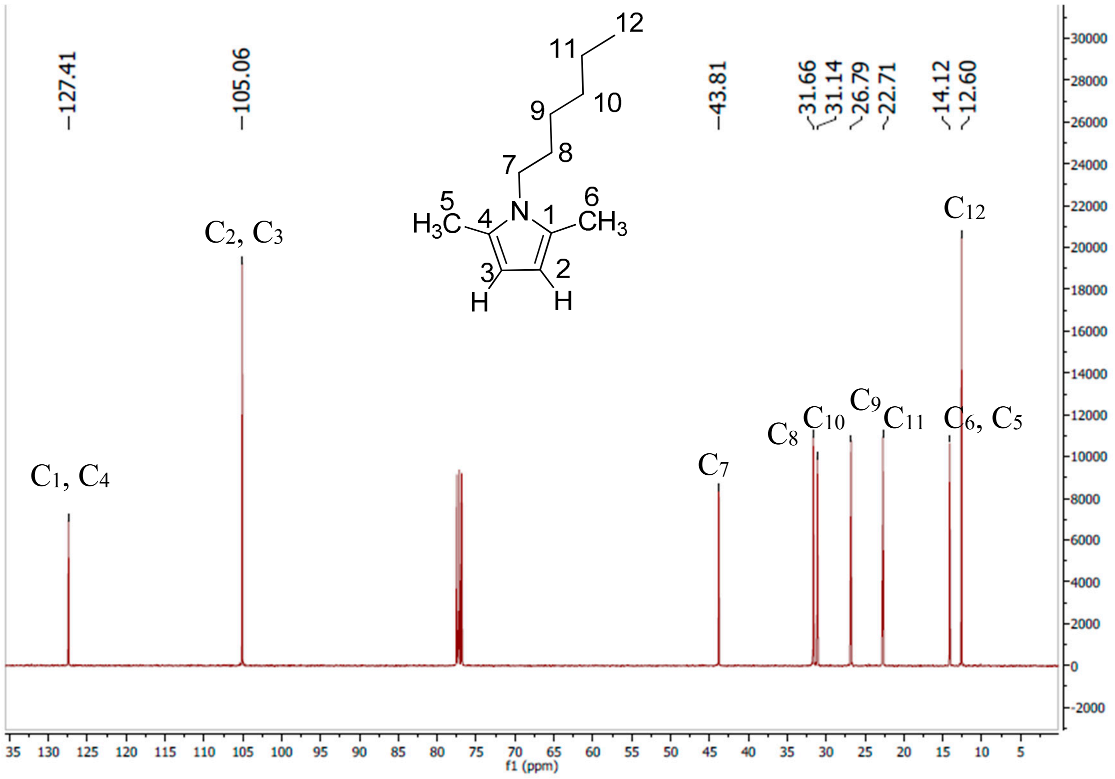

(B)

Figure S.25. NMR spectra of (A) ¹H (400 MHz, DMSO-*d*₆) and (B) ¹³C of 1-hexyl-2,5-dimethyl-1*H*-pyrrole (hexylpyrrole).

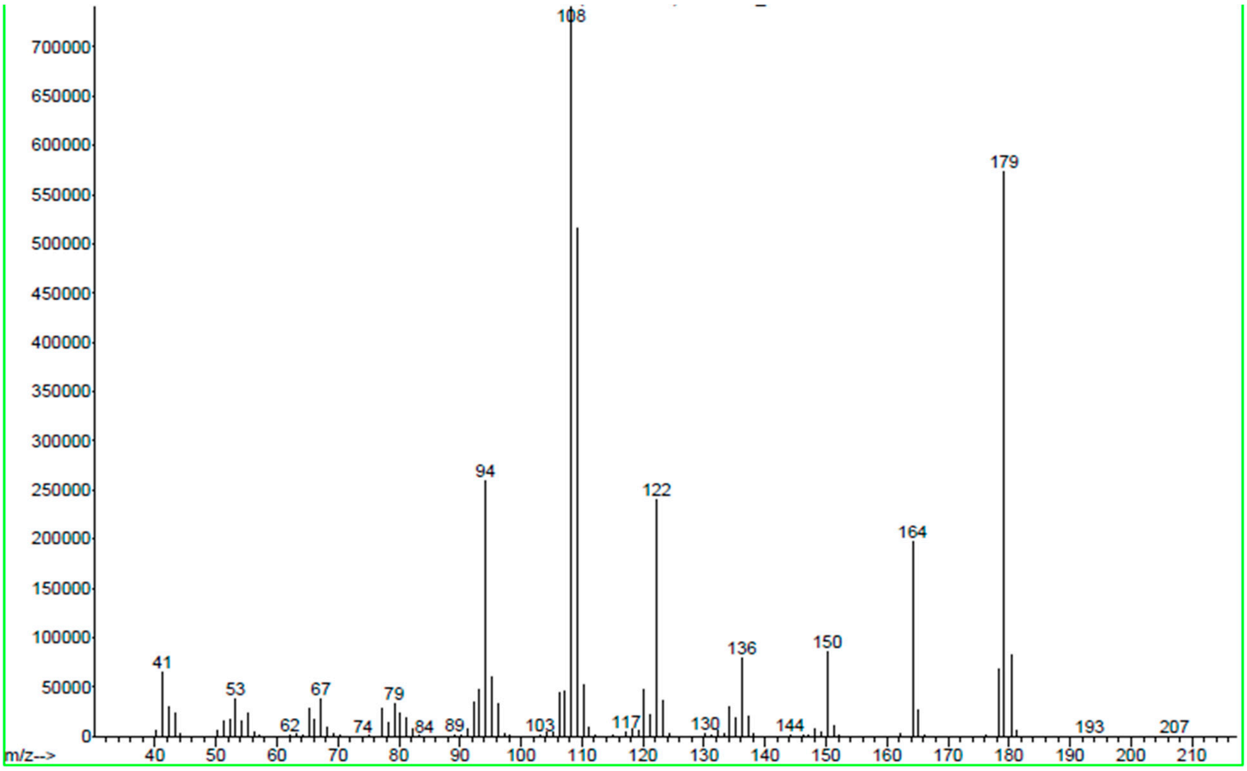

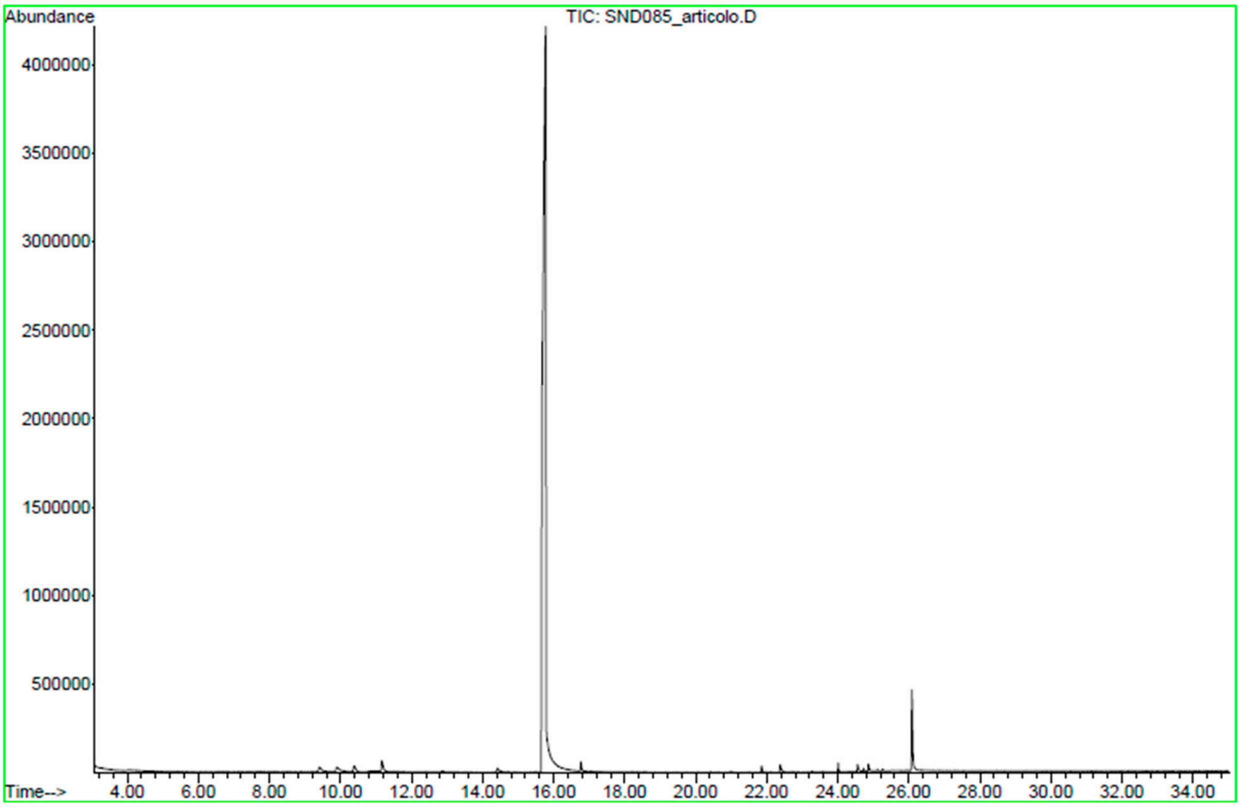

Figure S.26. GC-Mass chromatogram of 1-hexyl-2,5-dimethyl-1*H*-pyrrole

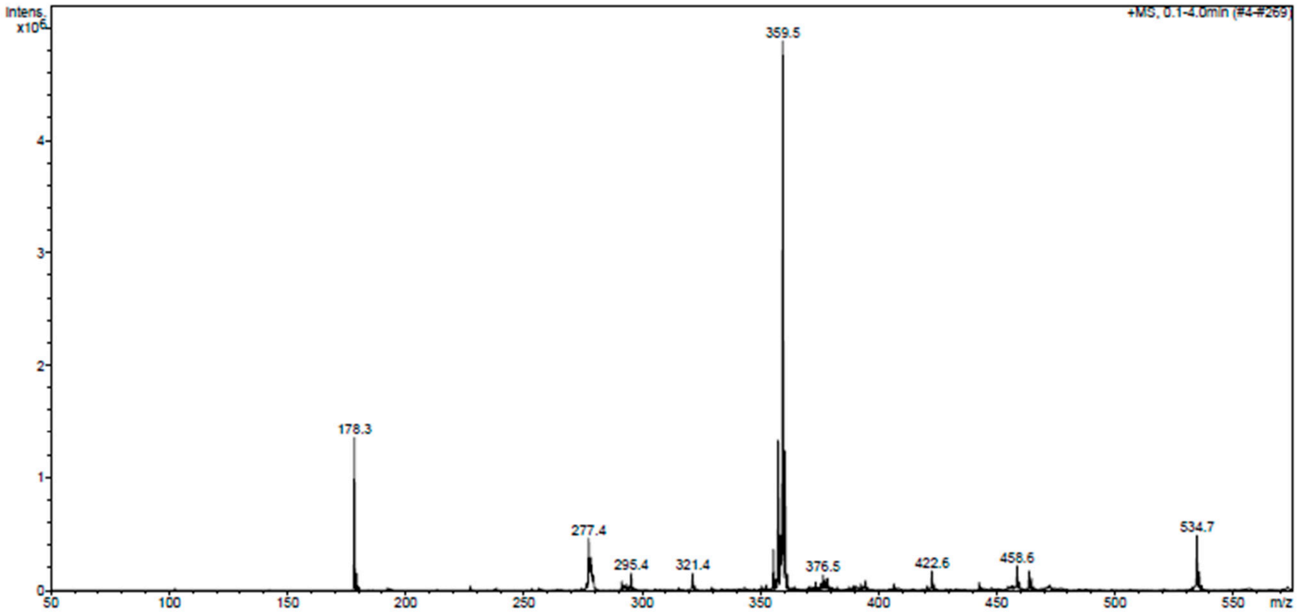

Figure S.27 Positive ESI-MS spectrum (1:100 MeOH) of 1-hexyl-2,5-dimethyl-1*H*-pyrrole

3f) 1-benzyl-2,5-dimethyl-1*H*-pyrrole (benzyl pyrrole)

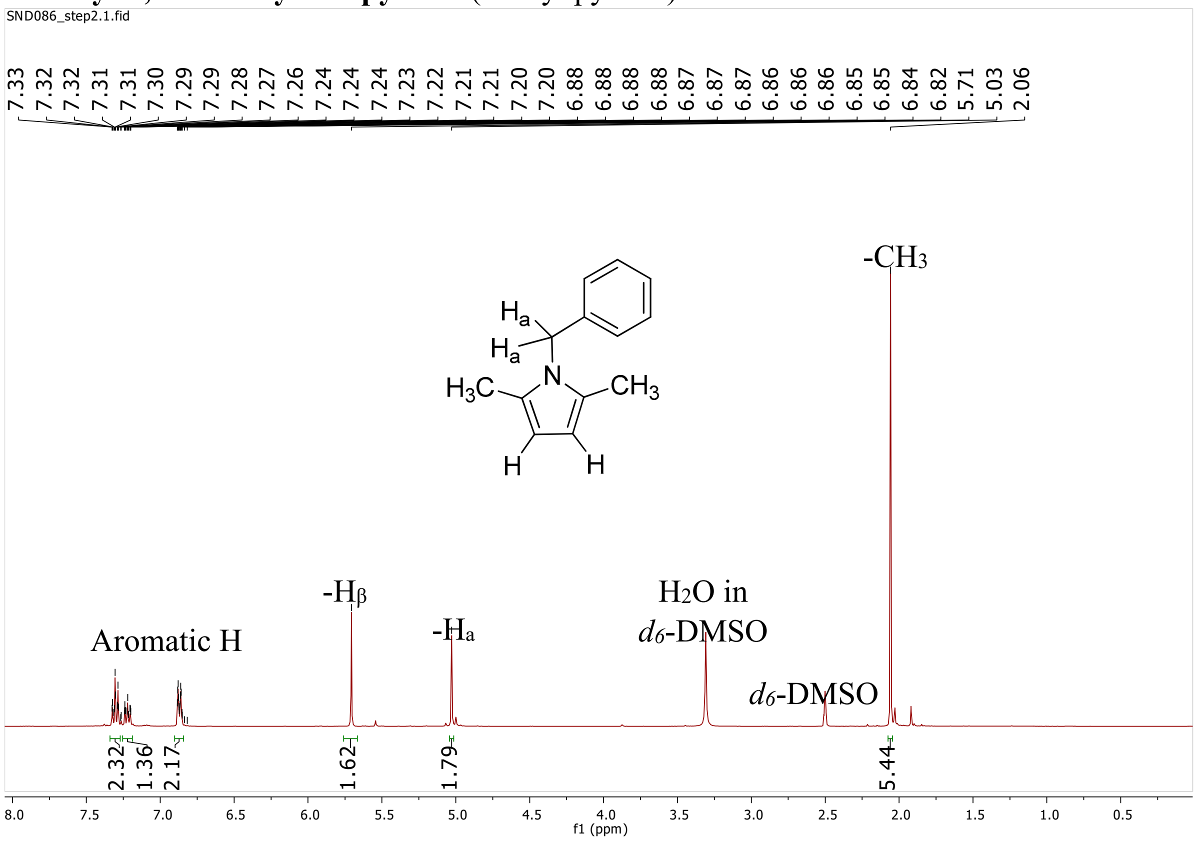

(A)

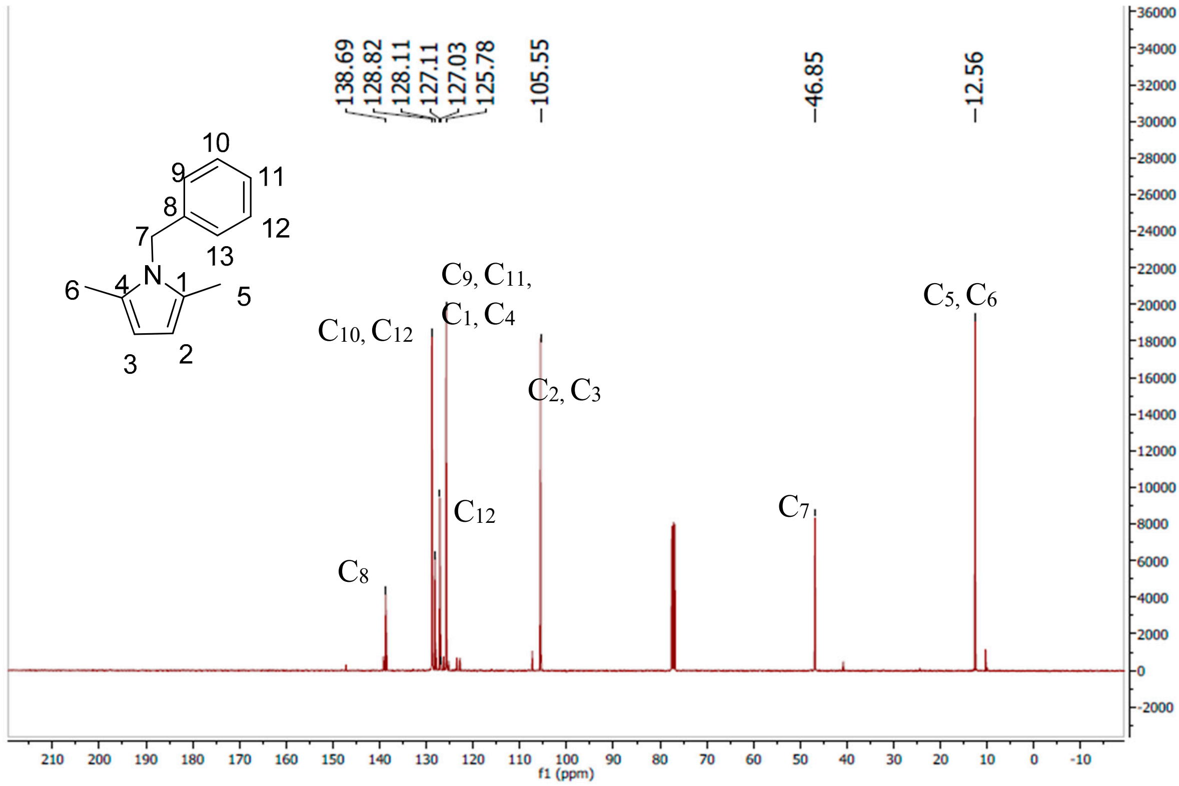

(B)

Figure S.28. NMR spectra of (A) ^{1}H (400 MHz, DMSO-*d6*) and (B) ^{13}C of 1-benzy-l,2,5-dimethyl-1*H*-pyrrole (benzyl pyrrole)

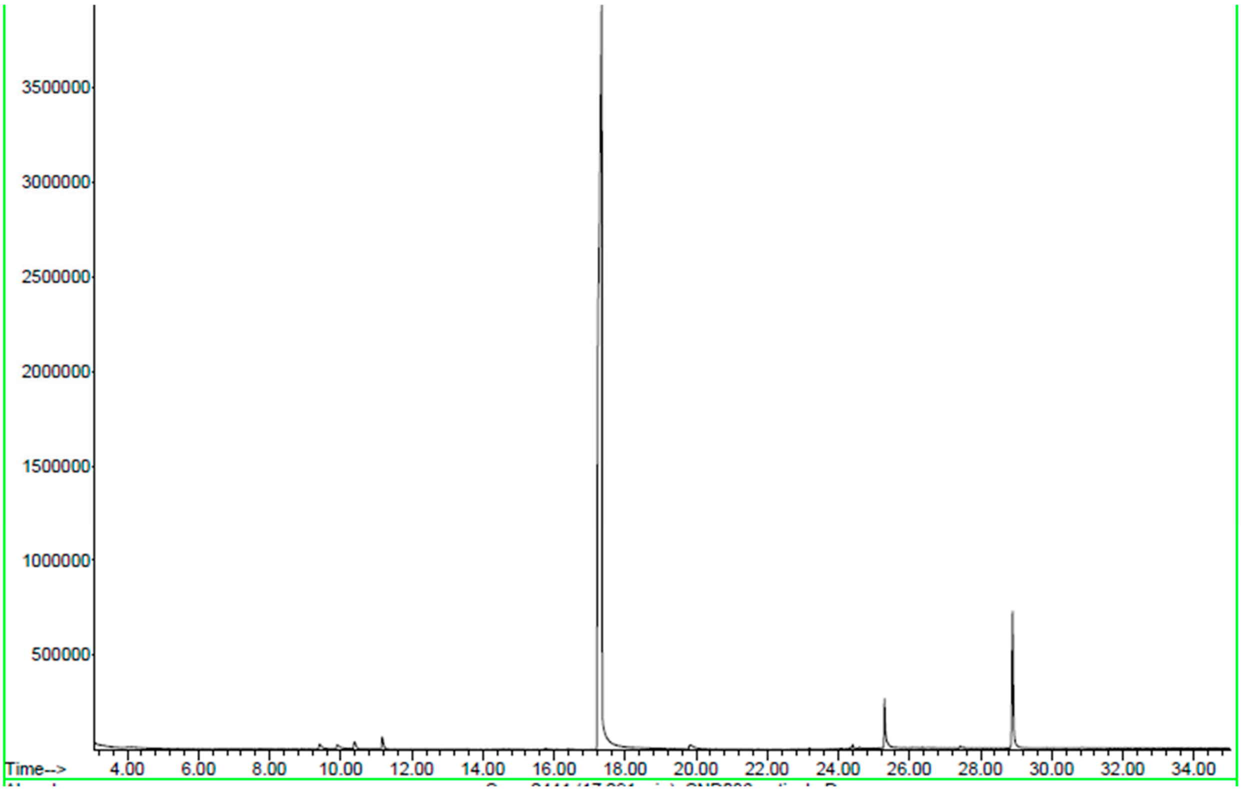

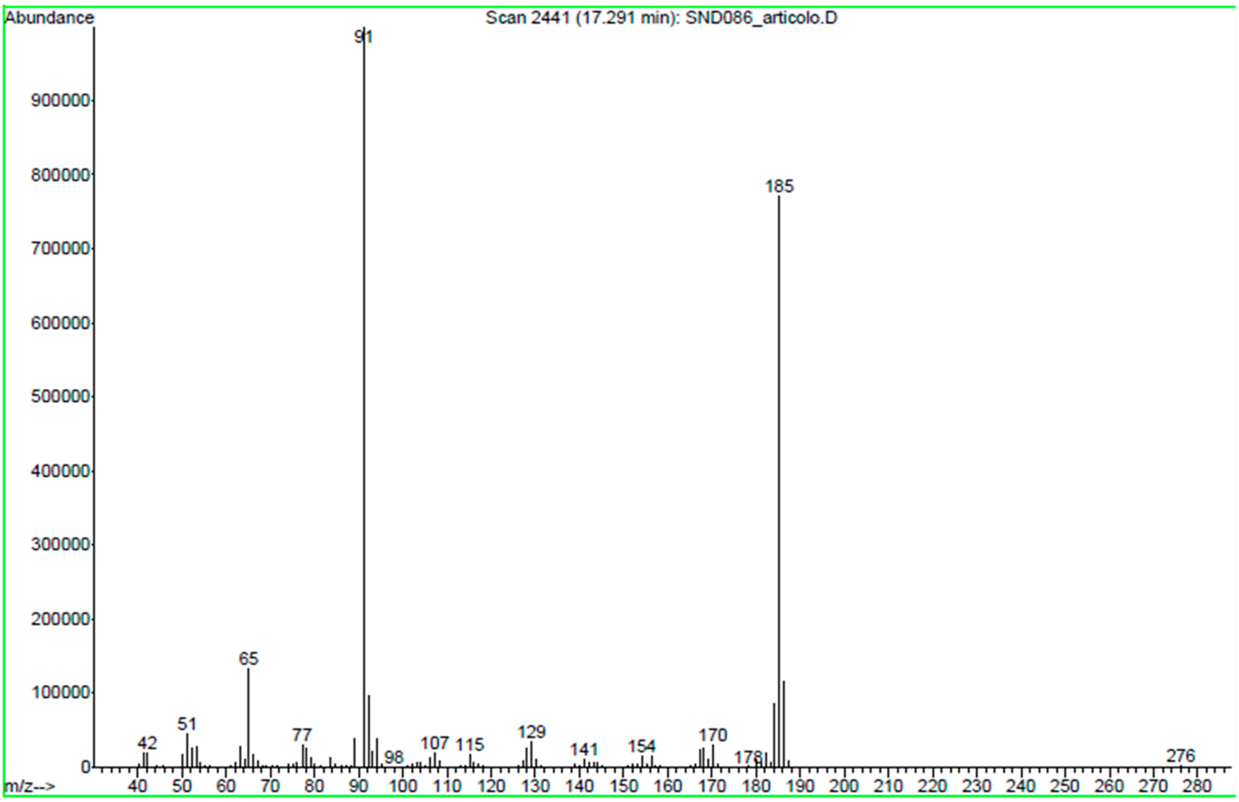

Figure S.29. GC-Mass chromatogram of 1-benzy-l,2,5-dimethyl-1*H*-pyrrole (benzyl pyrrole)

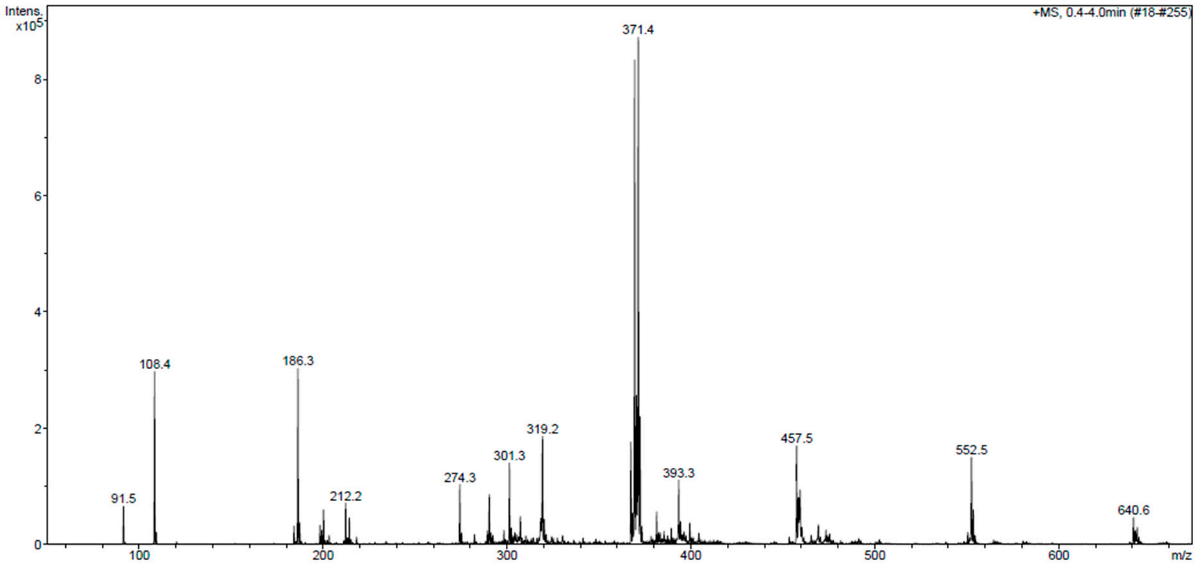

Figure S.30. Positive ESI-MS spectrum (1:100 MeOH) of 1-benzy-l,2,5-dimethyl-1*H*-pyrrole (benzyl pyrrole)

3g) **2-(2,5-dimethyl-1H-pyrrol-1-yl)ethane-1-thiol** *(SHP)*

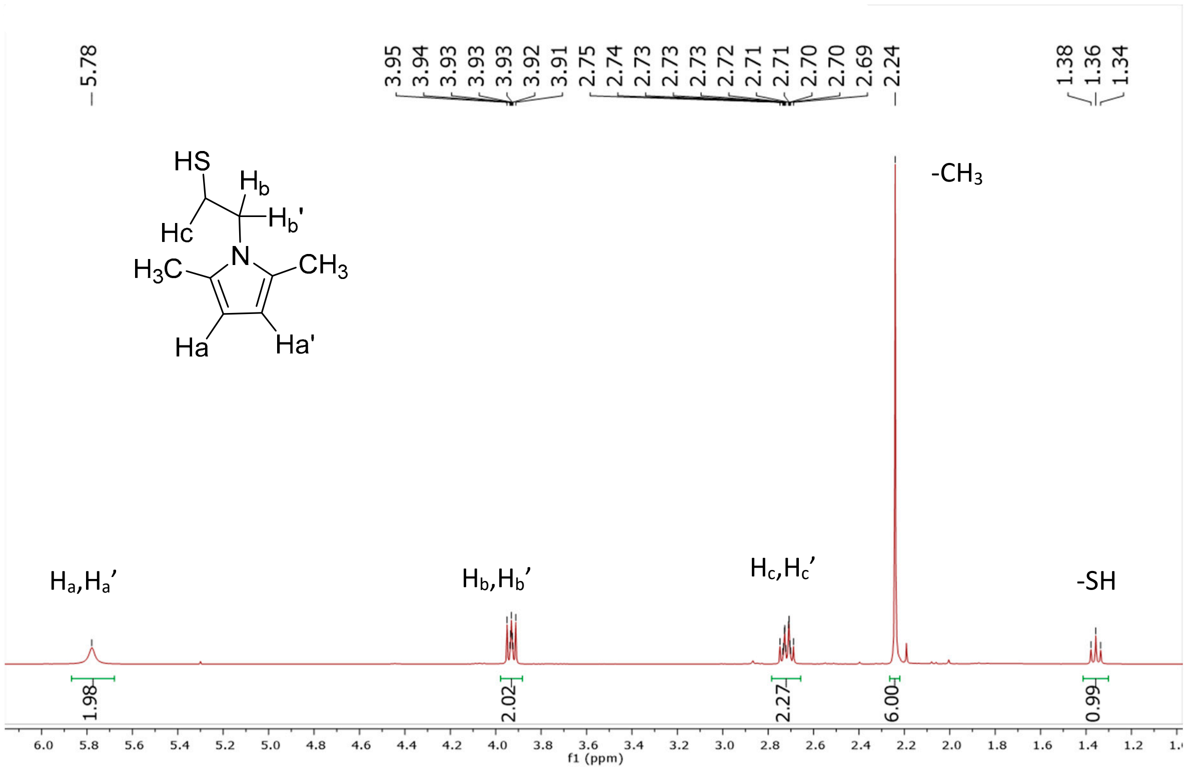

(A)

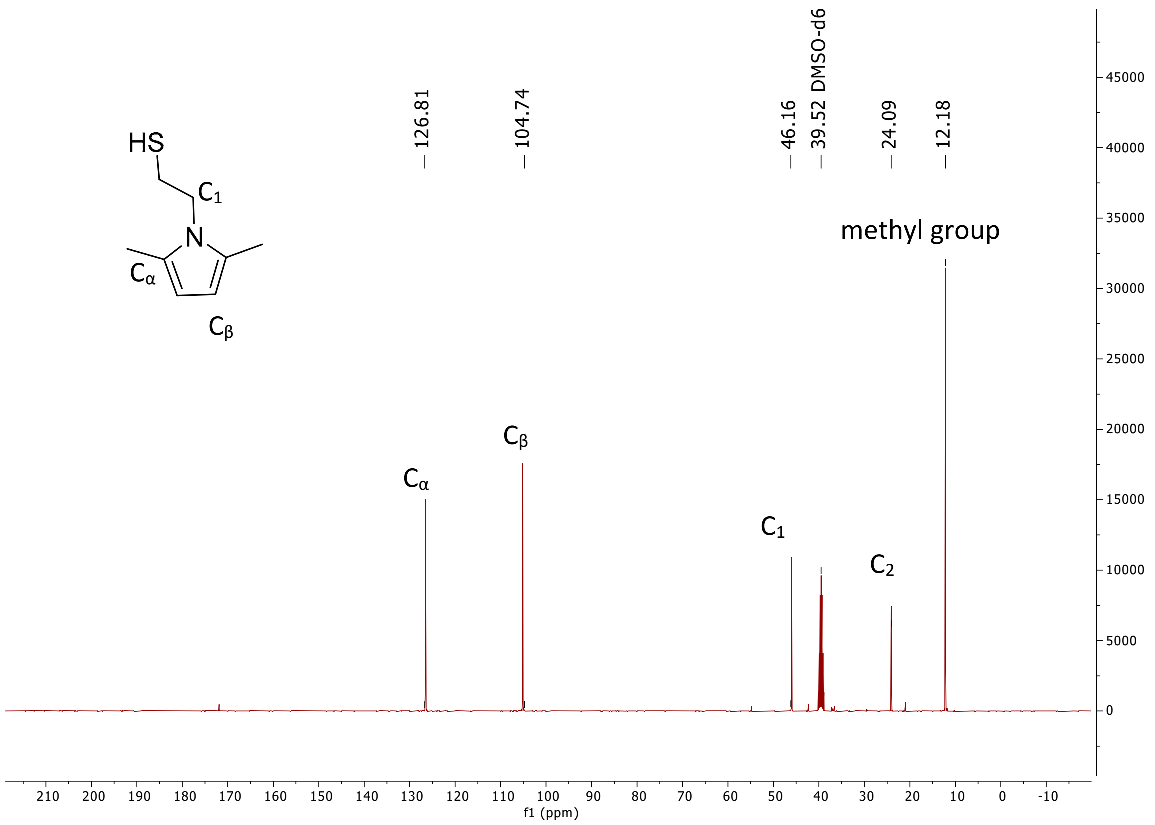

(B)

Figure S.31. (A) ^{1}H-NMR and (B) ^{13}C-NMR spectra of SHP performed in *d_6*-DMSO

GC-Mass analysis of 2-(2,5-dimethyl-1H-pyrrol-1-yl)ethane-1-thiol (SHP)

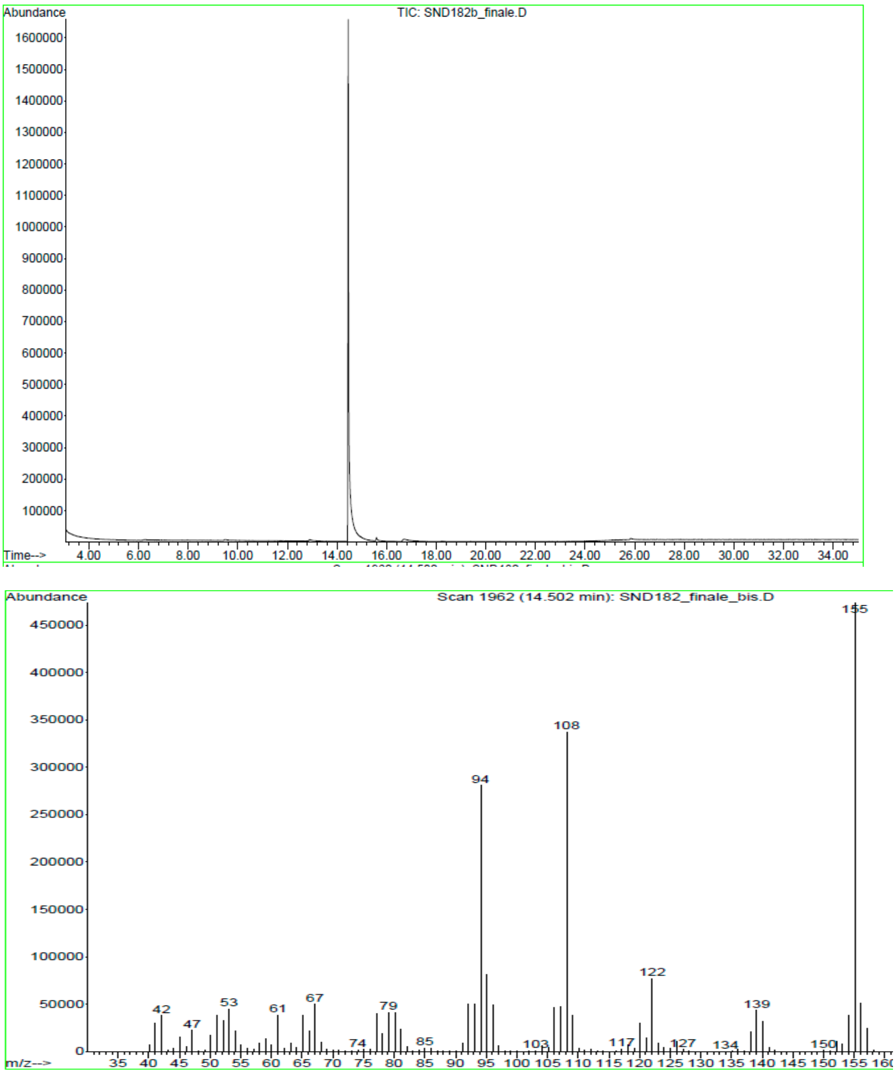

Figure S.32: GC-mass analysis of 2-(2,5-dimethyl-1H-pyrrol-1-yl)ethane-1-thiol (SHP)

3h) **1,2-bis(2-(2,5-dimethyl-1H-pyrrol-1-yl)ethyl)disulfane** (SSP)

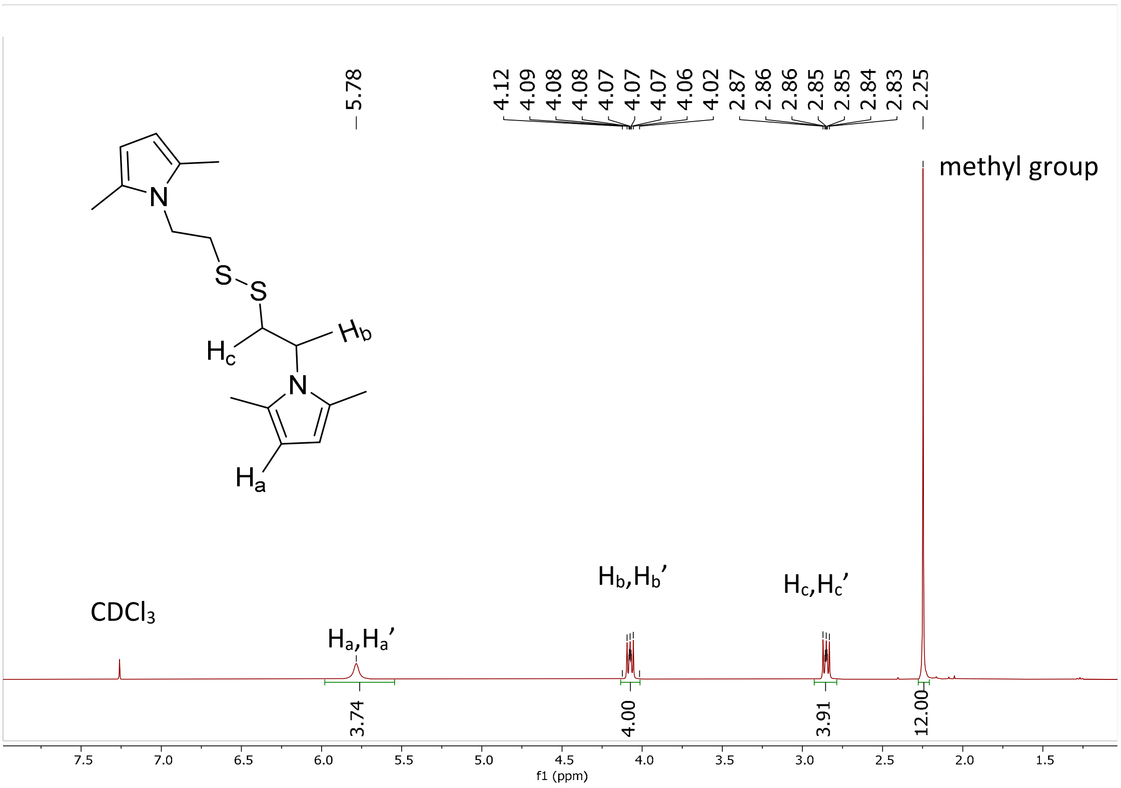

(A)

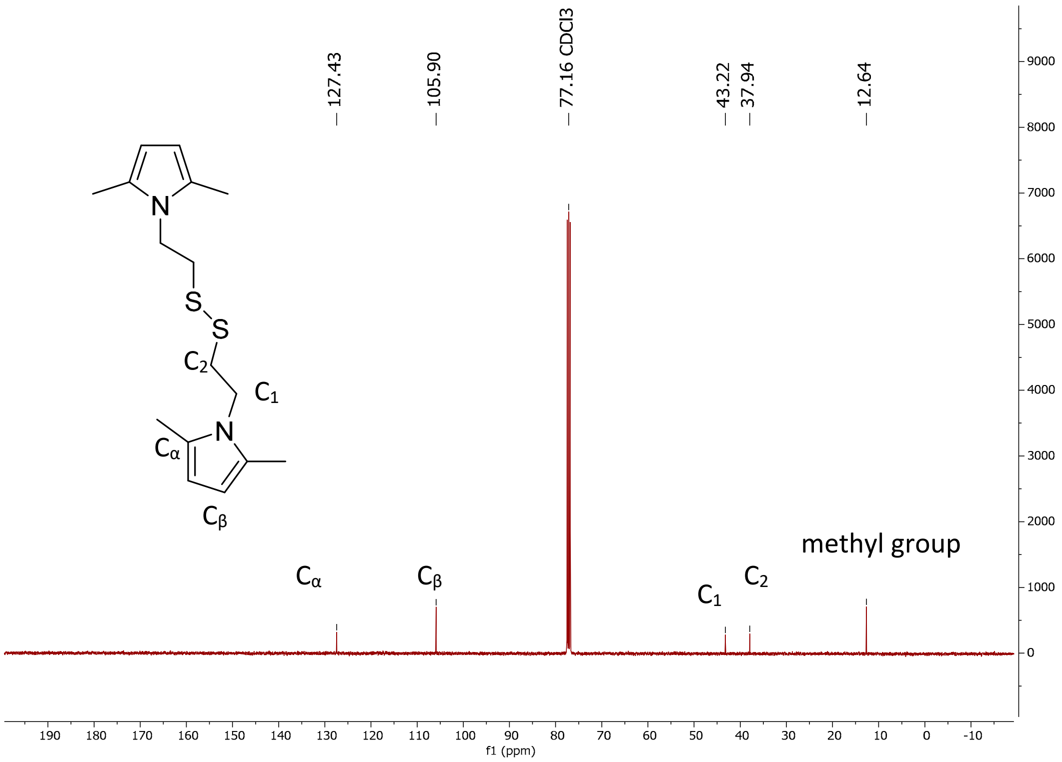

Figure S.33. (A) ^{1}H-NMR and (B) ^{13}C-NMR spectra of 1,2-bis(2-(2,5-dimethyl-1H-pyrrol-1-yl)ethyl)disulfane (SSP) performed in $CDCl_3$

GC-Mass analysis of 1,2-bis(2-(2,5-dimethyl-1H-pyrrol-1-yl)ethyl)disulfane (SSP)

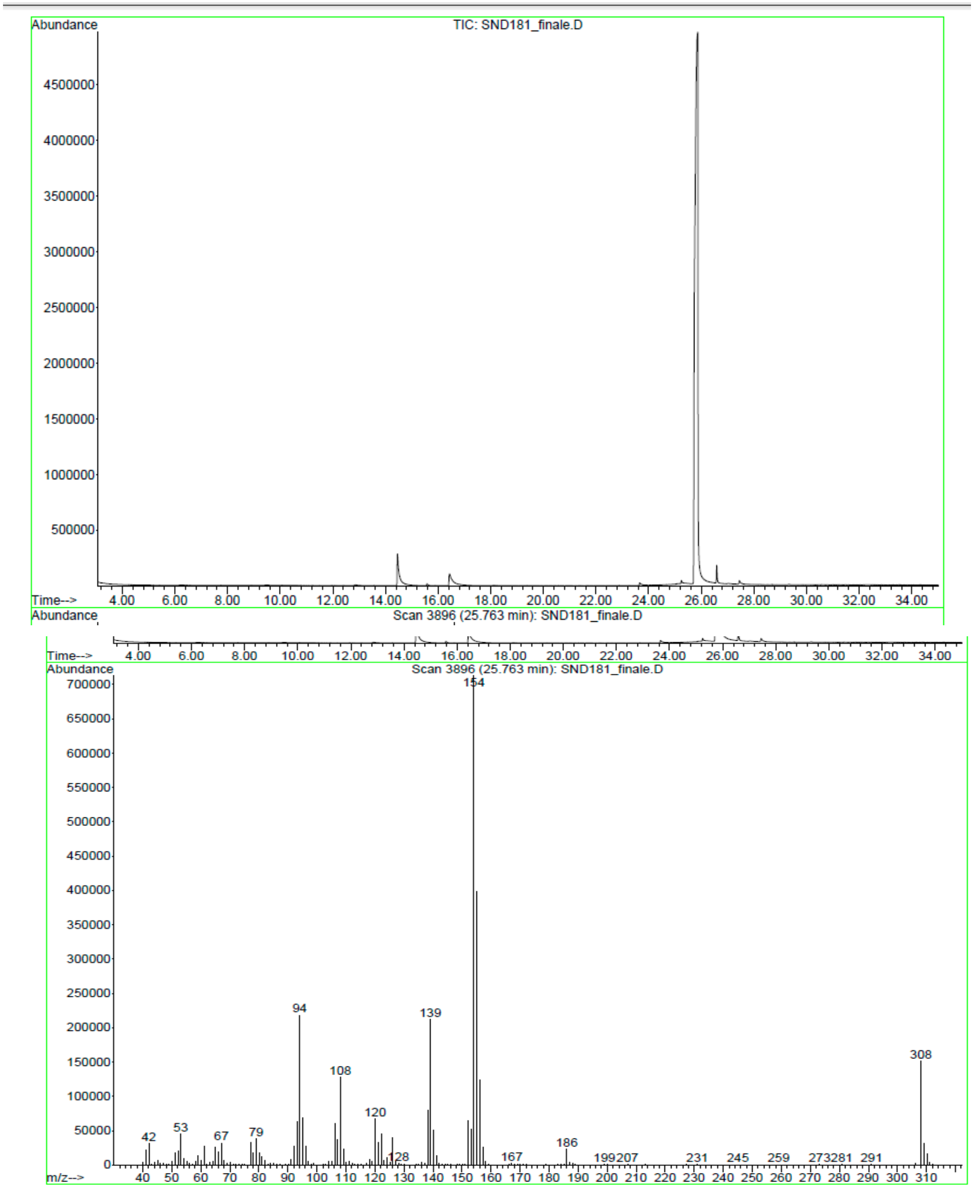

Figure S.34. GC-mass analysis of 1,2-bis(2-(2,5-dimethyl-1H-pyrrol-1-yl)ethyl)disulfane (SSP)

S6 Synthesis and characterization of SHP and SSP from

Synthesis of SHP

STEP 1: *2,5-hexanedione from 2,5-dimethylfuran*

In a round bottom flask, equipped with a magnetic stirrer, were introduced in order water (0.85 mL, 47.0 mmol), sulfuric acid (1.88 mmol, 4 mol%, 10 drops) and 2,5-dimethylfuran (5.0 ml, 47.0 mmol). The reaction mixture was heated at 50 °C and stirred for 24 hours, 400 rpm. 2,5-hexanedione was obtained, without any further purifications, as a dark brown liquid with a yield of 95%.

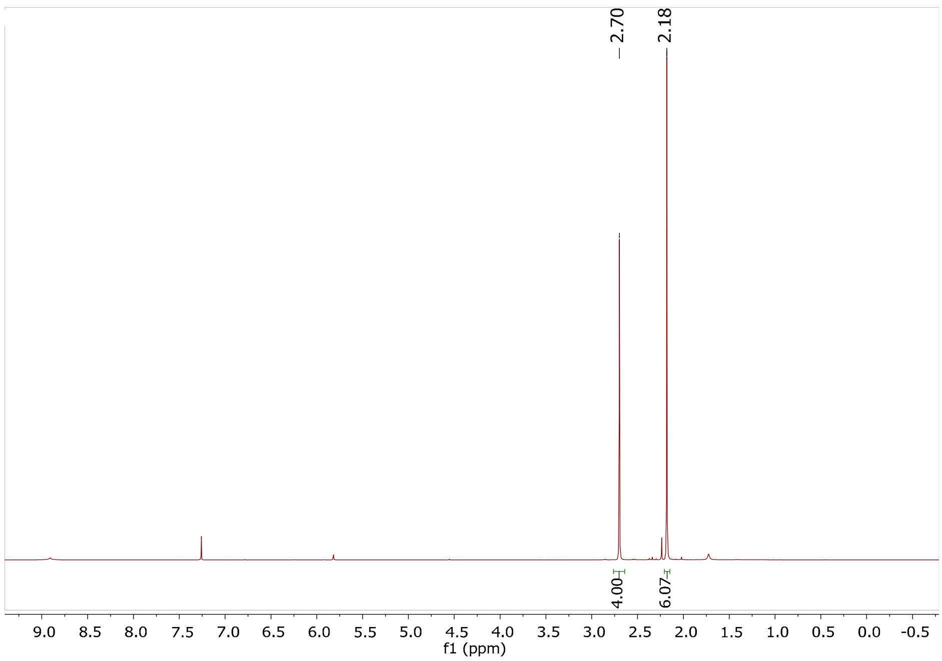

Figure S.35:^{1}H-NMR of 2,5-hexanedione starting from 2,5-dimethylfuran

STEP 2: SHP from 2,5-hexanedione

Bio-2,5-hexanedione obtained during the first step (44.65 mmol) was stirred with sodium acetate trihydrate (0.1 eq respect to 2,5-hexanedione, 4.46 mmol, 607.6 mg) at room temperature for 1 hour. In a second bottom flask, equipped with magnetic stirrer, were introduced sodium acetate trihydrate (6,075 g, 44.65 mmol) and cisteamine hydrochloride (5,072 g, 44.65 mmol); the mixture was stirred at room temperature since an homogeneous solution was obtained.

Then, the solution of bio-2,5-hexanedione and sodium acetate trihydrate was added to the solution of cisteamine hydrochloride dropwise. The resulting solution was stirred at room temperature for 4 hours, 400 rpm.

Neutralization:

A satured solution of NaHCO3 was added to the mixture, under stirring, until the end of the effervescence.

WORK-UP:

Then, the organic phase was extracted with dicloromethane and anhydrified with Na2SO4. The solvent was removed under reducted pression to rotavapor.

SHP was obtained as a dark brown liquid.

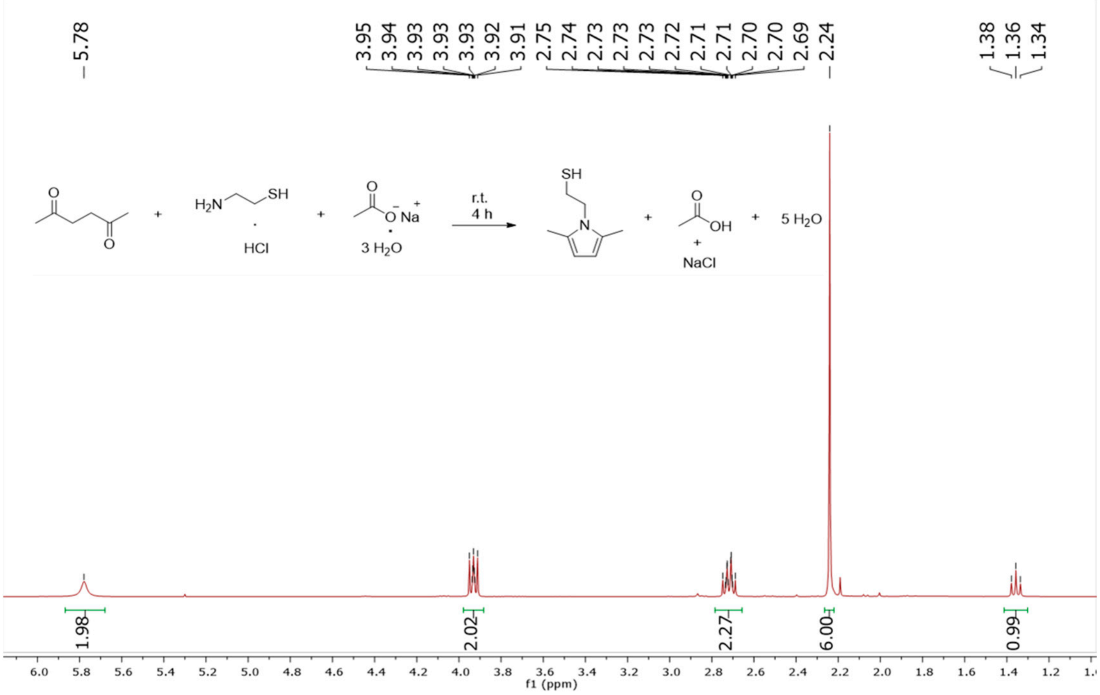

Figure S.36:^{1}H-NMR of SHP starting from obtained by using cysteamine hydrochloride as the hydrochloric salt

Synthesis of SSP

STEP 1: **2,5-hexanedione from 2,5-dimethylfuran**

In a round bottom flask, equipped with a magnetic stirrer, were introduced in order water (0.85 mL, 47.0 mmol), sulfuric acid (1.88 mmol, 4 mol%, 10 drops) and 2,5-dimethylfuran (5.0 mL, 47.0 mmol). The reaction mixture was heated at 50 °C and stirred for 24 hours, 400 rpm. 2,5-hexanedione was obtained, without any further purifications, as a dark brown liquid.

STEP 2: SSP from 2,5-hexanedione

Bio-2,5-hexanedione obtained during the first step was stirred with sodium acetate trihydrate (0.1 eq. respect to 2,5-hexanedione, 4.46 mmol, 607.6 mg) at 100 °C for 1 hour.

In a second bottom flask, equipped with magnetic stirrer, were introduced sodium acetate trihydrate (6,075 g, 44.65 mmol) and cistamine dihydrochloride (5,072 g, 22.32 mmol); the solution was stirred at 100 °C since an homogeneous solution was obtained.

Then, the solution of bio-2,5-hexanedione was added to the solution of cistamine dihydrochloride dropwise. The resulting solution was stirred at 100 °C for 2 hours, 400 rpm.

NEUTRALIZATION

A satured solution of NaHCO3 was added to the mixture, under stirring, until the end of the effervescence.

WORK-UP:

Then, the organic phase was extracted with ethyl acetate and anhydrified with Na2SO4; the solvent was removed under reducted pression.

SSP was obtained as a dark orange solid.

^{1}H-NMR confirmed the result.

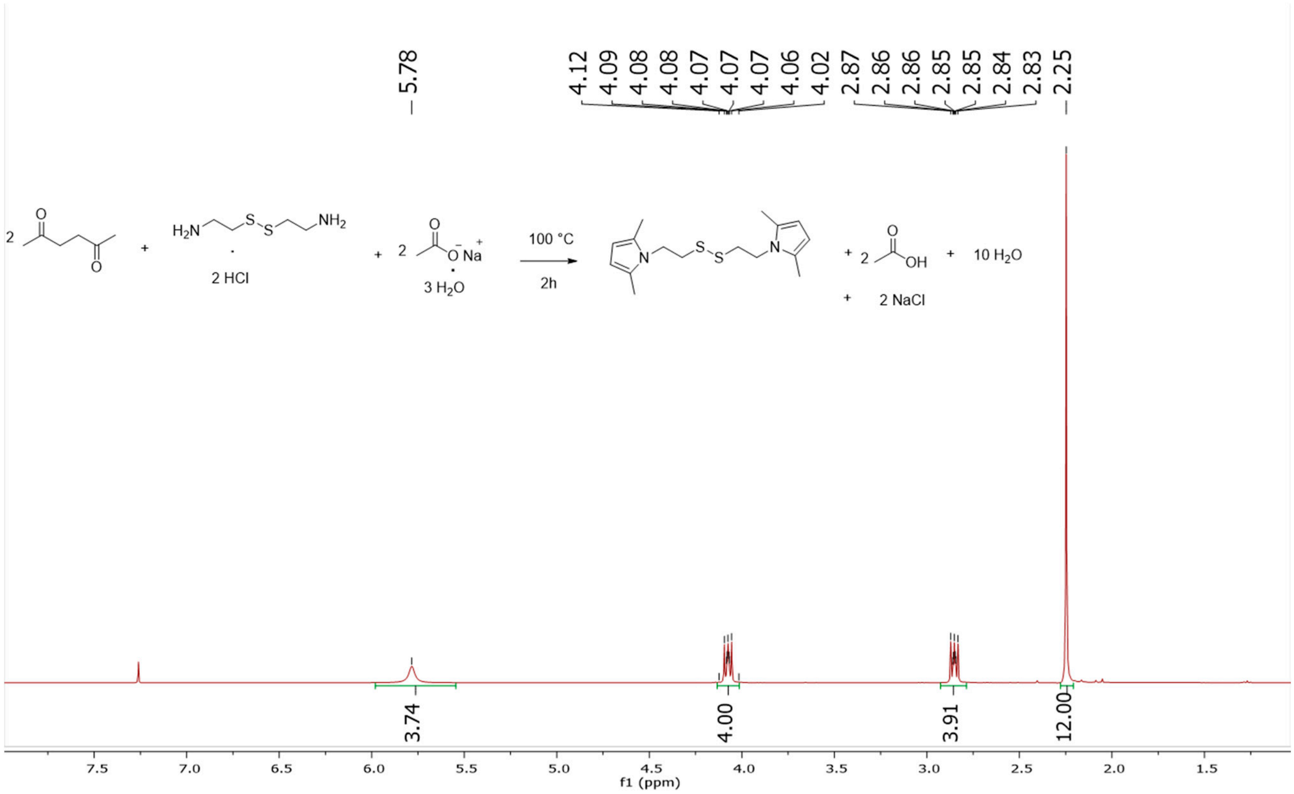

Figure S.37: ^{1}H-NMR of SSP starting from obtained by using cistamine dihydrochloride ad the hydrochloric salt

S7 Green metrics calculation for bio-pyrroles

Green metrics parameters are implemented by using the definition reported in references 56 and 57.

Atom economy.

The atom economy gives the indication of the reagents' atoms which are converted into the desired product, without considering the yield of the reaction.

Atom economy is calculated through the Equation 1 below:

AEc (%) = (Molecular Mass of the product)/(∑ 〖Molecular Mass of all reagents〗)*100 Eq. 1

Atom efficiency. The atom efficiency considers the yield of the reaction and is calculated through Equation 2 below:

AEf (%) = AEc ∗ yield Eq. 2

Carbon economy

Carbon economy is calculated through the Equation 3 below:

CEc (%) = (Molar Mass of C atoms the product)/(∑▒ 〖Molar Mass of C in the all reagents)*100 Eq.3

Carbon efficiency

The Carbon Efficiency (CEf) was calculated through the Equation 4 below and indicates the actual amount of carbon in the product:

CEf (%) = CEc ∗ yield Eq. 4

Reaction Mass efficiency

The Reaction Mass Efficiency (RME) was calculated through the equation 5 below.

RME = (Mass of the product)/(∑▒ 〖 Mass of the all reagents〗)*100………………Eq. 5

E factor

The E-factor takes into account all the materials used to perform the chemical reactions, is defined as the ratio of the mass of waste per mass of product and was calculated through the Equation 6 below:

E-Factor = (Mass of Waste)/(Mass of Product) Eq. 6

Reference for the Green metrics calculation are in the main text [49-51,66,67].

S8. Rheometric data

Table S1: Properties from the vulcanization of composites in Table 4.

	Composite based on			
**Property**	**Silica**	**CB/SHP**	**CB + S**	**CB**
M_L (dNm)	4.33	3.83	2.56	2.60
M_H (dNm)	24.44	19.00	18.68	16.46
M_H-M_L (dNm)	20.11	15.17	16.12	13.86
t_{S1} (min)	2.01	3.03	3.31	3.56
t_{90} (min)	10.00	6.76	8.17	8.79
(M_H-M_L) / (t_{90}-t_{S1}) (dNm/min)	2.52	3.84	3.32	2.65

Table S2: $G'\gamma_{min}$, $G'\gamma_{max}$, $\Delta G'$, $\Delta G'/G'\gamma_{min}$, G''_{max} and Tan δ_{max} from strain sweep tests on composites of Table 4.

	Composite based on			
**Property**	**Silica**	**CB/SHP**	**CB + S**	**CB**
$G'\gamma_{min}$ (MPa)	4.68	2.67	4.79	3.60
$G'\gamma_{max}$ (MPa)	1.53	1.33	1.18	1.08
$\Delta G'$ (MPa)	3.15	1.34	3.61	2.52
$\Delta G'$ / $G'\gamma_{min}$	0.67	0.50	0.75	0.60
G''_{max} (MPa)	0.40	0.28	0.55	0.52
Tan δ_{max}	0.18	0.16	0.27	0.28
